# Enantioselective Synthesis of α‐Thiocarboxylic Acids by Nitrilase Biocatalysed Dynamic Kinetic Resolution of α‐Thionitriles

**DOI:** 10.1002/chem.202001108

**Published:** 2020-07-16

**Authors:** Kate Lauder, Silvia Anselmi, James D. Finnigan, Yuyin Qi, Simon J. Charnock, Daniele Castagnolo

**Affiliations:** ^1^ School of Cancer and Pharmaceutical Sciences King's College London 150 Stamford Street SE1 9NH London UK; ^2^ Prozomix Limited West End Industrial Estate, Haltwhistle Northumberland NE49 9HA UK

**Keywords:** biocatalysis, dynamic kinetic resolution, nitrilase, thiocarboxylic acid, thionitrile

## Abstract

The enantioselective synthesis of α‐thiocarboxylic acids by biocatalytic dynamic kinetic resolution (DKR) of nitrile precursors exploiting nitrilase enzymes is described. A panel of 35 nitrilase biocatalysts were screened and enzymes Nit27 and Nit34 were found to catalyse the DKR of racemic α‐thionitriles under mild conditions, affording the corresponding carboxylic acids with high conversions and good‐to‐excellent *ee*. The ammonia produced in situ during the biocatalytic transformation favours the racemization of the nitrile enantiomers and, in turn, the DKR without the need of any external additive base.

## Graphical abstract

**Nitrilase biocatalysts** catalyse the dynamic kinetic resolution (DKR) of α‐thionitriles affording enantiomerically pure α‐thiocarboxylic acids under mild conditions. The DKR is favoured by the ammonia formed in situ during the biotransformation.

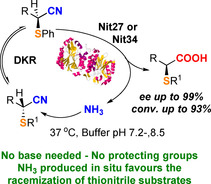

Enantiomerically pure α‐mercapto(thio)carboxylic acids are ubiquitous structural motifs found in pharmaceutical ingredients,^1^ peptides,^2^ metal chelators^3^ and S,O‐ligands^4^ or used as precursors in the asymmetric synthesis of drugs like tiopronin **2** and the IMP1 inhibitor **3** (Figure 1).^5^ Mercapto/thio‐carboxylic acids also belong to the class of volatile sulphur compounds (VSC) and found application as flavouring and aroma agents in food and fragrance industries.^6^ Compounds such as **4** and **5** have a characteristic meaty‐cheese aroma or fruity/tropical flavour, respectively and these organoleptic properties are related to the configuration of their C−S stereocentre. Despite the key importance of chiral α‐thiocarboxylic acids, the available and efficient protocols for their asymmetric synthesis are limited. In fact, the construction of molecules bearing a stereodefined C−S centre poses a distinct challenge in organic chemistry mainly because sulphur compounds have different reactivity which can be incompatible with the type of asymmetric chemistry exhibited by their O‐ or N‐derivative analogues. The most straightforward methods to access enantiomerically pure α‐thiocarboxylic acids rely on stereospecific substitution reactions of enantioenriched halo‐,^7^ hydroxy‐ or amino acid^8^ derivatives with sulphur nucleophiles. Enzymatic and non‐enzymatic dynamic kinetic resolution (DKR) also represents an efficient approach to access enantioenriched α‐thiocarboxylic acids from esters, due to the ability of a C−S stereocentre in alpha to an electron withdrawing group to racemise under specific conditions. Examples of DKR of thioesters using hydrolase enzymes9a, 9b, 9c or thiocarboxylic acids using *Nocardia diaphanozonaria* cells9d have been reported by Drueckhammer and Otha, respectively, while Birman described a non‐enzymatic DKR of α‐thiocarboxylic acids using homo‐benzotetramisole (*S*)‐HBTM.^10^ However, despite the excellent conversion and selectivity, all these methods show some limitations in terms of green chemistry, such as the need of additives and bases (*i*Pr_2_NEt or trioctylamine) to promote the dynamic racemization of the substrates, the use of ester protecting groups with consequent production of waste in the chemical process and the need of oxygen‐free conditions to avoid the formation of side products.

**Figure 1:**
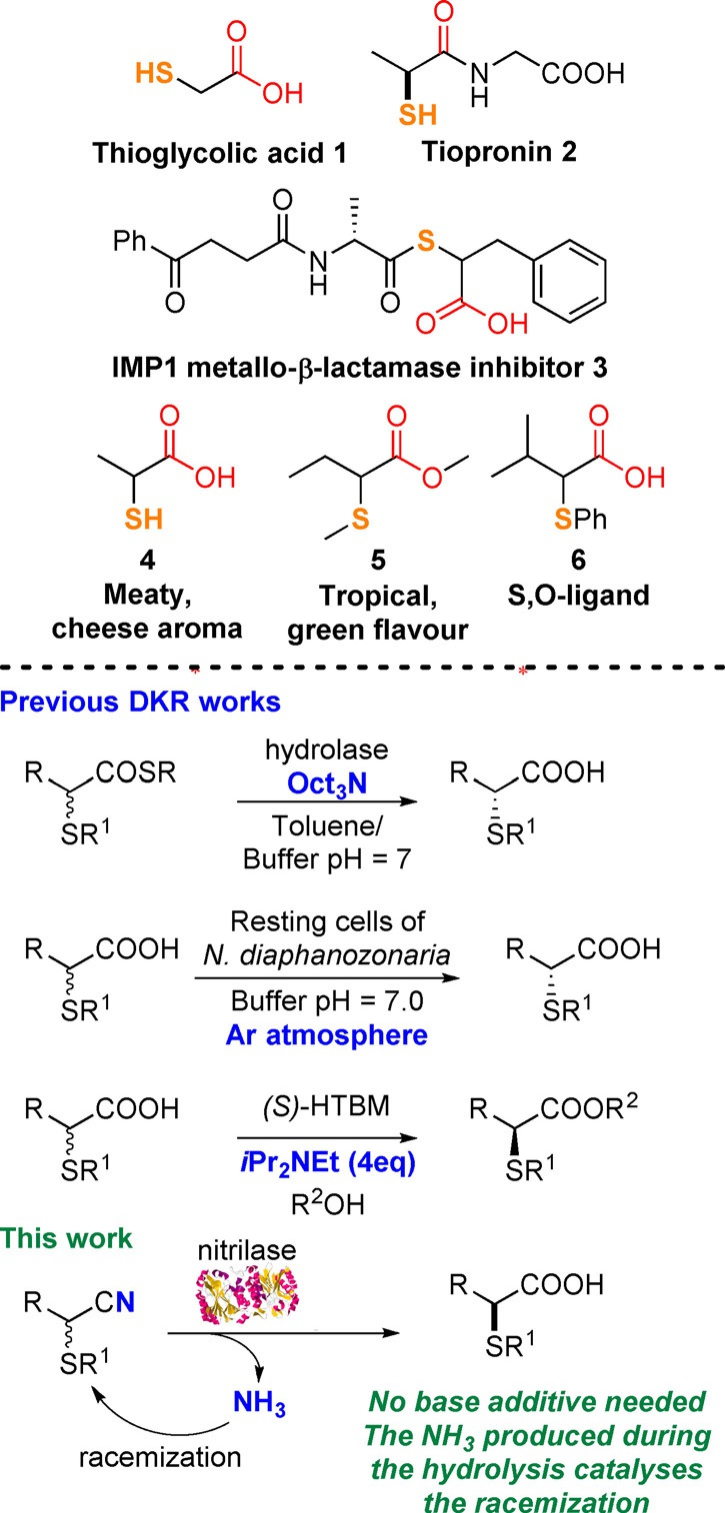
Examples of α‐thio carboxylic acids and aim of the work.

Due to our interest in the development of green and sustainable biocatalytic methodologies to access VSC and drug‐like compounds,^11^ herein we describe a DKR approach to synthesise enantiomerically pure α‐thiocarboxylic acids from racemic α‐thionitriles exploiting nitrilase enzymes. Nitrilases^12^ catalyse the hydrolysis of nitriles into the corresponding carboxylic acids with the formation of ammonia as side product. We hypothesised that the NH_3_ produced in situ during the course of the biotransformation would be sufficient to promote the racemization of the thionitrile substrates and their DKR, as shown in Figure 1, without the need of additional external bases to the reaction mixture or the use of protecting groups.

In addition, the enzymatic hydrolysis would allow the efficient synthesis of α‐thiocarboxylic acids under mild conditions. In fact, even if it is widely documented that nitriles can be hydrolysed into carboxylic acids under acidic or basic conditions,^13^ we found that α‐thionitriles decompose into volatile by‐products in the presence of strong acids/bases,^14^ thus supporting the need for a mild methodology to access α‐thiocarboxylic acids.

The racemic thionitrile **8 a**, synthesised from the bromonitrile **7** under microwave irradiation on water (Scheme 1), was chosen as a model substrate to identify the most appropriate hydrolytic biocatalysts from a pool of 35 nitrilases, identified and isolated through a metagenomic approach from Prozomix's library. A colourimetric assay,12d, 15 able to detect the NH_3_ formed during the nitrile hydrolysis reaction by exploiting *o*‐phthalaldehyde (OPA), was carried out, leading to the identification of eight hit nitrilases (blue/dark wells in Scheme 1). The conversion and enantiomeric excess (*ee*) for each of the 8 hit enzymes were then analysed by chiral HPLC and the results are reported in Table 1.

**Scheme 1:**
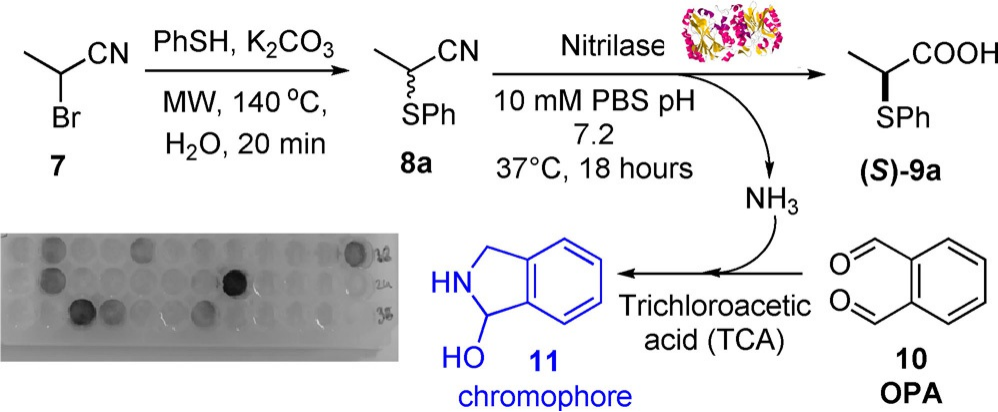
Synthesis of α‐thionitriles and colorimetric screening assay.

**Table 1:** Colorimetric screening of nitrilases.

Entry	Nitrilase	Screening plate colour^[a,b]^	Conv. [%]^[c,d]^	*ee* [%]^[c]^ **8 a**	*ee* [%]^[c]^ **9 a**
1	Nit02	weak	20	2	6
2	Nit05	weak	<1	0	ND^[e]^
3	Nit12	medium	<1	0	ND^[e]^
4	Nit14	medium	9	2	4
5	Nit20	strong	89	12	5
6	Nit27	strong	36	22	95
7	Nit28	weak	<1	0	ND^[e]^
8	Nit31	weak	<1	0	ND^[e]^

[a] The assay was carried out for 18 h at 37 °C (see Supporting Information). [b] Intensity of the colour is described on the basis of personal perception as shown in Scheme 1. [c] Determined by HPLC with ChiralPak^©^ IG column. [d] The conversion of the nitrile into the acid, determined by HPLC, is reported. [e] Not determined.

Surprisingly, several of the nitrilases which had been tipped for activity based on the assay plate demonstrated no conversion of **8 a** into the carboxylic acid **9 a** (Table 1, entries 2,3 and 7–8). This may be due to the sensitivity of the assay which is able to detect ammonia and lead to a colour change even in the presence of little enzymatic activity (<1 % conversion). On the other hand, Nit27 afforded **9 a** with 36 % conversion and an excellent 95 % ee (entry 6), while the starting nitrile was recovered with 22 % *ee*. Higher conversion (89 %) was observed with Nit20 (entry 5) but both the nitrile **8 a** and the acid **9 a** were recovered with negligible selectivity.

The absolute configuration of the acid was assigned as ***(S)*** **‐9 a** by comparison of its alpha value with that described in the literature.^16^ Due to the promisingly high *ee*, Nit27 was selected for further optimization of the reaction conditions. The effects of temperature and pH on the biocatalytic transformation were then investigated (Table 2). When the biotransformation was carried out for a longer time (24 h) an increase in the conversion and the *ee* was observed. Similar data were obtained at 25 °C, 30 °C or 37 °C (entries 2–4), while a drop in the conversion was observed at 4 °C (entry 1), likely due to a loss in enzyme activity at this temperature. On the other hand, the pH of the buffer solution had a more incisive effect on the biocatalytic hydrolysis. In fact, at pH 8 the acid ***(S)*** **‐9 a** was obtained with 68 % conversion (entry 8), clearly indicating that a DKR reaction was occurring. The rate of substrate racemization of **8 a** is faster at higher pH due to a higher proportion of the generated ammonia being present in the non‐protonated form rather than as an ammonium ion. The increased nucleophilicity of the non‐protonated NH_3_ may lead to a greater proton exchange for the α‐thionitrile substrate favouring, in turn, the formation of ***(S)*** **‐9 a** with conv. >50 %, as shown in Scheme 2 a. When the reaction was carried out at a higher pH (pH 8.5), a decrease in the conversion was initially observed, likely due to a reduced activity of the enzyme at this pH. However, when the reaction was left stirring for a longer time (7 days, Table 2, entry 9), excellent (75 %) conversion and 94 % *ee* of ***(S)*** **‐9 a** were obtained. Interestingly, under these conditions, the unreacted nitrile **8 a** was recovered with 60 % *ee*, while, when the reaction was left for 13 days, **8 a** was obtained with only 8 % *ee* (Table 2, entry 10). It is plausible that the maximum conversion of the biotransformation at pH 8.5 is reached in 7 days due to the loss of enzyme activity after this time in the presence of ammonia. Due to the basic environment, the unreacted nitrile **8 a** racemises affording, after 13 days, **8 a** with 8 % *ee*, thus indirectly confirming the proposed mechanism for the DKR reaction. Noteworthy, no racemization of the acid **9 a** occurs after 13 days. Finally, **8 a** was treated at pH 7.2 for 7 days to investigate if longer reaction times at lower pH could also lead to improved conversions and *ee*. Under these conditions, an excellent conversion of 90 % was observed but the *ee* of **9 a** slightly dropped down to 81 % (entry 11).^17^ It is likely that after some hours at pH 7.2 the Nit27 begins to also hydrolyse the **(*R*)‐8 a** enantiomer, thus resulting in a lower *ee* of **9 a**. Increasing the loading of the biocatalysts proved detrimental leading to **9 a** with poor (36 %) *ee* (Table 2, entry 13). On the other hand, the lower conversions observed at pH 8.5 after 7 days must be ascribable to the reduced activity of the enzyme under these conditions, which counterbalances the racemization process. Even if at pH 7.2 the amount of non‐protonated NH_3_ is low, it appears to be sufficient to catalyse the racemization of nitrile **8 a**, in agreement with previous literature data.^9^ In order to confirm our hypothesis on the role of ammonia in the racemization of **8 a**, two additional experiments were carried out (Scheme 2 b). The enantioenriched nitrile **8 a** (*ee*=72 %, Table 2, entry 12) was suspended in a buffer solution (pH 7.2) and treated with 1 equiv of aqueous ammonia. After 48 h, the full racemization of **8 a** was observed. On the contrary, when the enantioenriched **8 a** was left stirring for 48 h in buffer solution (pH 7.2) without any base, no racemization occurred and only a small decrease in the *ee* was observed.^18^ These experiments show that, even if ammonia at pH 7.2 mainly exists in its protonated form, the amount of free base is sufficient to favour the racemization of the α‐thionitrile substrates, thus supporting the hypothesised mechanism of the biotransformation. From previous experiments, it was clear that while the conversions of the biocatalytic transformation increase at lower pH, the best conv./*ee* ratio were obtained at higher pH.

**Table 2:** Optimization of the DKR of nitrile **8 a** with Nit27.

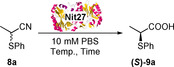						
Entry	*T* [°C]	pH	*t*	Conv. [%]^[a,b]^	*ee* [%]^[c]^ **8 a**	*ee* [%]^[c]^ **9 a**
1	4	7.2	24 h	13	9	97
2	25	7.2	24 h	41	44	99
3	30	7.2	24 h	48	56	99
4	37	7.2	24 h	46	39	99
5	37	6.5	24 h	7	4	99
6	37	7.0	24 h	11	6	93
7	37	7.5	24 h	35	23	95
8	37	8.0	24 h	68	70	93
9	37	8.5	7 d	75^[f]^	60	94
10	37	8.5	13 d	75	8	92
11	37	7.2	7 d	90^[g]^	90	81
12^[d]^	37	7.2	7 d	82	72	87
13^[e]^	37	7.2	7 d	96	77	36

[a] All the reactions were carried out on 0.012 mmol of **8 a** and 1 mg of nitrilase (Cell Free Extract, CFE). [b] Conversion of the nitrile into the acid is reported. [c] Determined by HPLC with ChiralPak^©^ IG column. [d] The reaction was carried out with 0.018 mmol of **8 a** and 1 mg of nitrilase (CFE). [e] The reaction was carried out with 0.012 mmol of **8 a** and 7 mg of nitrilase (CFE). [f] **9 a** was obtained in 67 % isolated yield. [g] **9 a** was obtained in 72 % isolated yield.

**Scheme 2:**
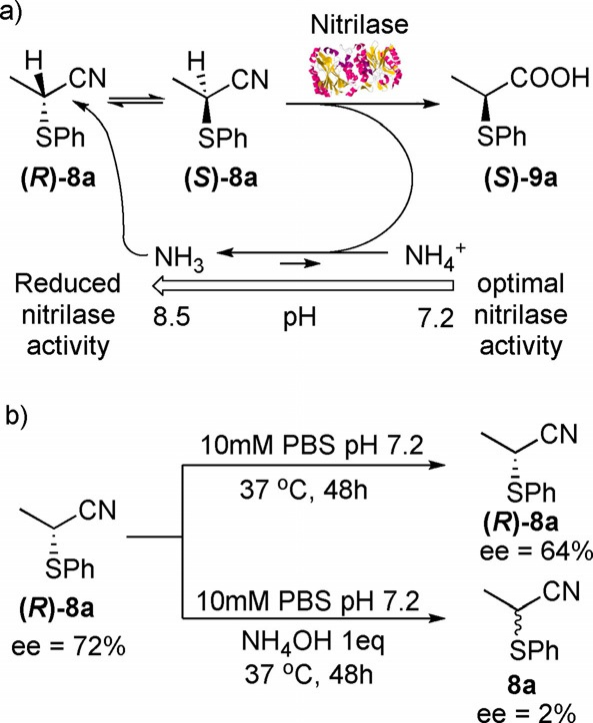
Plausible mechanism for the DKR of nitrile **8 a** and racemization experiments.

The scope of the biocatalysed hydrolysis of α‐thionitriles **8 a**–**m** with nitrilase Nit27 was thus investigated under different pH conditions. The thionitrile substrates **8 a**–**m** were synthesised from α‐bromonitrile **7** or from the appropriate cyanohydrins **10 a**–**d** as shown in Table 3. The α‐thiopropanoic acids **9 b**–**d** (Table 3, entries 1–3) were obtained from the corresponding nitriles with high to excellent conversions (50 to 93 %) and excellent *ee* (89–99 %) regardless of the pH at which the reaction was carried out. The unreacted nitriles are generally recovered with good *ee* when the reaction was performed at pH 7.2.^17^, ^19^ The thiocarboxylic acids **9 e**,**f** were also obtained with excellent *ee* but lower conversions (Table 3, entries 4 and 5), most likely due to presence of bulkier substituents on the S‐phenyl ring that may affect their interaction with the nitrilase catalytic site, while the allyl nitrile **8 g** was fully and enantioselectively converted into **9 g** (*ee* 99 %, Table 3, entry 6). All the α‐thiopropanoic acids were obtained with absolute configuration *S*, thus confirming the selectivity of the nitrilase Nit27 for the enantiomers ***(S)*** **‐8**.^16^ When the methyl substituent on the stereocentre was replaced with bulkier groups in derivatives **8 h**–**m**, low or no conversion into the corresponding acids **9** was observed. The α‐thiobutanoic acids **9 h**,**i** (Table 3, entries 7 and 8) were obtained with excellent *ee* but poor conversions, while no conversion was observed for derivatives **9 j**–**m** bearing bulkier groups. Since the reason for the poor activity of Nit27 on nitriles **8 h**–**m** was ascribable to their steric hindrance, a second screening of the nitrilase pool from Prozomix's library was carried out. For the substrate **8 h** only the nitrilase Nit34 gave a positive response in the colorimetric assay, while 4 nitrilases (Nit2, Nit6, Nit20 and Nit34) were identified to be active for the phenylacetonitrile derivative **8 j** (Table 4). As expected, Nit27 was not highlighted in the colorimetric assay, which was consistent with the lack of conversion observed in the initial reaction scope experiments. Nit20 and Nit34 fully converted the phenylacetonitrile **8 j** into the acid **9 j** although with poor *ee* (22 % and 5 % respectively, Table 4, entry 3), while irrelevant conversion was observed with Nit02 and Nit06. While both Nit20 and 34 are active upon the α‐phenyl‐substituted thionitrile **8 j**, the lack of enantioselectivity indicates that these nitrilases are less able to differentiate the two enantiomers. Surprisingly, even if the *n*Pr and the allyl groups are similar in terms of size, the derivative **8 p** was not converted by any of the four nitrilases (Table 4, entry 4). It is plausible that the shorter length of the double bond together with the different electronic properties of the allyl and *n*Pr groups may affect the binding of **8 j** and **8 p** to the catalytic site of Nit20 and thus account for the different conversions observed. On the other hand, nitrilase Nit34 proved to be effective on derivatives **8 h** and **8 k** leading to the corresponding acids **9** with good *ee* and conversions >50 %, clearly indicating a DKR of the substrate. Compounds **9 i** and **9 l**–**o** were obtained with good‐excellent *ee* (up to 93 %) but with poor conversions and yields most likely due to steric factors which may prevent the substrates **8** to interact with the enzyme catalytic site.^20^

**Table 3:** DKR of α‐thionitriles **8 b**–**m** with Nit27.

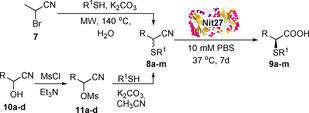						
Entry	Compound^[a]^		pH	Conv. [%]^[b]^	*ee* [%]^[b]^	Yield [%]^[c,d]^
1	**9 b**	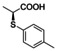	7.2 8.5	65	92	46
				50	93	39
2	**9 c**	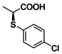	7.2 8.5	92	89	53
				93	94	57
3	**9 d**	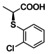	7.2 8.5	73	99	66
				52	93	41
4	**9 e**	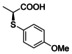	8.5	40	99	24
5	**9 f**	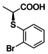	7.2 8.5	0	–	–
				6	99	ND^[e]^
6	**9 g**	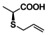	8.5	>99	99	83
7	**9 h**	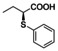	7.2	13	94	ND^[e]^
8	**9 i**	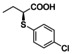	7.2	3	99	ND^[e]^
9	**9 j**	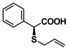	7.2 8.5	0	–	–
				0	–	–
10	**9 k**	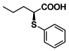	7.2 8.5	0	–	–
				0	–	–
11	**9 l**	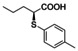	8.5	0	–	–
12	**9 m**	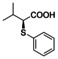	8.5	0	–	–

[a] The absolute configuration of acids **9** was assigned as *S*, by comparison of the α_D_ values with that of **9 a** and those reported in the literature.^16^ [b] Determined by HPLC with ChiralPak^©^ IG column. [c] Isolated yields are reported. [d] The low yields sometimes observed may be ascribable to the volatility of some products. [e] Not determined due to low conversion.

**Table 4:** DKR of α‐thionitriles **8 h**–**p** with Nit20 and Nit34.

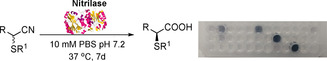						
Entry	Compound		Nitrilase	Conv. [%]^[a]^	*ee* [%]^[a]^	Yield [%]^[b]^
*1*	**9 h**	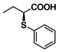	34	84	61	63
*2*	**9 i**	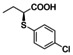	34	27	79	31
*3*	**9 j**	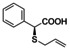	02	1	–	ND^[c]^
			06	1	–	ND^[c]^
			20	>99	22	ND^[c]^
			34	>99	5	ND^[c]^
*4*	**9 k**	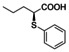	34	72	81	63
*5*	**9 l**	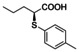	34	25	93	24
*6*	**9 m**	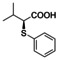	34	1	99	ND^[c]^
*7*	**9 n**	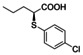	34	32	71	26
*8*	**9 o**	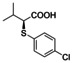	34	1	99	ND^[c]^
*9*	**9 p**	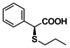	20	<1	–	ND^[c]^

[a] Determined by HPLC with ChiralPak^©^ IG column. [b] Isolated yields are reported. [c] Not determined due to low conversion.

In conclusion, nitrilase enzymes proved to be efficient biocatalysts for the enantioselective synthesis of α‐thiocarboxylic acids **9** through base‐free dynamic kinetic resolution of the corresponding racemic nitriles. Within this work two biocatalysts, namely Nit27 and Nit34, have been identified from a panel of 35 nitrilases and have shown the ability to catalyse the enantioselective synthesis of α‐thiocarboxylic acids **9** with good‐to‐excellent conversion and *ee* by DKR reaction under mild conditions.

The main advantage of the methodology relies in the exploitation of the ammonia formed during the biocatalytic hydrolysis to catalytically facilitate the in situ racemization of nitrile **8** (*R*)‐enantiomers, avoiding the need of external bases, while the nitrilase selectively hydrolyses the (*S*)‐enantiomers. To the best of our knowledge, this is the first example of base‐free DKR of α‐thionitriles by nitrilase biocatalysed hydrolysis and represents a mild, greener and straightforward means to access enantiomerically pure α‐thiocarboxylic acids, such as the flavouring compounds **4** and **5**.

## Conflict of interest

The authors declare no conflict of interest.

## Supporting information

As a service to our authors and readers, this journal provides supporting information supplied by the authors. Such materials are peer reviewed and may be re‐organized for online delivery, but are not copy‐edited or typeset. Technical support issues arising from supporting information (other than missing files) should be addressed to the authors.

Supplementary
