# Supplementary material for: Enantioselective Synthesis of α‐Thiocarboxylic Acids by Nitrilase Biocatalysed Dynamic Kinetic Resolution of α‐Thionitriles
Source: Chemistry. 2020 Jul 16;26(46):10422–6. doi: 10.1002/chem.202001108 (PMC7496879; doi:10.1002/chem.202001108)

Chemistry–A European Journal

Supporting Information

Enantioselective Synthesis of α-Thiocarboxylic Acids by Nitrilase Biocatalysed Dynamic Kinetic Resolution of α-Thionitriles

Kate Lauder,[a] Silvia Anselmi,[a] James D. Finnigan,[b] Yuyin Qi,[b] Simon J. Charnock,[b] and Daniele Castagnolo*[a]

SUPPORTING INFORMATION

**General methods**	**S2**
**Nitrilase enzymes**	**S2**
**Colourimetric Assay**	**S2**
**General procedure for the synthesis of α-thionitriles 8a-g**	**S2**
**General procedure for the synthesis of α-thionitriles 8j-k**	**S4**
**General procedure for the synthesis of α-thionitriles 8h-p**	**S5**
**General procedure for the synthesis of racemic α-thioacids 9a-p**	**S8**
**Biocatalysed synthesis of enantiopure α-thioacids 9a-p**	**S13**
**References**	**S13**
**Polarimetric Analysis of α-thioacids**	**S14**
**HPLC Analysis for α-hionitriles and acids**	**S15**
**ee (%) of the recovered nitriles**	**S16**
**Examples of HPLC spectra**	**S17**
**Racemization experiments**	**S23**
**Copies of NMR Spectra**	**S24**

General methods

Unless noted, all solvents and commercially available reagents were purchased from Sigma Aldrich and used as without further purifications. ^{1}H and ^{13}C Nuclear Magnetic Resonance (NMR) spectra were recorded using a Bruker Ascend 400 spectrometer at 298 K. Chemical shifts (δ) are reported in ppm, referenced to tetramethylsilane. Coupling constants (*J*) are reported in Hertz. Splitting patterns are abbreviated as follows: singlet (s), doublet (d), triplet (t), quartet (q), multiplet (m). TLC was performed using commercially available pre-coated plated and visualized with UV light at 254 nm. Flash column chromatography was carried out using Sigma Aldrich silica gel particle size, 40-63 µm particle size 60 Å. Biocatalytic reactions were performed by shaking the mixtures contained in 15 mL Falcon tubes using a Grant Bio™ PSU-10i Orbital Platform Shaker. A JouanB4 centrifuge with exchangeable buckets was used to centrifuge and isolate the biocatalytic products. LRMS (Low-resolution mass) and HRMS (high-resolution mass) were measured on a Thermo Q-Exactive mass spectrometer with an EI/ESI/APCI source.

Nitrilase enzymes

Nitrilase enzymes have been provided by Prozomix Ltd. Details and the primary sequence of the enzymes are available at Prozomix webpage http://www.prozomix.com.

Colourimetric Assay[1]

The colourimetric assay was used to screen a panel of 35 nitrilases against a nitrile substrate **8**. 200 µL of nitrilase CFE in pH 7.2 10 mM phosphate buffer solution (0.56 mg/mL), was added to a solution of the substrate (20 µL, 100 mM). Microcentrifuge tubes were incubated for 18 hours at 37 °C. After this time, the reaction tubes were cooled at 4 °C for 10 minutes to ensure any ammonia produced had condensed. Hits were identified by those which produced a black colour after the addition of TCA. Since only 50 µL of test solutions was required for the colourimetric assay, it was possible to analyse by HPLC those which suggested a hit enzyme. The remaining 150 µL of test solution was acidified with 1M HCl(aq), and extracted with ethyl acetate, the organic layers were concentrated *in vacuo*, resuspended in ethanol, passed through a 0.2 µM filter and analysed by HPLC.

General procedure for the synthesis of α-thionitriles 8a-g

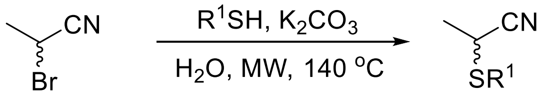

8a-g

A microwave flask containing K_2CO_3 (2 eq), 2-bromopropionitrile (0.76 mmol, 1 eq), appropriate thiol (0.84 mmol, 1.1 eq), and water (3 mL) was irradiated for 20 minutes at 140 °C in the microwave. The reaction was monitored by TLC and irradiated for a second time if starting material remained. Upon

completion of the reaction, the reaction mixture was extracted with ethyl acetate (3 mL) three times. The organic layers were collected, dried with $MgSO_4$ and concentrated *in vacuo*. The pure products **8a-g** were obtained following column purification on silica gel (eluent hexane/EtOAc 9:1).

2-(Phenylthio)propanenitrile 8a

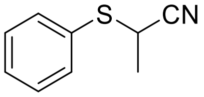

Yield: 79%. ^{1}**H NMR** (400MHz, $CDCl_3$) δ 7.66-7.59 (m, 2H), 7.46-7.37 (m, 3H), 3.81 (q, *J*=7.2 Hz, 1 H), 1.62 (d, *J*=7.2 Hz, 3H) ppm. ^{13}**C NMR** (101MHz, $CDCl_3$) δ 134.7, 130.5, 129.6, 129.4, 119.8, 31.4, 18.7 ppm. **HRMS** (APCI) m/z calcd. for $C_9H_{10}NS^+$ $[M+H]^+$ 164.0528; found 164.0528. **R*f*** 0.59 (Hexane/EtOAc 9:1).

2-(*p*-Tolylthio)propanenitrile 8b

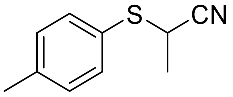

Yield: 66%. ^{1}**H NMR** (400MHz, $CDCl_3$) δ 7.48-7.41 (m, *J*=8.0 Hz, 2 H), 7.17-7.09 (m, *J*=8.1 Hz, 2H), 3.67 (q, *J*=7.2 Hz, 1H), 2.30 (s, 3H), 1.51 (d, *J*=7.2 Hz, 3H) ppm. ^{13}**C NMR** (101 MHz, $CDCl_3$) δ 140.2, 135.2, 130.2, 126.8, 120.0, 31.7, 21.3, 18.7 ppm. **HRMS** (APCI) m/z calcd. for $C_{10}H_{12}NS^+$ $[M+H]^+$ 178.0685; found 178.0685. **R*f*** 0.33 (Hexane/EtOAc 95:5).

2-((4-Chlorophenyl)thio)propanenitrile 8c

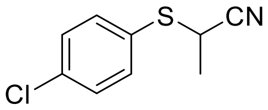

Yield: 59%. ^{1}**H NMR** (400MHz, $CDCl_3$) δ 7.51-7.42 (m, 2H), 7.34-7.24 (m, 2H), 3.70 (q, *J*=7.2 Hz, 1H), 1.51 (d, *J*=7.2 Hz, 3H) ppm. ^{13}**C NMR** (101MHz, $CDCl_3$) δ 136.2, 136.2, 129.7, 128.8, 119.6, 31.5, 18.7 ppm. **HRMS** (APCI) m/z calcd. for $C_9H_9ClNS^+$ $[M+H]^+$ 198.0139; found 198.0138. **R*f*** 0.22 (Hexane/EtOAc 9:1).

2-((2-Chlorophenyl)thio)propanenitrile 8d

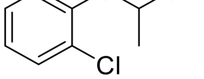

Yield: 54%. ^{1}**H NMR** (400MHz, $CDCl_3$) δ 7.73-7.66 (m, 1H), 7.50 (dd, *J*=1.9, 7.4 Hz, 1H), 7.39-7.29 (m, 2H), 4.02 (q, *J*=7.3 Hz, 1H), 1.69 (d, J=7.2 Hz, 3H) ppm. ^{13}**C NMR** (101MHz, $CDCl_3$) δ 137.9, 135.6, 130.6, 130.3, 130.3, 127.8, 119.5, 30.0, 18.2 ppm. **HRMS** (APCI) m/z calcd. for $C_9H_9ClNS^-$ $[M-H]^-$ 195.9993; found 195.9808. **R*f*** 0.21 (Hexane/EtOAc 9:1).

2-((4-Methoxyphenyl)thio)propanenitrile 8e

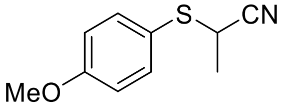

Yield: 96%. **^{1}H NMR** (400MHz, $CDCl_3$) δ 7.63-7.56 (m, 2H), 6.97-6.91 (m, 2H), 3.85 (s, 3H), 3.71 (q, *J*=7.2 Hz, 1H), 1.58 (d, *J*=6.2 Hz, 3H) ppm. **^{13}C NMR** (101MHz, $CDCl_3$) δ 161.2, 137.5, 120.6, 120.0, 115.0, 55.4, 32.1, 18.6 ppm. **HRMS** (APCI) m/z calcd. for $C_{10}H_{12}NOS^+$ $[M+H]^+$ 194.0634; found 194.0635. **R*f*** 0.18 (Hexane/EtOAc 9:1).

2-((2-Bromophenyl)thio)propanenitrile 8f

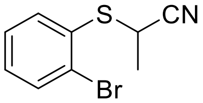

Yield: 34%. **^{1}H NMR** (400MHz, $CDCl_3$) δ 7.69 (dt, *J*=1.4, 7.5 Hz, 1H), 7.38 (dt, *J*=1.4, 7.6 Hz, 1H), 7.28-7.23 (m, 1H), 4.03 (q, *J*=7.3 Hz, 1H), 1.70 (d, *J*=7.3 Hz, 2H) ppm. **^{13}C NMR** (101MHz, $CDCl_3$) δ 135.2, 133.6, 132.5, 130.6, 128.5, 119.4, 30.4, 18.1 ppm. **HRMS** (APCI) m/z calcd. for $C_9H_9BrNS^+$ $[M+H]^+$ 241.9634 and 243.9613 ; found 241.9632 and 243.9613. **R*f*** 0.28 (Hexane/EtOAc 9:1).

2-(Allylthio)propanenitrile 8g

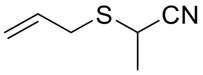

Yield: 58%. **^{1}H NMR** (400MHz, $CDCl_3$) δ 5.90-5.75 (m, 1H), 5.31-5.25 (m, 1H), 5.23 (d, *J*=10.0 Hz, 1H), 3.58 (q, *J*=7.3 Hz, 1H), 3.50-3.31 (m, 2H), 1.62 (d, *J*=7.2 Hz, 3H) ppm. **^{13}C NMR** (101 MHz, $CDCl_3$) δ 132.5, 119.7, 118.9, 35.1, 25.8, 17.9 ppm. **HRMS** (APCI) m/z calcd. for $C_6H_{10}NS^+$ $[M+H]^+$ 128.0528; found 128.0529 **R*f*** 0.58 (Hexane/EtOAc 9:1).

General procedure for the synthesis of α-thionitriles 8j and 8p

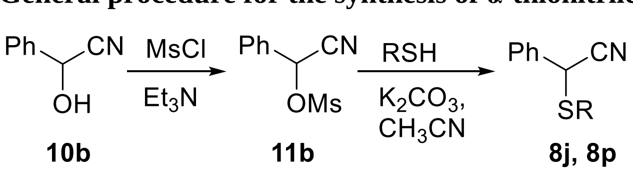

Synthesis of ***11b***. To a solution of mandelonitrile (4.51 mmol) and triethylamine (6.77 mmol) in DCM (3 mL) at 0 °C was added methanesulfonyl chloride (MsCl) (5.41 mmol). The reaction was stirred for 5 hours at 0 °C and monitored by TLC. The reaction was worked up by extraction with ethyl acetate, with the organic layers collected, dried ($MgSO_4$), and concentrated *in vacuo*. The product was filtered through a pad of silica using hexane: ethyl acetate 8:2 and used in the next step without any further purification (660 mg, 3.13 mmol, 69%).

^{1}H NMR (400MHz, $CDCl_3$) δ = 7.54-7.39 (m, 5H), 6.15 (s, 1H), 3.07 (s, 3H) ppm. **R*f*** 0.03 (Hexane/EtOAc 9:1).[2]

*Synthesis of α-thionitriles **8j** and **8p**.* The mesylated nitrile **11b** (1 mmol, 1.0 eq) was added to a flask containing K_2CO_3 (2.2 mmol, 2.2 eq), and acetonitrile (3 mL), followed by addition of the appropriate thiol (1.1 mmol, 1.1 eq). The reaction was stirred overnight at room temperature. After the starting reagents were shown to be consumed by TLC, the reaction was extracted with ethyl acetate (3 mL) and washed with brine. The organic layers were collected, dried with $MgSO_4$, and concentrated *in vacuo*. The products **8j** and **8p** were obtained after column chromatography on silica using hexane: ethyl acetate 9:1 as solvent system.

2-(Allylthio)-2-phenylacetonitrile 8j

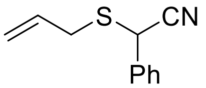

Yield: 23%. **^{1}H NMR** (400MHz, $CDCl_3$) δ 7.54-7.47 (m, 2H), 7.47-7.34 (m, 3H), 5.95-5.79 (m, 1H), 5.37-5.24 (m, 2H), 4.74 (s, 1H), 3.53 (dd, *J*=8.7, 13.9 Hz, 1H), 3.33 (dd, *J*=5.8, 13.9 Hz, 1H) ppm. **^{13}C NMR** (101MHz, $CDCl_3$) δ 208.6, 132.2, 129.2, 129.1, 128.0, 119.3, 117.7, 35.9, 35.6 ppm. **HRMS** (APCI) m/z calcd. for $C_{11}H_{12}NS$ $[M+H]^+$ 190.0685; found 190.0685. **R*f*** 0.45 (Hexane/EtOAc 9:1).

2-Phenyl-2-(propylthio)acetonitrile 8p

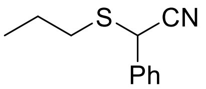

Yield: 14%. **^{1}H NMR**: (400MHz, $CDCl_3$) δ 7.60-7.48 (m, 3H), 7.44-7.36 (m, 2H), 4.81 (s, 1H), 2.81 (107, *J*=6.4, 8.0, 12.4 Hz, 2H), 1.69 (tt, *J*=7.4, 15.1 Hz, 2H), 1.03 (t, *J*=7.3 Hz, 3H). **^{13}C NMR**: (101 MHz, $CDCl_3$) δ 132.8, 129.5, 129.1, 128.9, 127.7, 127.6, 36.9, 34.0, 22.1, 13.4 ppm. **HRMS** (APCI) m/z calcd. for $C_{11}H_{14}NS^+$ $[M+H]^+$ 192.0841; found 192.0842. **R*f*** 0.36 (Hexane/EtOAc 95:5).

General procedure for the synthesis of α-thionitriles 8h-p

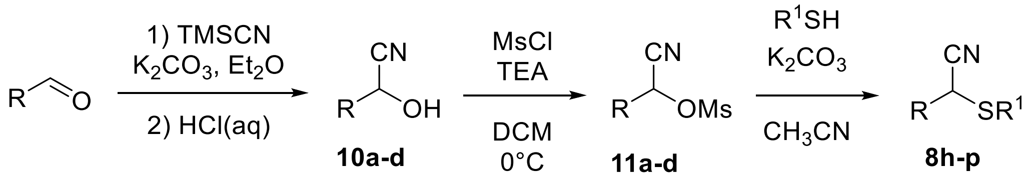

*Synthesis of cyanohydrins **10a-d**.* To a solution of the appropriate aldehyde (1 eq) in 10 mL Et_2O, TMSCN (1 eq) and K_2CO_3 (0.2 eq) were added. The reaction was stirred for 30 minutes before being quenched with saturated $NaHCO_3$ and then stirred for a further 30 minutes. The reaction was then extracted with Et_2O (5 mL). The organic layers were collected, dried with $MgSO_4$ and concentrated *in*

vacuo. To the crude OTMS-alkylnitrile 20 mL of 1 M HCl(aq) was added. The reaction mixture was stirred for 30 minutes, and then quenched with $NaHCO_3$. The product was extracted with Et_2O and washed with brine. The organic layers were collected, dried ($MgSO_4$), and concentrated *in vacuo*. The products **10a-d** were immediately used in the next step without any further purification.

*Synthesis of mesyl derivatives **11a-d***. To a solution of the 2-hydroxyalkylnitrile **10a-d** (1 eq) in Et_3N (1.5 eq) and 10 mL DCM at 0 °C, MsCl (1.2 eq) was added dropwise. The reaction was stirred for 2 hours and monitored by TLC. Upon depletion of the starting material, the reaction was quenched with water and extracted with DCM. The organic layers were dried ($MgSO_4$) and concentrated *in vacuo*. The products **11a-d** were purified using column chromatography (eluent system hexane: Et_2O 9: 1).

1-Cyanopropyl methanesulfonate 11a[2]

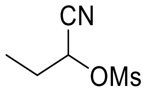

Yield: 46% yield (3 steps). Compound **11a** was filtered through a pad of silica and used in the next step without any further purification.
^{1}H NMR (400MHz, $CDCl_3$) δ 5.16 (t, *J*=6.5 Hz, 1H), 3.20 (s, 3H), 2.13-2.02 (m, 2H), 1.19-1.14 (m, 3H) ppm. **R_f** 0.45 (Hexane/EtOAc 7:3).

1-Cyanobutyl methanesulfonate 11c[3]

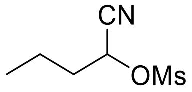

11c was obtained with 99% conversion (determined by ^{1}H NMR) and used in the next step without any further purification. **^{1}H NMR** (400MHz, $CDCl_3$) δ 5.20 (t, *J*=6.7 Hz, 1H), 3.20 (s, 3H), 2.05-1.97 (m, 2H), 1.64-1.58 (m, 2H), 1.03 (t, *J*=7.4 Hz, 3H) ppm. **R_f** 0.68 (Hexane/EtOAc 35:65).

1-Cyano-2-methylpropyl methanesulfonate 11d

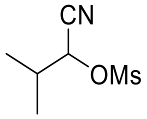

Yield: 39% yield (3 steps). Compound **11d** was filtered through a pad of silica and used in the next step without any further purification. **^{1}H NMR** (400MHz, $CDCl_3$) δ 5.01 (d, *J*=5.8 Hz, 1H), 3.20 (s, 3H), 2.28 (dd, *J*=6.7, 12.7 Hz, 1H), 1.16 (dd, *J*=6.8, 10.6 Hz, 6H) ppm. **R_f** 0.21 (Hexane/EtOAc 9:1).

*Synthesis of α-thionitriles **8h-p***. The appropriate methanesulfonate alkyl nitrile **11a-d** (0.85 mmol) was added to a flask containing K_2CO_3 (1.7 mmol) in 2 mL CH_3CN. The appropriate thiol (0.94 mmol) was

then added and the reaction mixture was allowed to stir overnight. Upon completion of the reaction, the solvent was removed by rotary evaporation, and the reaction residue then dissolved in DCM (5 mL) and washed with brine (10 mL). The organic layers were collected, dried ($MgSO_4$), and concentrated *in vacuo*. The thionitriles **8h-p** were purified by column chromatography on silica gel (eluent system hexane/EtOAc 9:1).

2-(Phenylthio)butanenitrile 8h

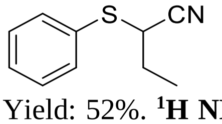

Yield: 52%. **^{1}H NMR** (400MHz, $CDCl_3$) δ 7.66-7.55 (m, 2H), 7.44-7.34 (m, 3H), 3.65 (t, *J*=7.2 Hz, 1H), 1.90 (quin, *J*=7.3 Hz, 2H), 1.19 (t, *J*=7.4 Hz, 3H) ppm. **^{13}C NMR** (101MHz, $CDCl_3$) δ 134.5, 130.8, 129.4, 119.1, 38.7, 26.1, 11.6 ppm. **HRMS** (APCI) m/z calcd. for $C_{10}H_{12}NS^+$ $[M+H]^+$ 178.0685; found 178.0685. **R*f*** 0.62 (hexane/EtOAc 9:1).

2-((4-Chlorophenyl)thio)butanenitrile 8i

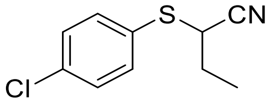

Yield: 40%. **^{1}H NMR** (400MHz, $CDCl_3$) δ 7.59-7.50 (m, *J*=8.5 Hz, 2H), 7.42-7.33 (m, *J*=8.5 Hz, 2H), 3.62 (t, *J*=7.2 Hz, 1H), 1.89 (m, *J*=7.3 Hz, 2H), 1.18 (t, *J*=7.4 Hz, 3H) ppm. **^{13}C NMR** (101MHz, $CDCl_3$) δ 136.1, 136.0, 129.7, 129.1, 118.8, 38.8, 26.1, 11.6 ppm. **HRMS** (APCI) m/z calcd. for $C_{10}H_{11}ClNS^+$ $[M+H]^+$ 212.0295; found 212.0294. **R*f*** 0.55 (Hexane/EtOAc 9:1).

2-(Phenylthio)pentanenitrile 8k

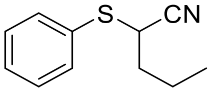

Yield: 75%. **^{1}H NMR** (400MHz, $CDCl_3$) δ 7.61-7.47 (m, 2H), 7.37-7.26 (m, 3H), 3.69-3.55 (m, 1H), 1.82-1.68 (m, 2H), 1.63-1.49 (m, 2H), 0.94-0.86 (m, 3H) ppm. **^{13}C NMR** (101MHz, $CDCl_3$) δ 134.5, 130.8, 129.4, 119.2, 36.9, 34.4, 20.4, 13.3 ppm. **HRMS** (APCI) m/z calcd. for $C_{11}H_{14}NS^+$ $[M+H]^+$ 192.0841; found 192.0842. **R*f*** 0.55 (Hexane/EtOAc 9:1).

2-(*p*-Tolylthio)pentanenitrile 8l

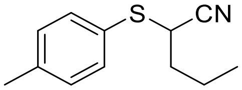

Yield: 60%. **^{1}H NMR** (400MHz, $CDCl_3$) δ 7.48-7.37 (m, *J*=8.1 Hz, 2H), 7.17-7.06 (m, *J*=7.9 Hz, 2H), 3.56 (t, *J*=7.4 Hz, 1H), 2.30 (s, 3H), 1.81-1.65 (m, 2H), 1.60-1.49 (m, 2H), 0.90 (t, *J*=7.3 Hz, 3H) ppm. **^{13}C NMR** (101MHz, $CDCl_3$) δ 140.0, 135.0, 130.2, 127.1, 119.4, 37.2, 34.4, 21.3, 20.4, 13.3 ppm.

HRMS (APCI) m/z calcd. for $C_{12}H_{16}NS^+$ $[M+H]^+$ 206.0998; found 206.0998. **R*f*** 0.54 (Hexane/EtOAc 9:1).

3-Methyl-2-(phenylthio)butanenitrile 8m

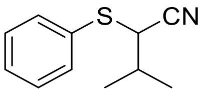

Yield: 57%. **^{1}H NMR** (400MHz, $CDCl_3$) δ 7.65-7.56 (m, 2H), 7.45-7.35 (m, 3H), 3.60 (d, *J*=6.0 Hz, 1H), 2.22-2.08 (m, 1H), 1.21 (t, *J*=6.9 Hz, 6H) ppm. **^{13}C NMR** (101MHz, $CDCl_3$) δ 133.9, 131.7, 129.5, 129.2, 118.4, 45.5, 31.3, 20.6, 19.3 ppm. **HRMS** (APCI) m/z calcd. for $C_{11}H_{14}NS^+$ $[M+H]^+$ 192.0841; found 192.0842. **R*f*** 0.51 (Hexane/EtOAc 9:1).

2-((4-Chlorophenyl)thio)pentanenitrile 8n

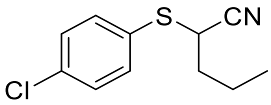

Yield: 70%. **^{1}H NMR** (400MHz, $CDCl_3$) δ 7.57-7.42 (m, 2H), 7.34-7.24 (m, 2H), 3.60 (t, *J*=7.4 Hz, 1H), 1.83-1.65 (m, 2H), 1.63-1.49 (m, 2H), 0.97-0.85 (m, 3H) ppm **^{13}C NMR** (101MHz, $CDCl_3$) δ 135.9, 135.8, 129.5, 129.0, 118.9, 36.8, 34.1, 20.2, 13.1 ppm. **HRMS** (APCI) m/z calcd. for $C_{11}H_{13}ClNS^+$ $[M+H]^+$ 226.0452; found 226.0448. **R*f*** 0.49 (Hexane/EtOAc 95:5).

2-((4-Chlorophenyl)thio)-3-methylbutanenitrile 8o

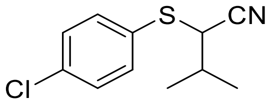

Yield: 72%. **^{1}H NMR** (400MHz, $CDCl_3$) δ 7.57-7.50 (m, 2H), 7.40-7.34 (m, 2H), 3.57 (d, *J*=6.1 Hz, 1H), 2.18-2.06 (m, 1H), 1.20 (t, *J*=6.4 Hz, 6H) ppm. **^{13}C NMR** (101MHz, $CDCl_3$) δ 135.8, 135.5, 130.0, 129.7, 118.2, 45.6, 31.3, 20.6, 19.4 ppm. **HRMS** (APCI) m/z calcd. for $C_{11}H_{14}NS^+$ $[M+H]^+$ 226.0452; found 226.0449. **R*f*** 0.58 (Hexane/EtOAc 9:1).

General procedure for the synthesis of racemic α-thioacids 9a-p

The racemic compounds **9a-p** were synthesised as reference standards for HPLC analysis and for determination of the ee excess.

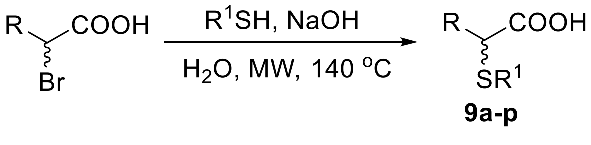

A microwave flask containing NaOH (2 eq), 2-bromoalkyl acid (1 eq) and the appropriate thiol (1.1 eq) in water (3 mL) was irradiated for 20 minutes at 140 °C in the microwave oven. The reaction was

monitored by TLC. Upon completion of the reaction, the reaction mixture was acidified with 1 M HCl(aq), then extracted with ethyl acetate (3 mL) three times. The organic layers were collected, dried with $MgSO_4$, and concentrated *in vacuo*. The products **9a-p** were isolated following column purification on silica (Hexane/EtOAc 9:1).

2-(Phenylthio)propanoic acid 9a

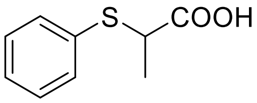

Yield 36%. **^{1}H NMR** (400MHz, $CDCl_3$) δ 7.50-7.40 (m, 2H), 7.33-7.24 (m, 3H), 3.75 (q, *J*=7.2 Hz, 1H), 1.47 (d, *J*=7.2 Hz, 3H) ppm. **^{13}C NMR** (101MHz, $CDCl_3$) δ 178.7, 133.0, 132.8, 129.0, 128.2, 45.1, 17.2 ppm. **HRMS** (APCI) m/z calcd. for $C_9H_9O_2S^+$ [M-H]$^+$ 181.0329; found 181.0332. **R_f** 0.17 (Hexane/EtOAc 7:3).

2-(*p*-Tolylthio)propanoic acid 9b

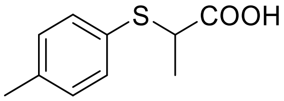

Yield: 33%. **^{1}H NMR** (400 MHz, $CDCl_3$) δ 7.54-7.50 (m, *J*=8.2 Hz, 2H), 7.24-7.19 (m, *J*=7.9 Hz, 2H), 3.75 (q, *J*=7.2 Hz, 1H), 2.38 (s, 3H), 1.59 (d, *J*=7.2 Hz, 3H) ppm. **^{13}C NMR** (101 MHz, $CDCl_3$) δ 179.0, 138.7, 133.9, 129.8, 128.8, 45.5, 21.2, 17.1 ppm. **HRMS** (APCI) m/z calcd. for $C_{10}H_{11}O_2S^+$ [M-H]$^+$ 195.0485; found 195.0488. **R_f** 0.35 (Hexane/EtOAc 9:1).

2-((4-Chlorophenyl)thio)propanoic acid 9c

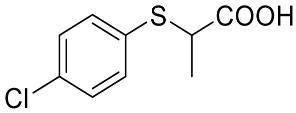

Yield: 79%. **^{1}H NMR** (400MHz, $CDCl_3$) δ 7.46-7.39 (m, 2H), 7.33-7.28 (m, 2H), 3.77 (q, *J*=7.2 Hz, 1H), 1.51 (d, *J*=7.2 Hz, 3H) ppm. **^{13}C NMR** (101MHz, $CDCl_3$) δ 178.5, 134.6, 134.5, 131.2, 129.2, 45.2, 17.1 ppm. **HRMS** (APCI) m/z calcd. for $C_9H_8ClO_2S$ [M-H]$^+$ 214.9939; found 214.9943. **R_f** 0.45 (DCM/MeOH 9:1).

2-((2-Chlorophenyl)thio)propanoic acid 9d

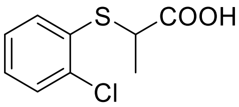

Yield: 30%. **^{1}H NMR** (400MHz, $CDCl_3$) δ 7.58-7.52 (m, 1H), 7.45 (dd, *J*=3.4, 5.9 Hz, 1H), 7.25 (dd, *J*=4.1, 5.2 Hz, 2H), 3.95 (q, *J*=7.2 Hz, 1 H), 1.59 (d, *J*=7.2 Hz, 3H) ppm. **^{13}C NMR** (101MHz, $CDCl_3$)

δ 178.1, 136.4, 133.4, 132.6, 130.0, 128.9, 127.2, 43.9, 16.9 ppm. **HRMS** (APCI) m/z calcd. for $C_9H_8ClO_2S^+$ [M-H]$^+$ 214.9939; found 214.9943. **R*f*** 0.39 (DCM/MeOH 9:1).

2-((4-Methoxyphenyl)thio)propanoic acid 9e

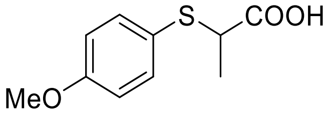

Yield: 37%. **^{1}H NMR** (400MHz, $CDCl_3$) δ 7.42-7.33 (d, *J*=8.7 Hz, 2H), 6.82-6.74 (d, *J*=8.7 Hz, 2H), 3.73 (s, 3H), 3.56 (q, *J*=7.1 Hz, 1H), 1.37 (d, *J*=7.2 Hz, 3H) ppm. **^{13}C NMR** (101MHz, $CDCl_3$) δ 178.2, 160.4, 136.5, 122.5, 114.6, 55.3, 17.0 ppm. **HRMS** (APCI) m/z calcd. for $C_{10}H_{11}O_3S^+$ [M-H]$^+$ 211.0434; found 211.0438. **R*f*** 0.54 (DCM/MeOH 9:1).

2-((2-Bromophenyl)thio)propanoic acid 9f

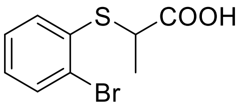

Yield: 80%. **^{1}H NMR** (400MHz, $CDCl_3$) δ 7.61 (dd, *J*=1.3, 7.9 Hz, 1H), 7.53 (dd, *J*=1.6, 7.8 Hz, 1H), 7.29 (td, *J*=1.3, 7.7 Hz, 1H), 7.15 (dt, *J*=1.6, 7.7 Hz, 1H), 3.93 (q, *J*=7.2 Hz, 1H), 1.59 (d, *J*=7.2 Hz, 3H) ppm. **^{13}C NMR** (101MHz, $CDCl_3$) δ 178.5, 134.7, 133.3, 133.0, 129.0, 127.9, 126.8, 44.2, 16.9 ppm. **HRMS** (APCI) m/z calcd. for $C_9H_8BrO_2S$ [M-H]$^+$ 260.9413 (and 258.9434); found 260.9417 (and 258.9437). **R*f*** 0.66 (DCM/MeOH 95:5).

2-(Allylthio)propanoic acid 9g

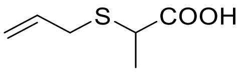

Yield: 42%. **^{1}H NMR** (400MHz, $CDCl_3$) δ 5.89-5.74 (m, 1H), 5.25-5.12 (m, 2H), 3.44-3.34 (m, 2H), 3.24 (dd, *J*=6.1, 13.8 Hz, 1H), 1.45 (d, *J*=7.2 Hz, 2 H) ppm. **^{13}C NMR** (101MHz, $CDCl_3$) δ 179.3, 133.1, 118.3, 39.4, 34.8, 16.7 ppm. **HRMS** (ESI) m/z calcd. for $C_6H_9O_2S$ [M-H]$^-$ 145.0329; found 145.0332. **R*f*** 0.29 (Hexane/Et_2O 5:5).

2-(Phenylthio)butanoic acid 9h

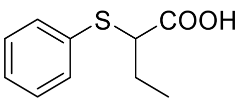

Yield: 52%. **^{1}H NMR** (400MHz, $CDCl_3$) δ 7.49 (dd, *J*=1.8, 7.5 Hz, 2H), 7.35-7.29 (m, 3H), 3.59 (t, *J*=7.4 Hz, 1H), 1.94 (qd, *J*=7.4, 14.6 Hz, 1H), 1.84 (qd, *J*=7.4, 14.3 Hz, 1H), 1.09 (t, *J*=7.4 Hz, 3H) ppm. **^{13}C NMR** (101MHz, $CDCl_3$) δ 178.7, 133.1, 132.8, 129.0, 128.0, 52.4, 24.8, 11.7 ppm. **HRMS** (APCI) m/z calcd. for $C_{10}H_{11}O_2S$ [M-H]$^+$ 195.0485 ; found 195.0487. **R*f*** 0.44 (Hexane/EtOAc 7:3).

2-((4-Chlorophenyl)thio)butanoic acid 9i

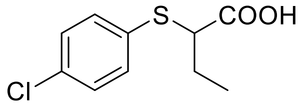

Yield: 56%. **^{1}H NMR** (400MHz, $CDCl_3$) δ 10.84 (br. s., 1H), 7.36-7.26 (m, *J*=8.4 Hz, 2H), 7.23-7.12 (m, *J*=8.5 Hz, 2H), 3.43 (t, *J*=7.4 Hz, 1H), 1.89-1.75 (m, 1H), 1.75-1.64 (m, 1H), 0.97 (t, *J*=7.4 Hz, 3H) ppm. **^{13}C NMR** (101MHz, $CDCl_3$) δ 178.3, 134.4, 134.2, 131.5, 129.1, 52.4, 24.7, 11.6 ppm. **HRMS** (APCI) m/z calcd. for $C_{10}H_{10}ClO_2S$ $[M-H]^+$ 229.0096; found 229.0097. **R*f*** 0.29 (Hexane/EtOAc 7:3).

2-(Allylthio)-2-phenylacetic acid 9j

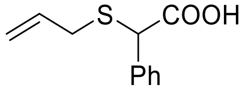

Yield: 41%. **^{1}H NMR** (400MHz, $CDCl_3$) δ 7.48-7.43 (m, 2H), 7.39-7.30 (m, 3H), 5.80 (tdd, *J*=7.2, 9.9, 17.0 Hz, 1H), 5.20-5.11 (m, 2H), 4.54 (s, 1H), 3.31-3.06 (m, 2 H) ppm. **^{13}C NMR** (101MHz, $CDCl_3$) δ 176.3, 135.2, 133.0, 128.8, 128.7, 128.4, 118.5, 50.5, 35.0 ppm. **HRMS** (APCI) m/z calcd. for $C_{11}H_{13}O_2S$ $[M+H]^+$ 209.0631; found 209.0632. **R*f*** 0.35 (Hexane/EtOAc 7:3).

2-(Phenylthio)pentanoic acid 9k

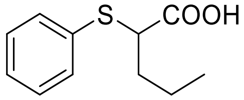

Yield: 36%. **^{1}H NMR** (400MHz, $CDCl_3$) δ 7.48 (dd, *J*=1.8, 7.5 Hz, 2H), 7.35-7.28 (m, 3H), 3.66 (t, *J*=7.5 Hz, 1H), 1.95-1.71 (m, 2H), 1.60-1.43 (m, 2H), 0.96 (t, *J*=7.3 Hz, 3H) ppm. **^{13}C NMR** (101MHz, $CDCl_3$) δ 177.2, 133.2, 132.7, 129.0, 128.1, 50.4, 33.5, 20.4, 13.6 ppm. **HRMS** (APCI) m/z calcd. for $C_{11}H_{14}O_2S$ $[M-H]^+$ 209.0642; found 209.0644. **R*f*** 0.43 (Hexane/EtOAc 7:3).

2-(*p*-Tolylthio)pentanoic acid 9l

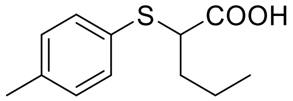

Yield: 37%. **^{1}H NMR** (400MHz, $CDCl_3$) δ 7.41-7.35 (m, *J*=8.1 Hz, 2H), 7.16-7.09 (m, *J*=7.9 Hz, 2H), 3.58 (dd, *J*=7.0, 0.9 Hz, 1H), 2.34 (s, 3H), 1.92-1.80 (m, 1H), 1.80-1.67 (m, 1H), 1.59-1.41 (m, 2H), 0.95 (t, *J*=7.3 Hz, 3H) ppm. **^{13}C NMR** (101MHz, $CDCl_3$) δ 177.2, 138.5, 133.6, 129.8, 129.2, 50.8, 33.4, 21.2, 20.4, 13.6 ppm. **HRMS** (APCI) m/z calcd. for $C_{12}H_{15}O_2S$ $[M-H]^+$ 223.0798; found 223.0801. **R*f*** 0.21 (7:3 Hexane/EtOAc).

3-Methyl-2-(phenylthio)butanoic acid 9m

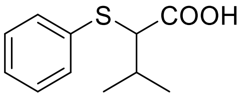

Yield: 40%. **^{1}H NMR** (400MHz, $CDCl_3$) δ 11.07 (br. s, 1H), 7.50-7.43 (m, 2H), 7.34-7.22 (m, 3H), 3.40 (d, *J*=8.6 Hz, 1H), 2.20-2.08 (m, 1H), 1.17 (d, *J*=6.8 Hz, 2H), 1.09 (d, *J*=6.6 Hz, 2H) ppm. **^{13}C NMR** (101MHz, $CDCl_3$) δ 178.5, 134.0, 132.3, 129.0, 127.8, 59.0, 30.3, 20.5, 20.1 ppm. **HRMS** (APCI) m/z calcd. for $C_{11}H_{13}O_2S$ $[M\text{-}H]^+$ 209.0642; found 209.0644. **R*f*** 0.36 (DCM/MeOH 9:1).

2-((4-Chlorophenyl)thio)pentanoic acid 9n

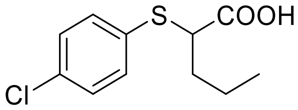

Yield: 58%. **^{1}H NMR** (400MHz, $CDCl_3$) δ 7.41 (d, *J*=8.6 Hz, 2H), 7.29 (d, *J*=8.6 Hz, 2H), 3.61 (t, *J*=7.5 Hz, 1H), 1.93-1.67 (m, 2H), 1.59-1.42 (m, 2H), 0.96 (t, *J*=7.4 Hz, 3H) ppm. **^{13}C NMR** (101MHz, $CDCl_3$) δ 177.0, 134.5, 134.2, 131.5, 129.2, 50.4, 33.3, 20.4, 13.6 ppm. **HRMS** (APCI) m/z calcd. for $C_{11}H_{12}ClO_2S$ $[M\text{-}H]^+$ 243.0252; found 243.0254. **R*f*** 0.29 (Hexane/EtOAc 7:3).

2-((4-Chlorophenyl)thio)-3-methylbutanoic acid 9o

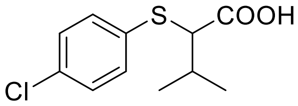

Yield: 53%. **^{1}H NMR** (400MHz, $CDCl_3$) δ 7.41 (d, *J*=8.5 Hz, 2 H), 7.28 (d, *J*=7.8 Hz, 2H), 3.38 (d, *J*=8.6 Hz, 1H), 2.17-2.08 (m, 1H), 1.18 (d, *J*=6.7 Hz, 3H), 1.10 (d, *J*=6.7 Hz, 3H) ppm. **^{13}C NMR** (101MHz, $CDCl_3$) δ 177.6, 134.1, 133.8, 132.4, 129.2, 59.1, 30.2, 20.5, 20.0 ppm. **HRMS** (APCI) m/z calcd. for $C_{11}H_{12}ClO_2S$ $[M\text{-}H]^+$ 243.0252; found 243.0254. **R*f*** 0.32 (Hexane/EtOAc 7:3).

2-Phenyl-2-(propylthio)acetic acid 9p

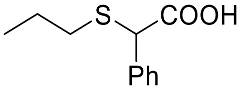

Yield 38%. **^{1}H NMR** (400MHz, $CDCl_3$) δ 7.49 (d, *J*=6.9 Hz, 2H), 7.41-7.29 (m, 3H), 4.58 (s, 1H), 2.64-2.48 (m, 2H), 1.62 (sxt, *J*=14.4 Hz, 2H), 0.97 (t, *J*=7.3 Hz, 3H) ppm. **^{13}C NMR** (101MHz, $CDCl_3$) δ 177.1, 135.5, 128.7, 128.5, 128.3, 51.9, 34.2, 22.3, 13.4 ppm. **HRMS** (ESI) m/z calcd. for $C_{11}H_{15}O_2S$ $[M+H]^+$ 211.0787 ; found 211.0525. **R*f*** 0.29 (Hexane/Et_2O 5:5) **R*f*** 0.18 (DCM/MeOH 95:5)

Biocatalysed synthesis of enantiopure α-thioacids 9a-p

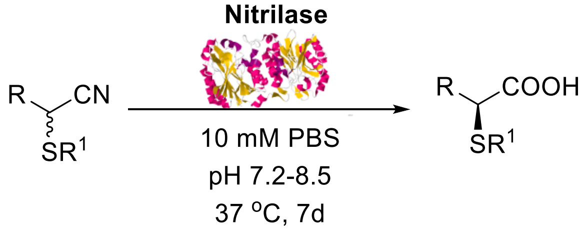

Enzymatic reactions were carried out in 1.5 mL microcentrifuge tubes, to which the nitrile substrate in ethanol (70 µL, 0.012 mmol), nitrilase (1 mg in 100 µL buffer), and 1230 µL of 10 mM phosphate buffer solution were added. The pH of the buffer was adjusted according to the data reported in the manuscript. The reactions were incubated for 7 days at 37 °C, and then acidified with 1 M HCl(aq). The reaction mixture was extracted with ethyl acetate, the organic layers collected, dried ($MgSO_4$), and concentrated *in vacuo*. A portion of the residue was dissolved in ethanol, passed through a 0.2 uM filter, and injected into HPLC to determine the conversion and ee. The α_D values were taken by basifying the crude mixture with 1 M NaOH(aq) and extracting the sample with ethyl acetate to remove the nitrile starting material. The mixture was reacidified with 1 M HCl(aq) and extracted again with ethyl acetate and the organic layers were concentrated *in vacuo*. The crude α-thioacids were purified by column chromatography, and then dissolved in an appropriate volume of chloroform to obtain a 0.1% concentration (g/100mL).

References

1. G. W. Black, N. L. Brown, J. J. B. Perry, P. D. Randall, G. Turnbull, M. Zhang, *Chem. Commun.*, **2015**, *51*, 2660–2662.
2. J. L. Marco, S. T. Ingate, C. Jaime, I. Beà, *Tetrahedron*, **2000**, *56*, 2523–2531.
3. F. Effenberger, U. Stelzer, *Chem. Ber.*, **1993**, *126*, 779-86.

Table S1. Polarimetric Analysis of α-Thioacids

Substrate	Nitrilase	Enantiomer	ee (%)	$[a]^{24}_D$
**9a**	Nit27	S	94	-9.2
**9b**	Nit27	S	93	-9.8
**9c**	Nit27	S	94	-18.7
**9d**	Nit27	S	99	-7.5
**9e**	Nit27	S	99	-10.4
**9f**	– [a]	S	-	–
**9g**	Nit27	S	99	-16.2
**9h**	Nit34	S	61	-11.8
**9i**	Nit34	S	79	–[a]
**9j**	Nit20	S	22	-4.9
**9k**	Nit34	S	81	-14.3
**9l**	Nit34	S	93	-15.1
**9m**	Nit34	S	99	– [b]
**9n**	Nit34	S	71	-8.6
**9o**	Nit34	S	99	– [b]
**9p**	– [a]	S	-	– [b]

[a] Not obtained.

[b] Yield too low for accurate α_D measurement.

[c] All α_D values were measured in $CHCl_3$, c= 0.1%, 24 °C.

Table S2. HPLC Analysis for α-Thionitriles and acids

HPLC analyses were carried out with a reverse phase Agilent series 1100 with a DAD detector set to λ = 254 nm. The column was a ChiralPak© IG (Daicel) (5 µm, 4.6 mm X 250 mm), with heptane/EtOH as an isocratic mobile phase at a flowrate of 1 ml min^{-1}.

Compounds	HPLC Column	Mobile Phase	RT Nitrile 8 / min	RT Acid 9/ min
**8-9a**	ChiralPak© IG	Heptane/EtOH 98:2	11.9 & 13.0	19.8 (S) & 21.5 (R)
**8-9b**	ChiralPak© IG	Heptane/EtOH 98:2	11.1 & 12.1	20.2 (S) & 23.5 (R)
**8-9c**	ChiralPak© IG	Heptane/EtOH 98:2	12.0 & 13.3	20.1 (S) & 22.8 (R)
**8-9d**	ChiralPak© IG	Heptane/EtOH 98:2	11.9[a]	26.1 (R) & 27.0 (S)
**8-9e**	ChiralPak© IG	Heptane/EtOH 98:2	22.7 & 24.6	39.1 (S) & 44.2 (R)
**8-9f**	ChiralPak© IG	Heptane/EtOH 98:2	12.3 & 12.5	19.4 & 21.2[b]
**8-9g**	ChiralPak© IG	Heptane/EtOH 98:2	9.7[a]	14.5 (S) & 16.9 (R)
**8-9h**	ChiralPak© IG	Heptane/EtOH 98:2	13.6 & 14.4	24.8 (S) & 25.9 (R)
**8-9i**	ChiralPak© IG	Heptane/EtOH 98:2	14.4 & 15.3	20.8 & 21.8[b]
**8-9j**	ChiralPak© IG	Heptane/EtOH 98:2	5.6[a]	14.3 (R) & 16.5 (S)
**8-9k**	ChiralPak© IG	Heptane/IPA 99:1	13.6 & 16.2	40.3 (S) & 44.7 (R)
**8-9l**	ChiralPak© IG	Heptane/EtOH 98:2	9.9 & 10.5	16.6 (S) & 18.0 (R)
**8-9m**	ChiralPak© IG	Heptane/EtOH 98:2	9.4 & 9.8	23.5[a]
**8-9n**	ChiralPak© IG	Heptane/EtOH 98:2	11.5 & 11.9	15.5 (S) & 16.9 (R)
**8-9o**	ChiralPak© IG	Heptane/EtOH 98:2	13.7 & 14.1	21.6 (R) & 22.6 (S)
**8-9p**	ChiralPak© IG	Heptane/EtOH 98:2	10.2 & 11.6	13.8 & 15.0[b]

[a] Not separated

[b] Enantiomer order of elution not determined

Table S3. ee (%) of the recovered nitriles

Cmpd	pH	Nitrilase	ee (%) recovered nitrile
**8b**	7.2	Nit27	42
	8.5		9
**8c**	7.2	Nit27	>99
	8.5		31
**8d**	7.2	Nit27	ND[a]
	8.5		ND[a]
**8e**	8.5	Nit27	8
**8f**	7.2	Nit27	-
	8.5		ND[a]
**8g**	8.5	Nit27	ND
**8h**	7.2	Nit27	5
	7.2	Nit34	37
**8i**	7.2	Nit27	1
	7.2	Nit34	4
**8j**	7.2	Nit27	-
	8.5		-
	7.2	Nit02	-
	7.2	Nit06	-
	7.2	Nit20	ND[a]
	7.2	Nit34	ND[a]
**8p**	7.2	Nit20	ND
**8k**	7.2	Nit27	-
	8.5		-
	7.2	Nit34	18
**8l**	8.5	Nit27	-
	7.2	Nit34	0
**8n**	7.2	Nit34	0
**8m**	8.5	Nit27	-
	7.2	Nit34	-[a]
**8o**	7.2	Nit34	2

[a]Not determined; the nitrile enantiomers could not be separated by HPLC

Examples of HPLC spectra

Nitrile **8a** racemic mixture

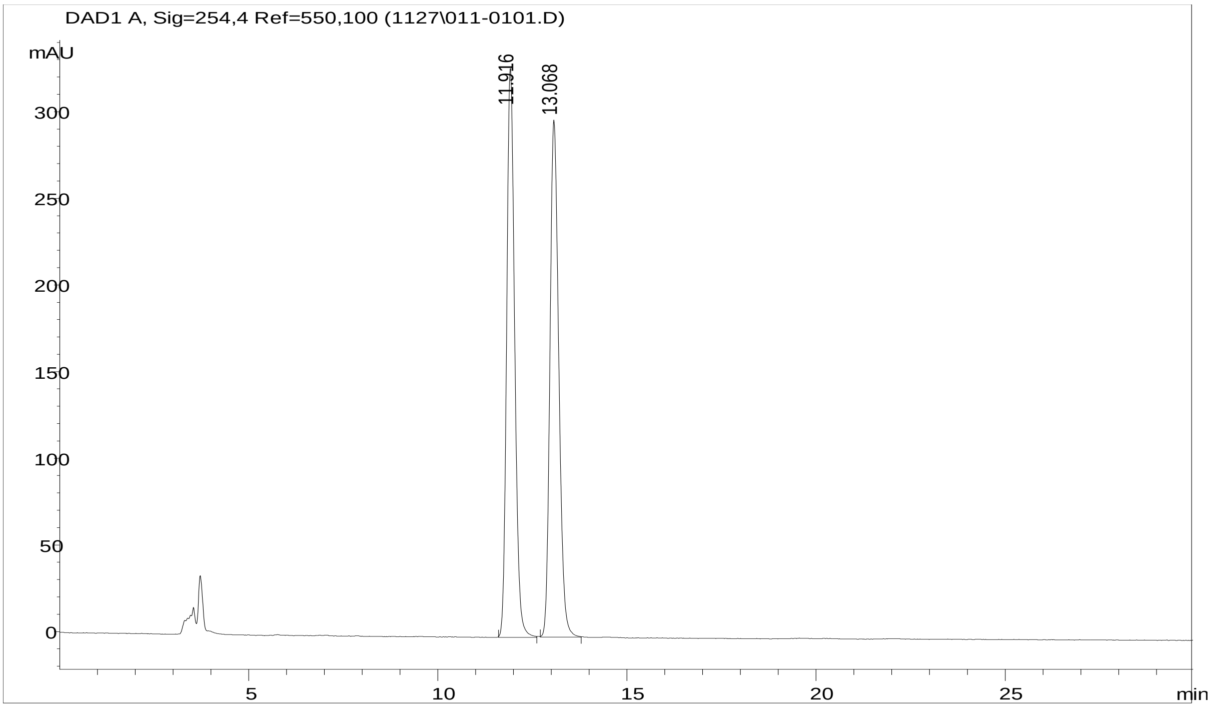

Acid **9a** racemic mixture

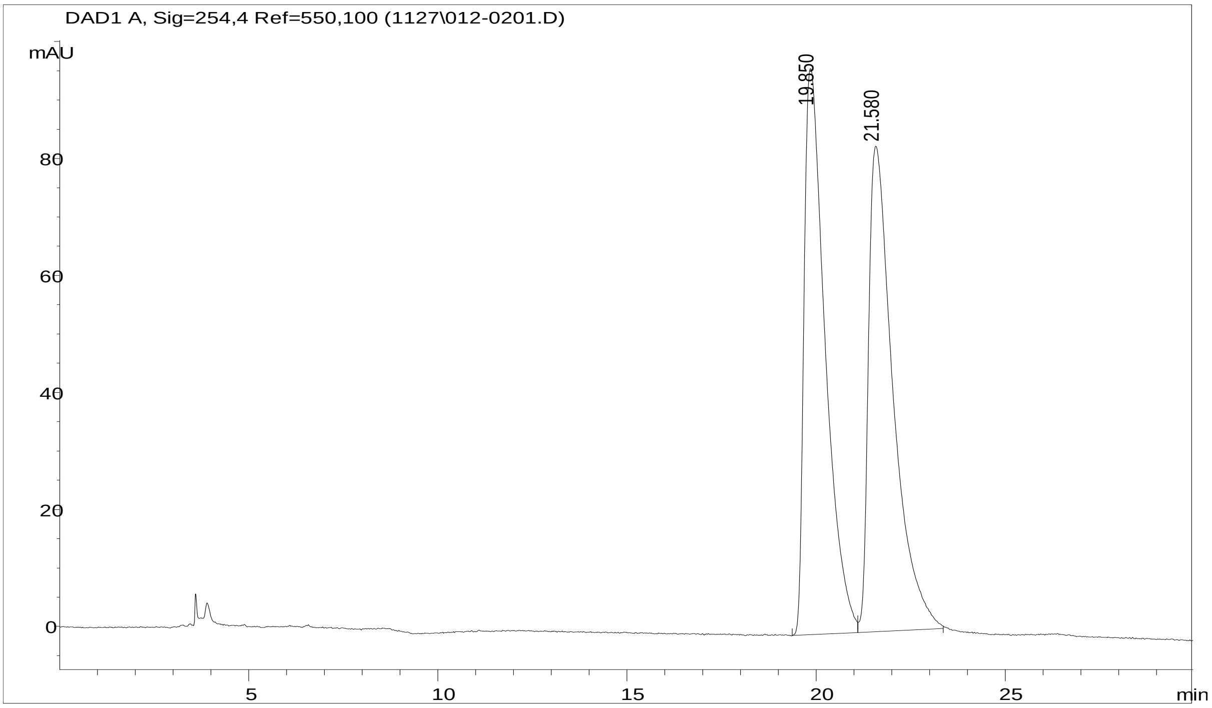

8-9a with Nit 27 at pH 7.2 after 7d

Nitrile **8a** (11.916 + 13.448) and acid **9a** (19.1916 + 21.960)

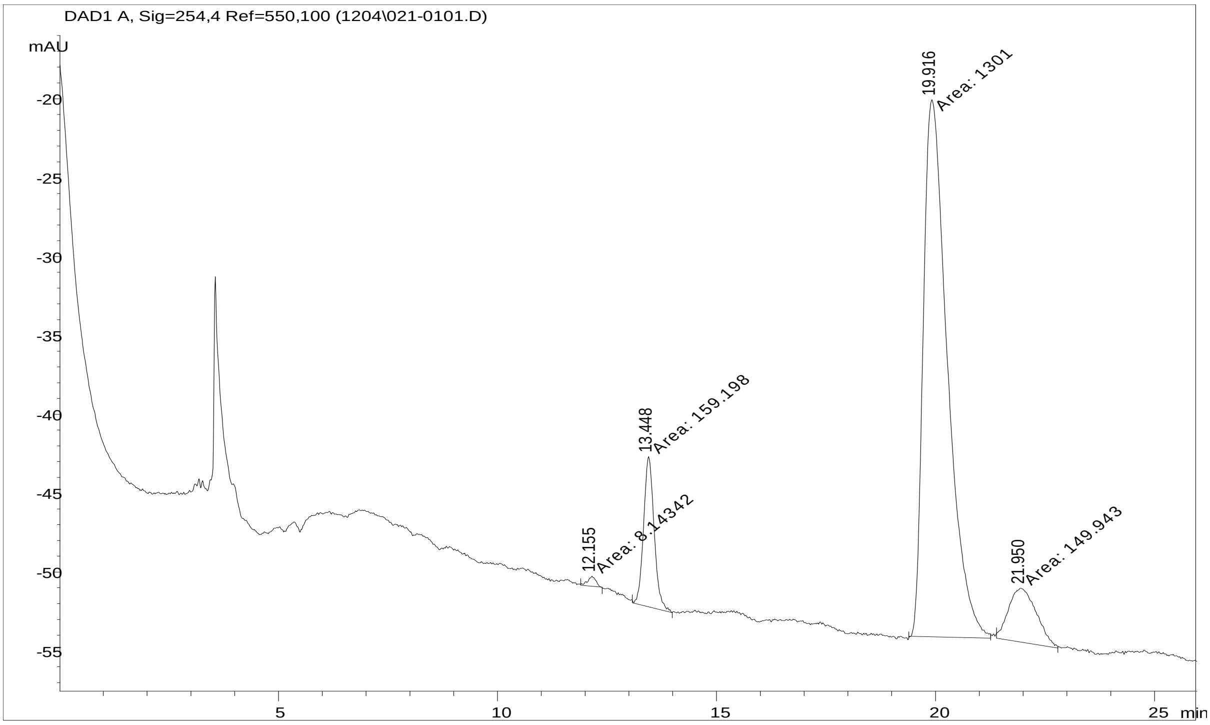

8-9b with Nit 27

Nitrile **8b** (11.077 + 12.090) and acid **9b** (20.232 + 23.538)

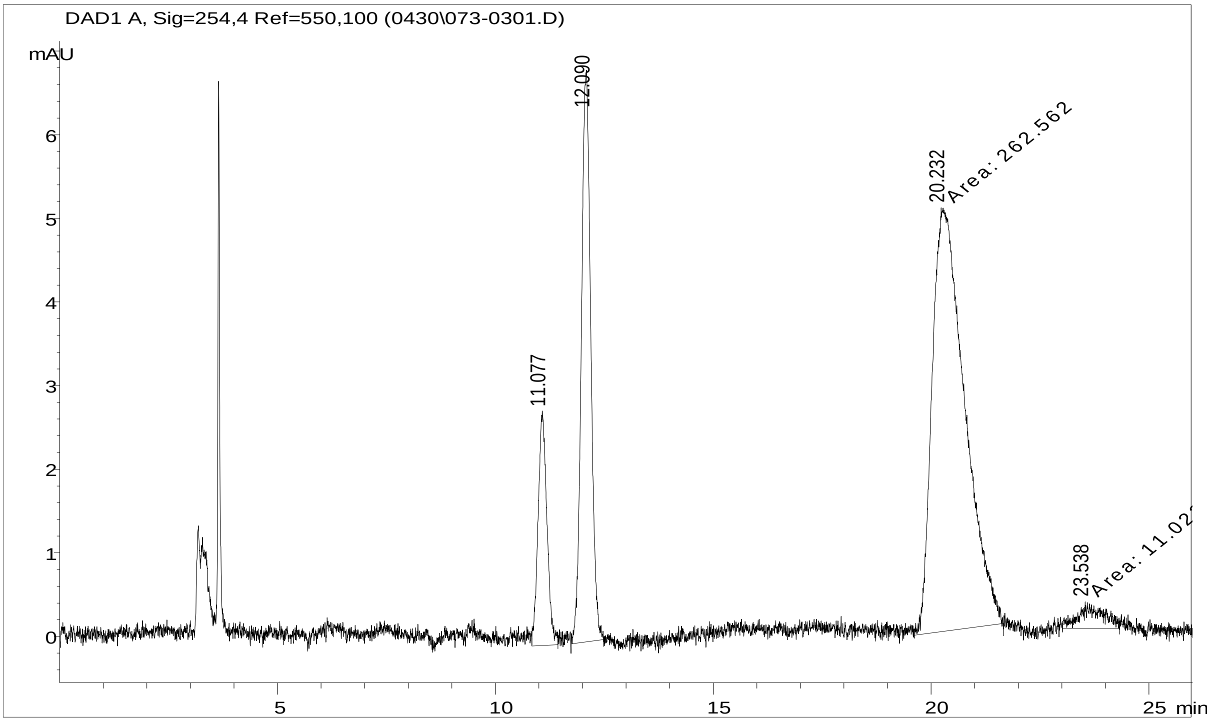

8-9c with Nit 27

Nitrile **8c** (14.323) and acid **9c** (20.148 + 22.822)

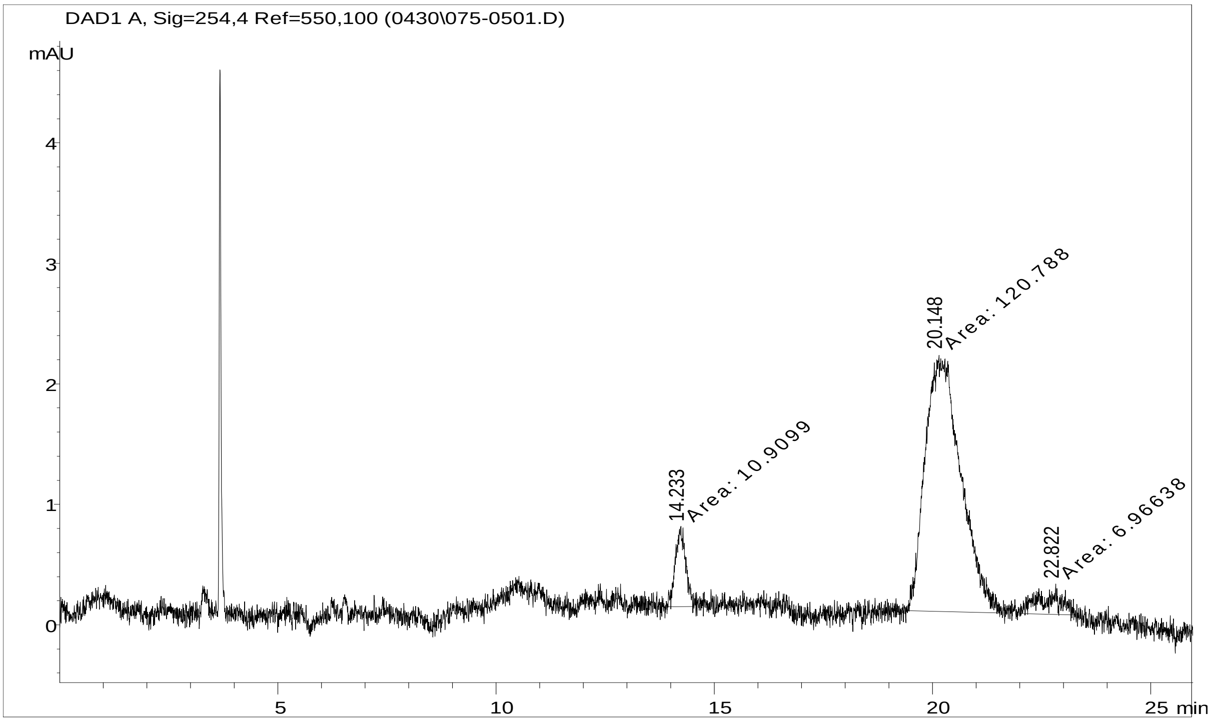

8-9h with Nit 34

Nitrile **8h** (13.688 and 14.003) and acid **9h** (24.862 and 25.927)

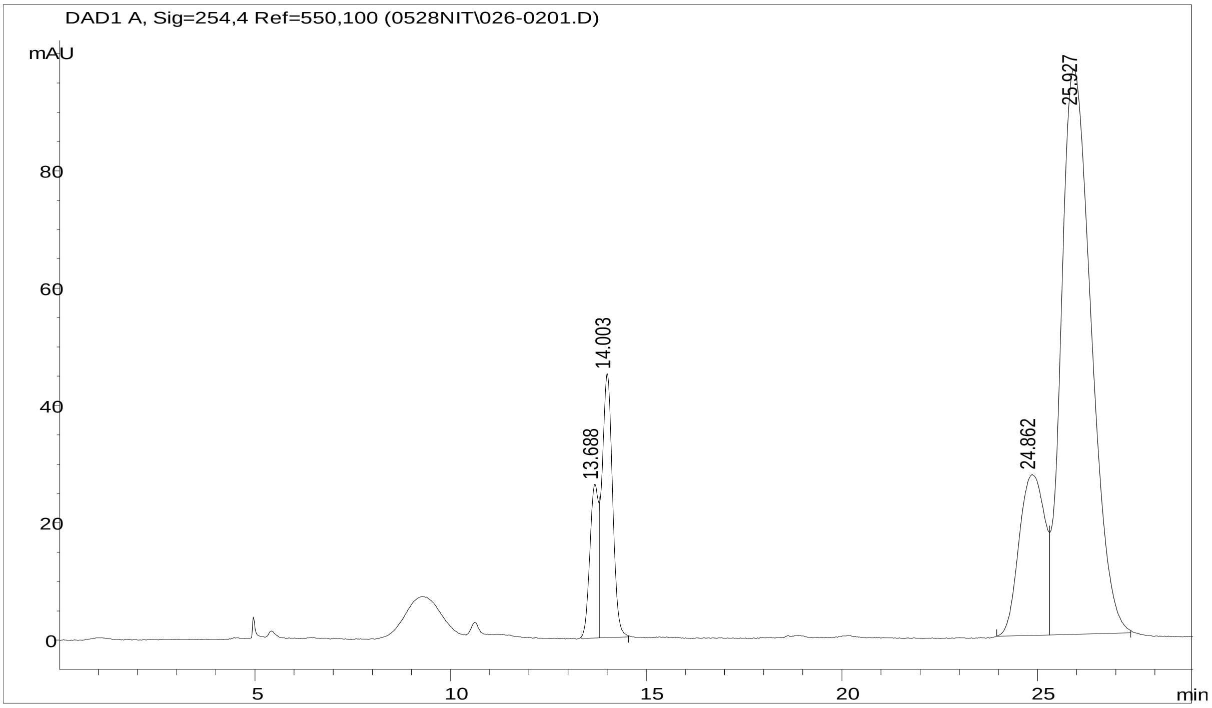

8-9i with Nit 34

Nitrile **8i** (14.453 and 15.351) and acid **9i** (20.831 and 21.818)

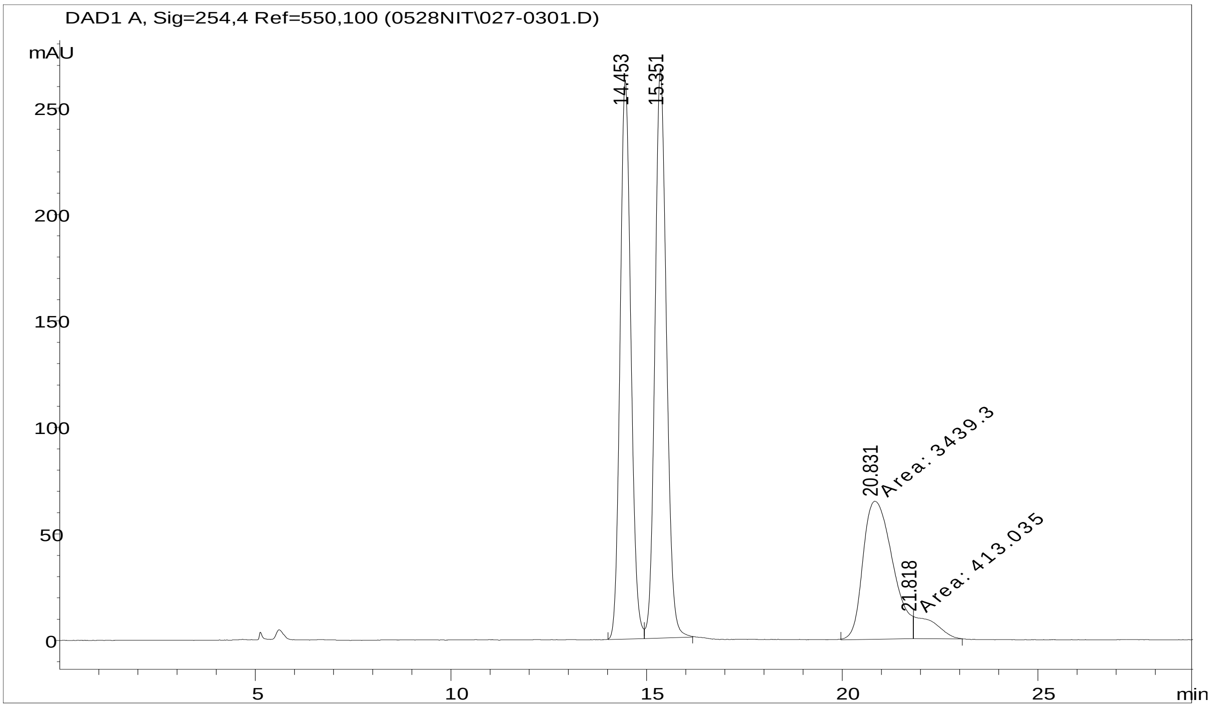

8-9l with nit34

Nitrile 8l (9.921 and 10.501) and acid 9l (16.621 and 18.022)

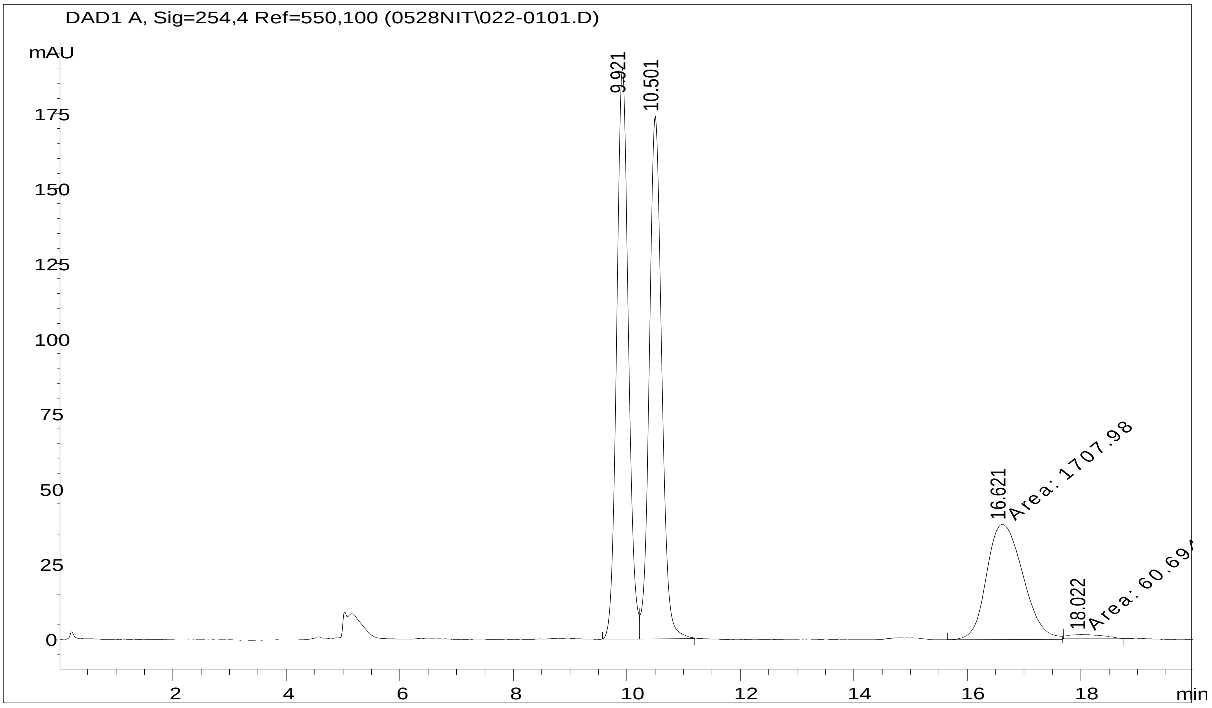

8j Racemic substrate

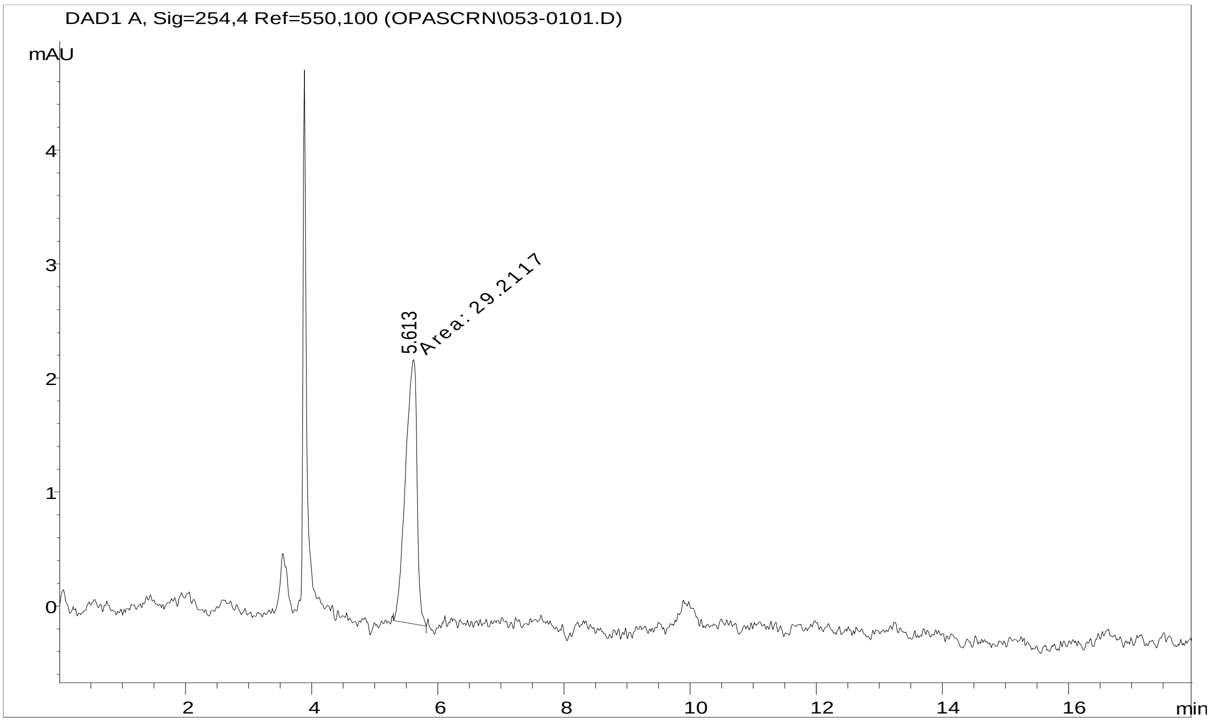

8-9j with Nit 20

8j (5.613) and **9j** (14.216 and 16.451)

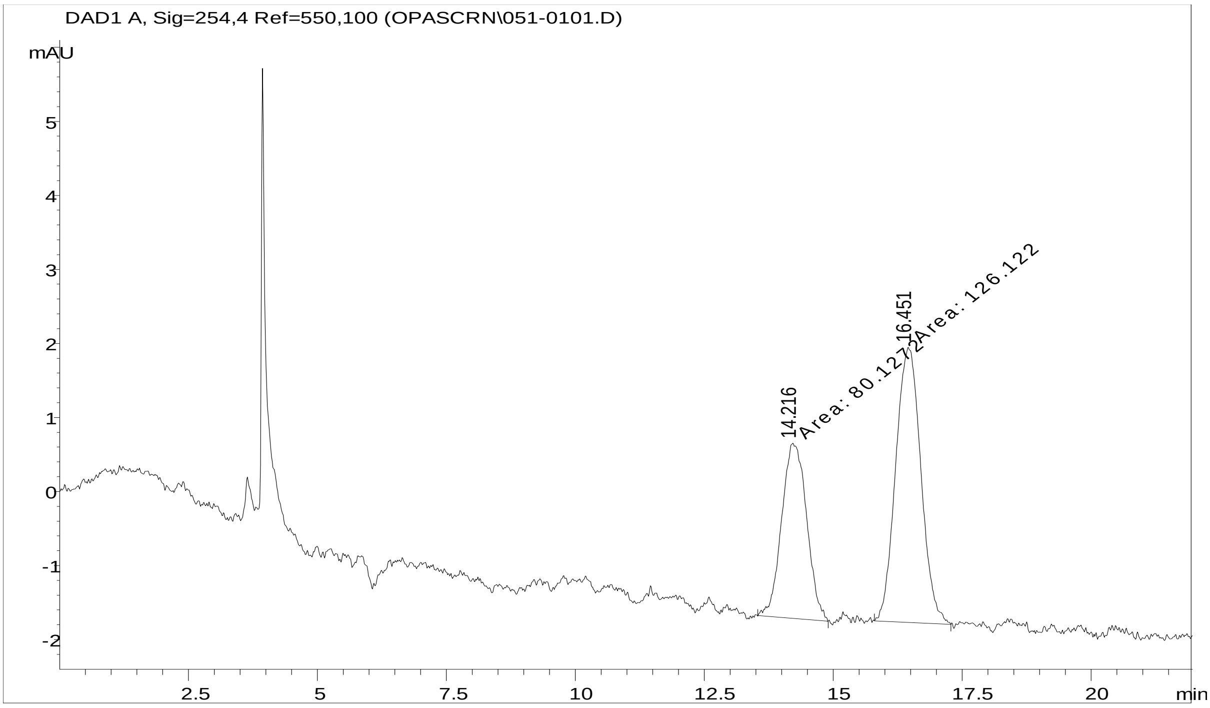

8-9j with Nit 34

8j (5.613) and **9j** (14.298 and 16.478)

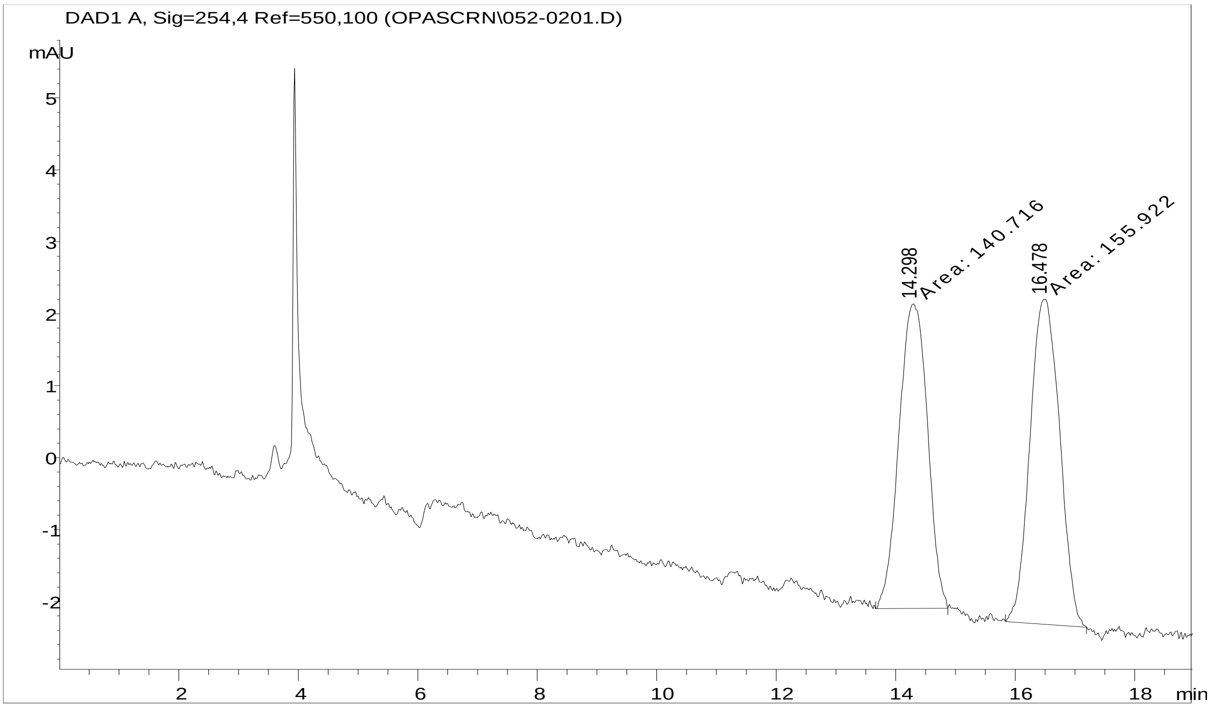

8-9k with Nit34

8k (13.554 and 16.180) and **9k** (40.272 and 44.658)

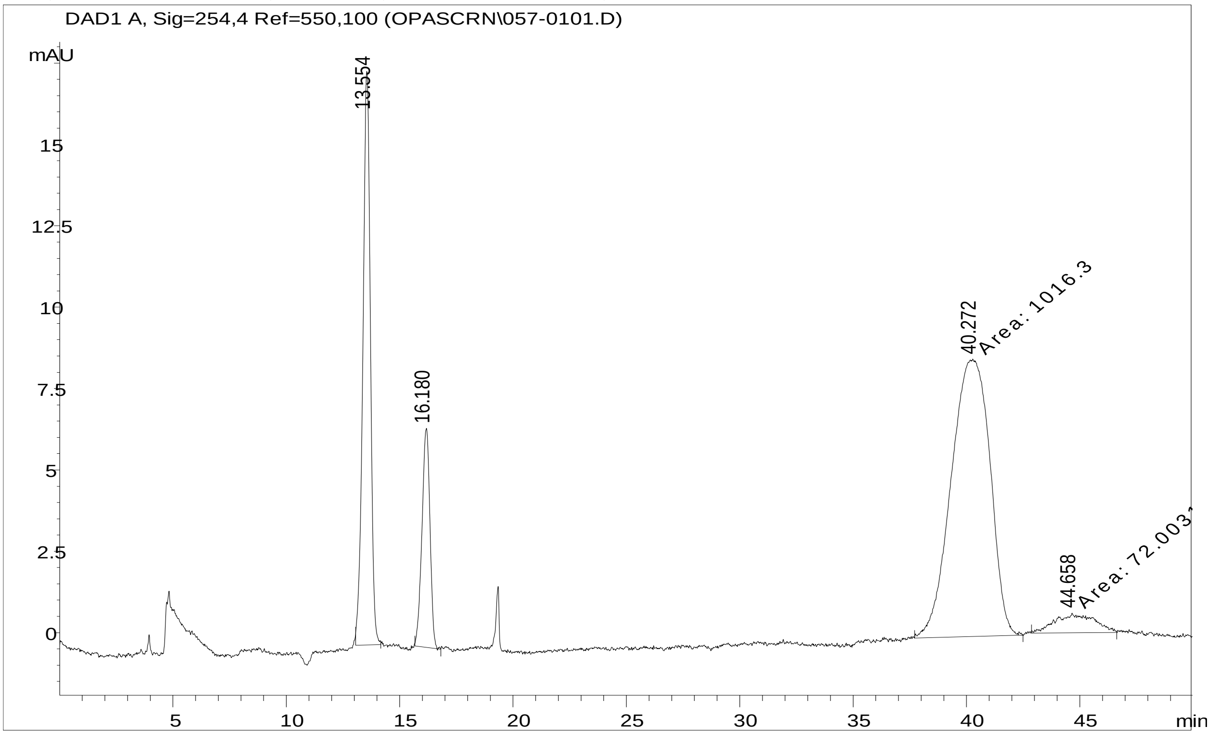

Racemization experiments

Enantioenriched nitrile **8a** after stirring in 10mM PBS (pH = 7.2) for 48h

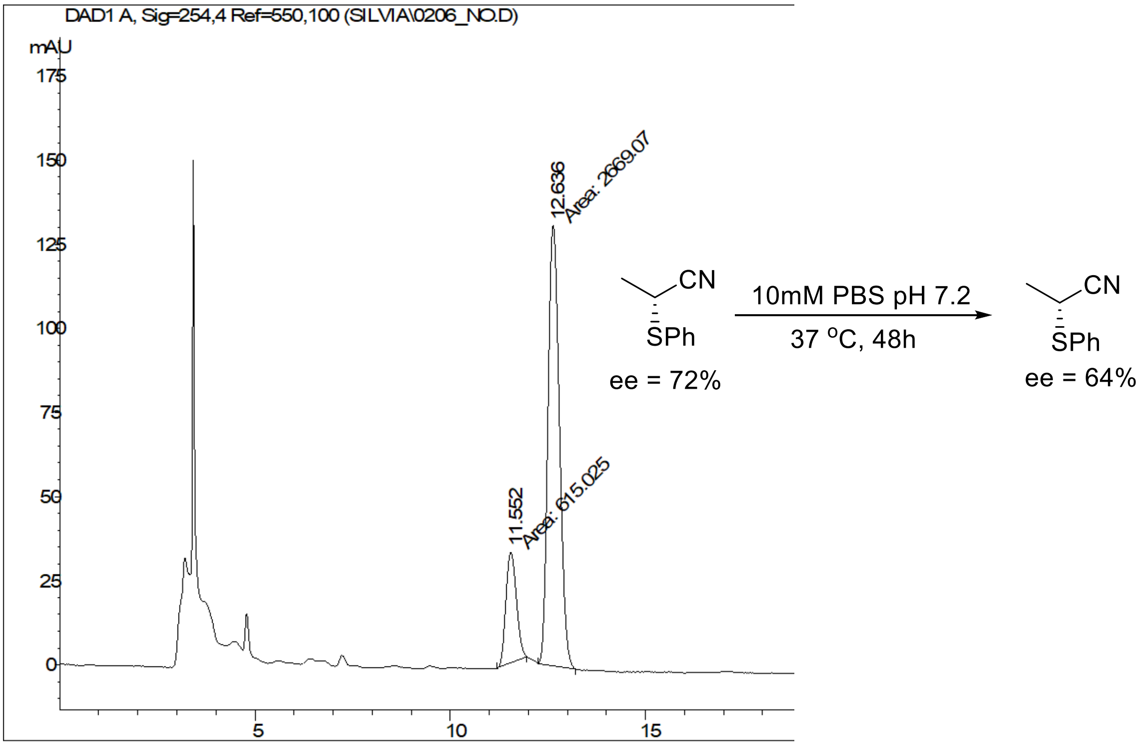

Enantioenriched nitrile **8a** after treatment with 1eq NH_4OH for 48h in10mM PBS (pH = 7.2) for 48h
Full racemization occurred

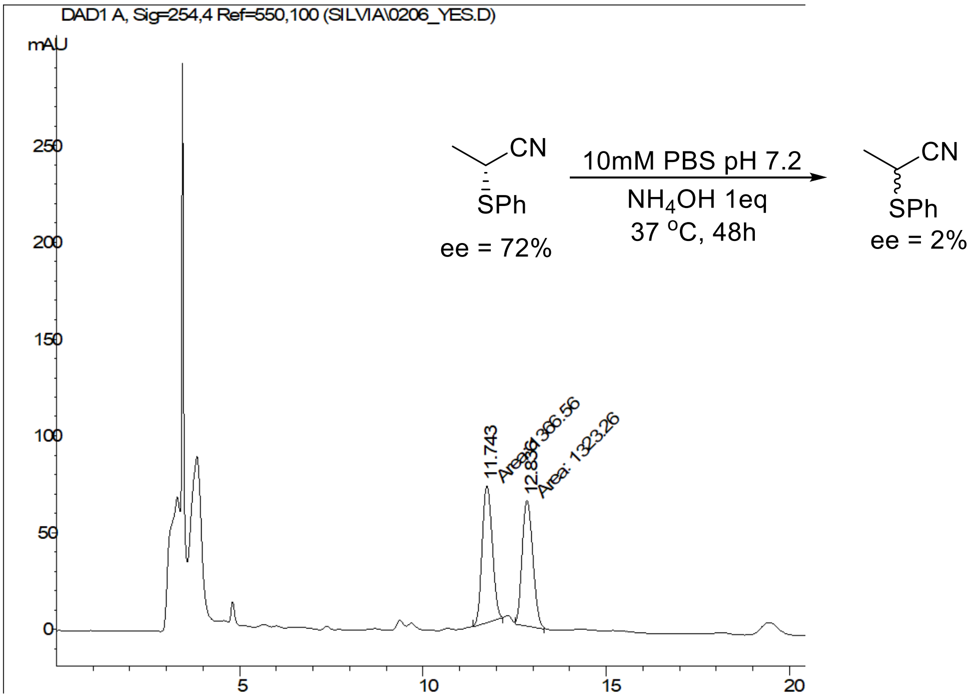

Copies of NMR Spectra

2-(Phenylthio)propanenitrile 8a

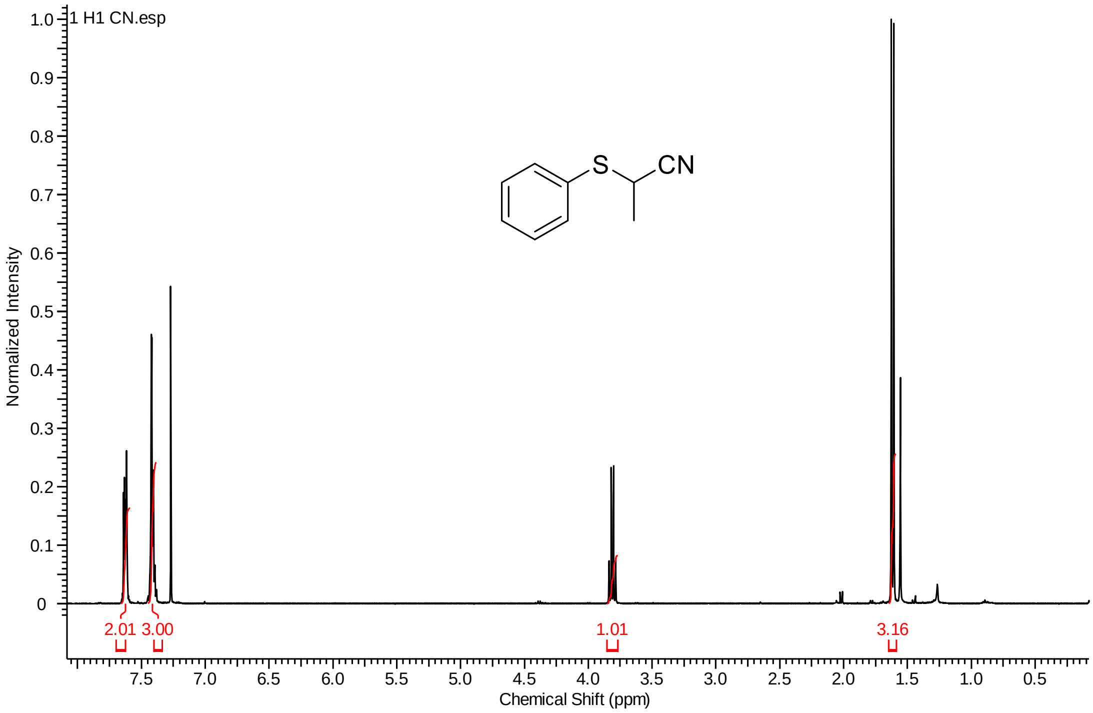

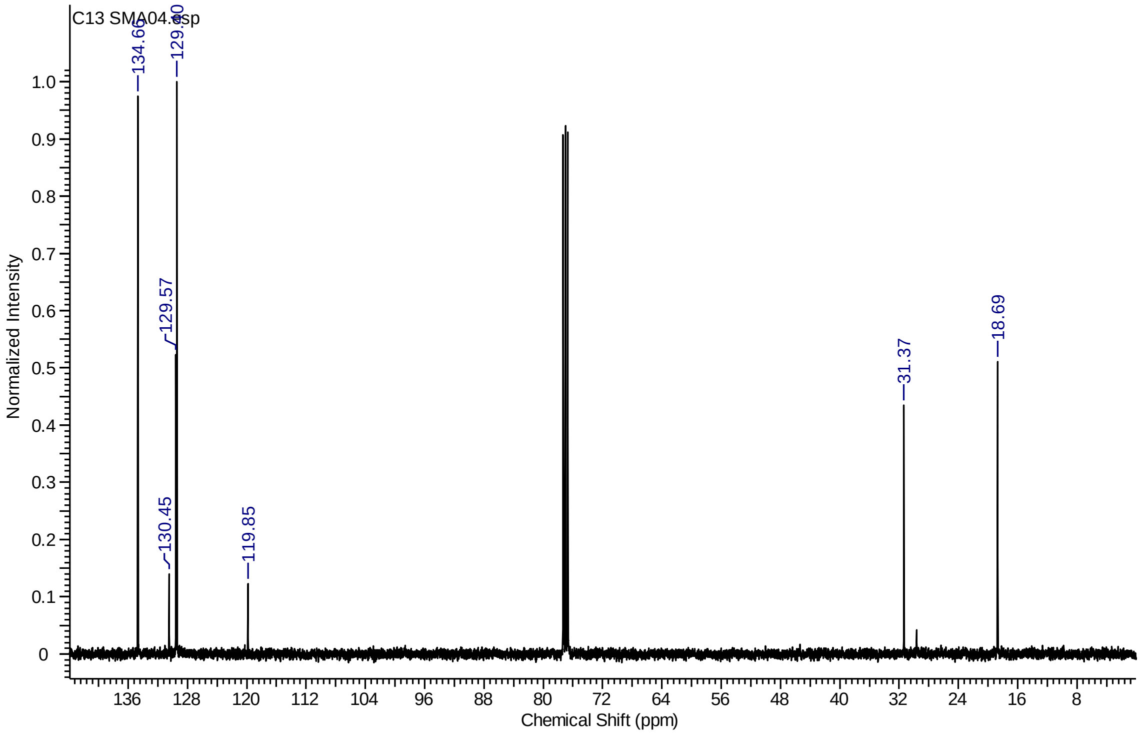

2-(*p*-Tolylthio)propanenitrile 8b

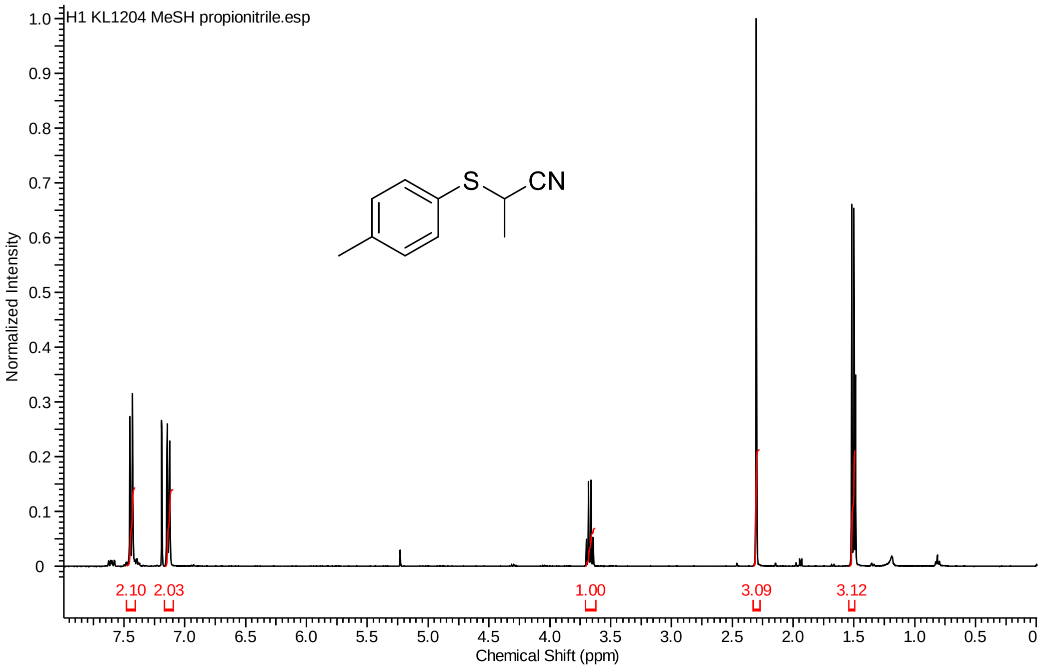

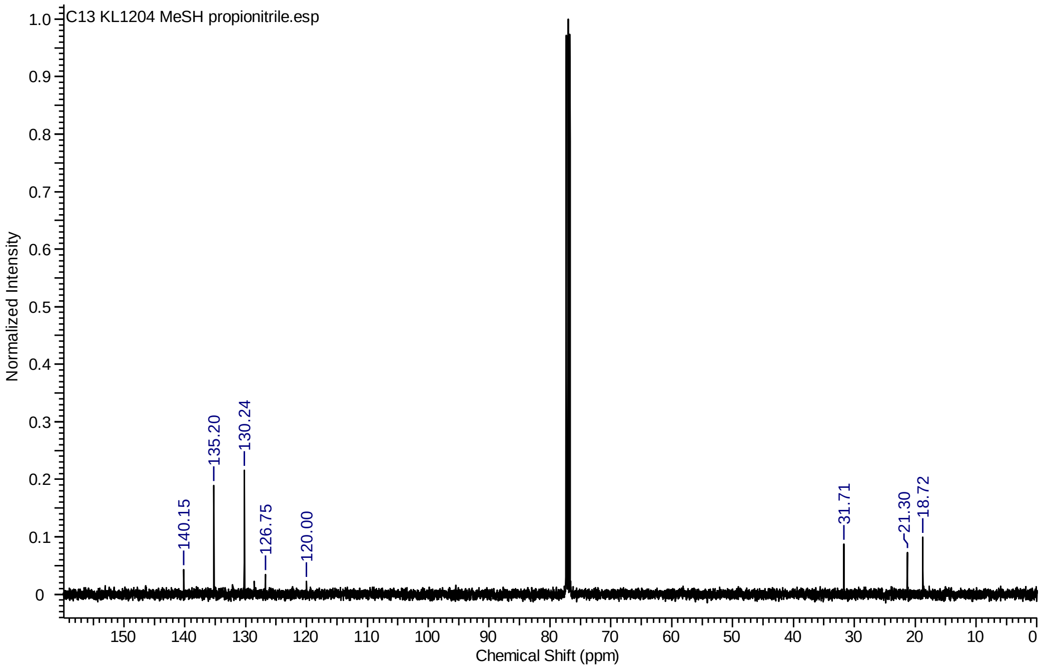

2-((4-Chlorophenyl)thio)propanenitrile 8c

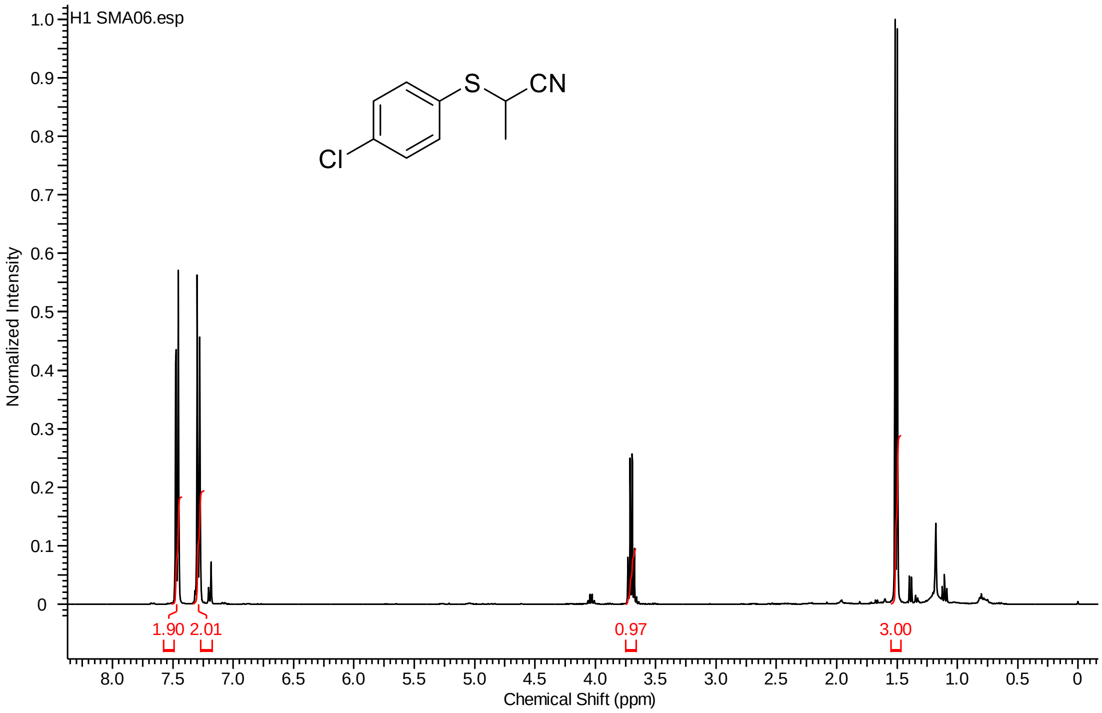

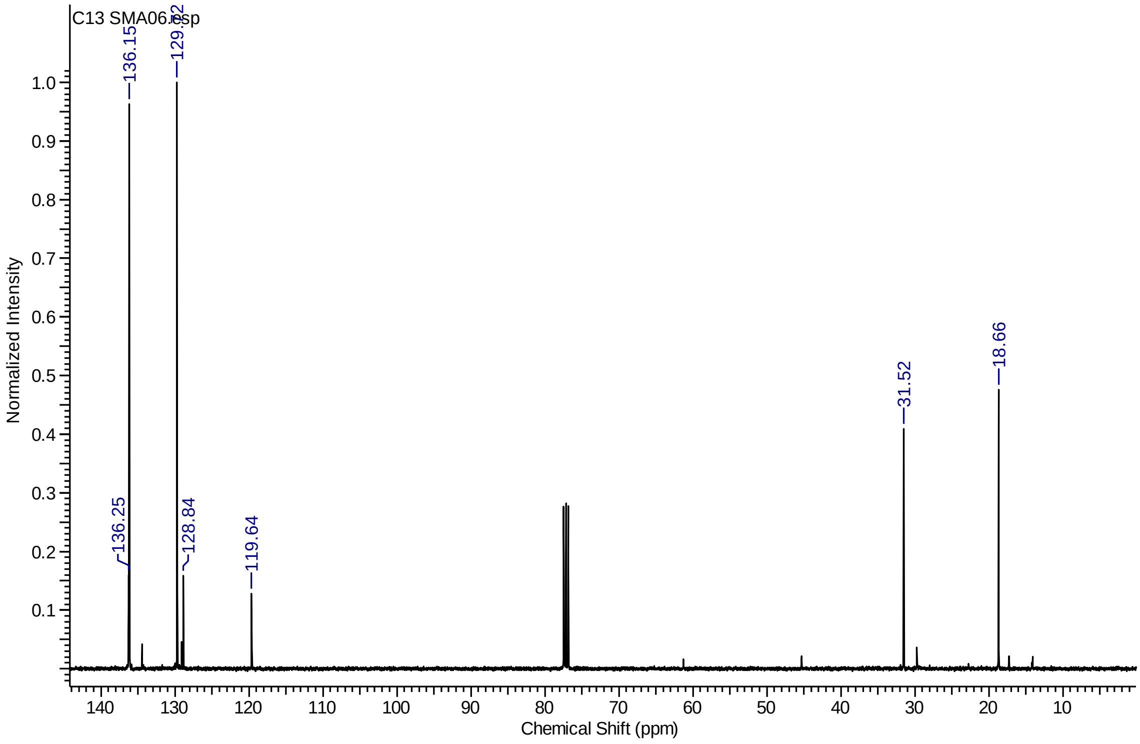

2-((2-Chlorophenyl)thio)propanenitrile 8d

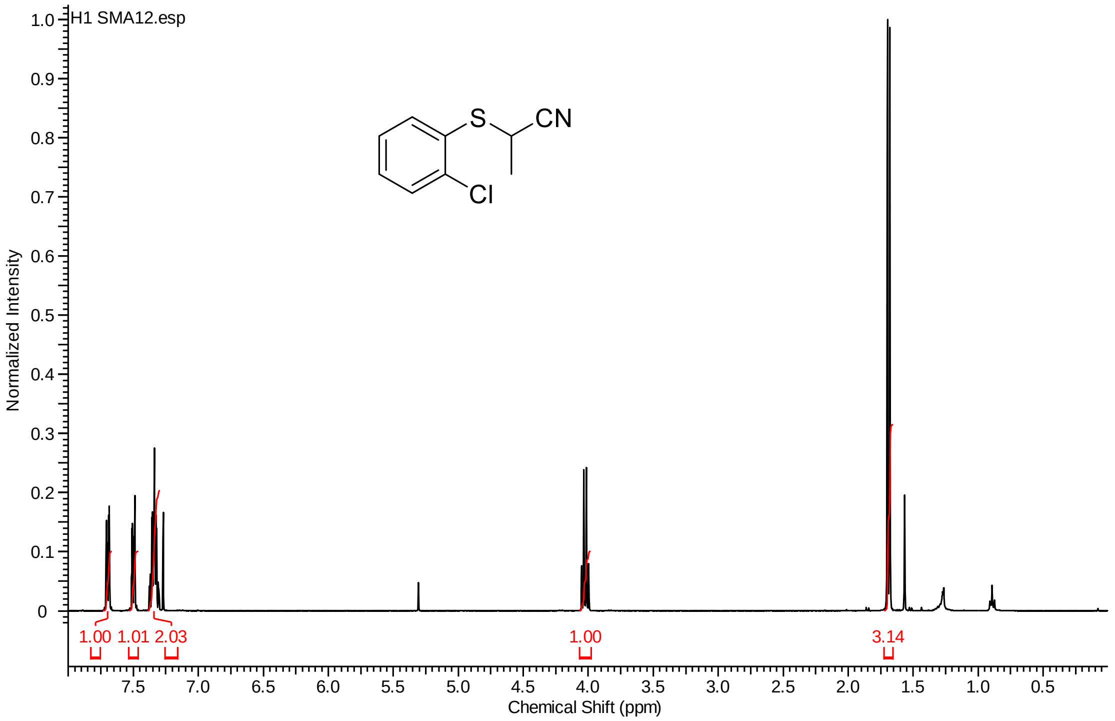

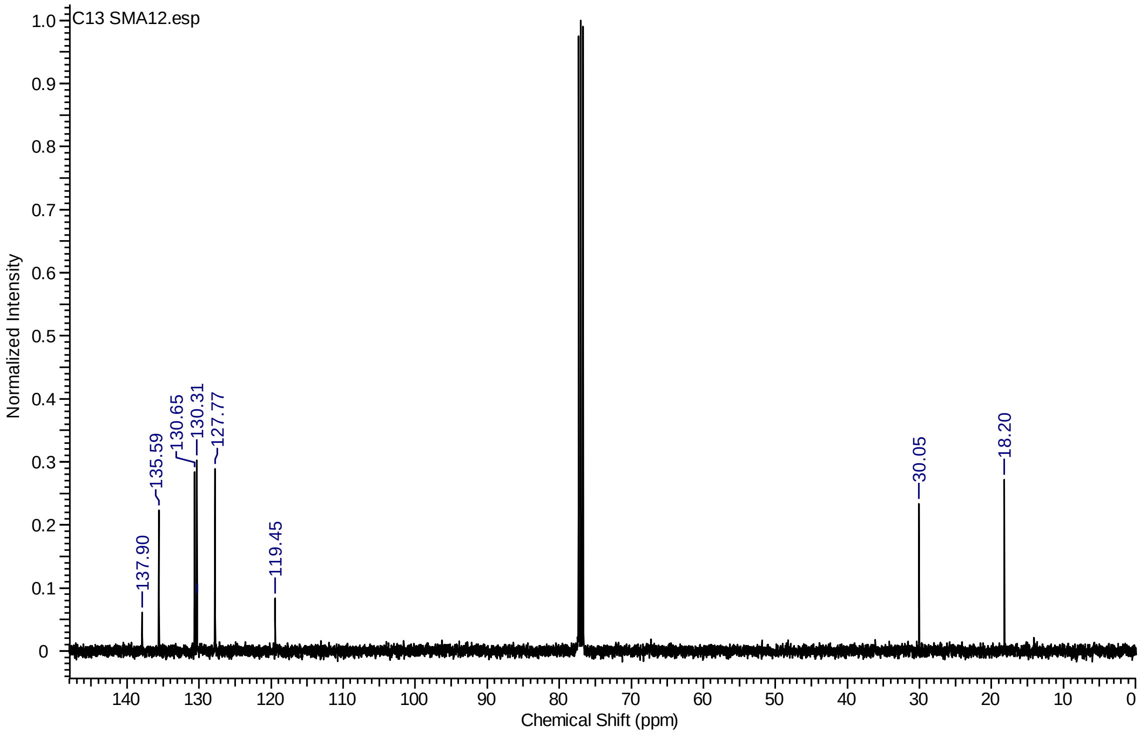

2-((4-Methoxyphenyl)thio)propanenitrile 8e

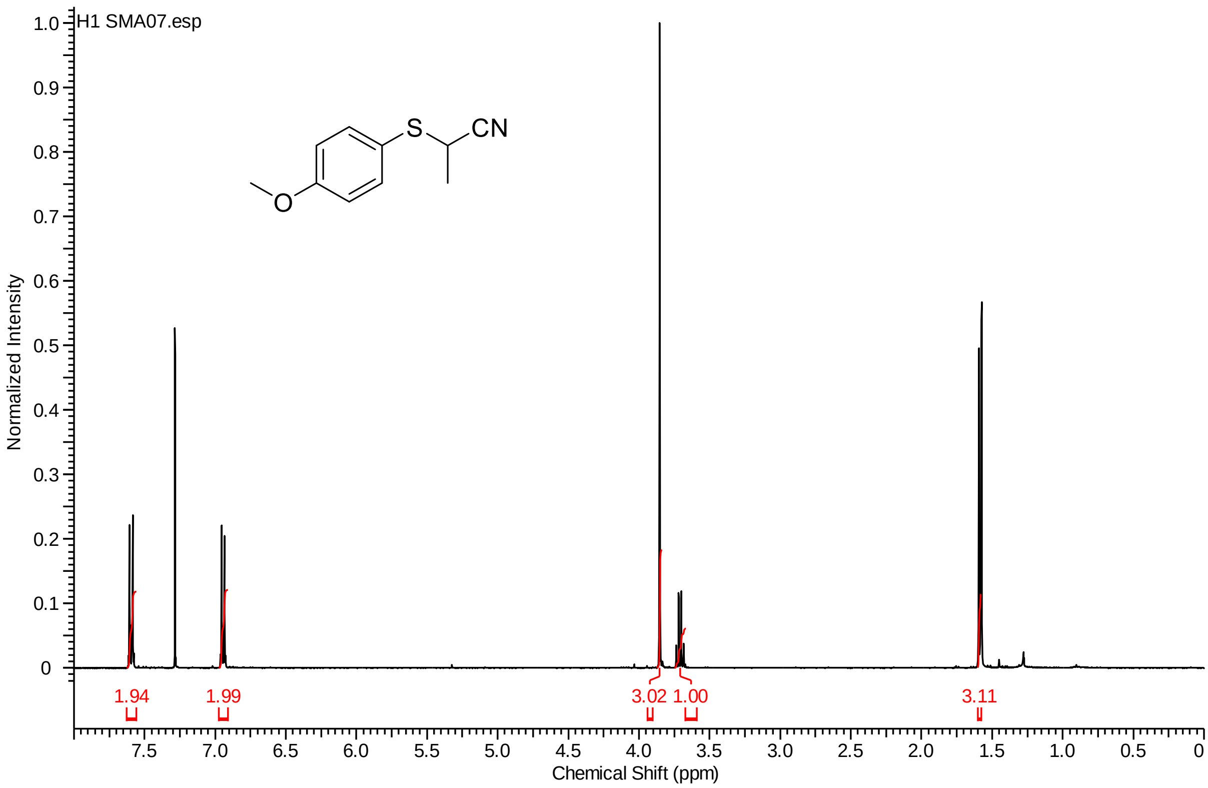

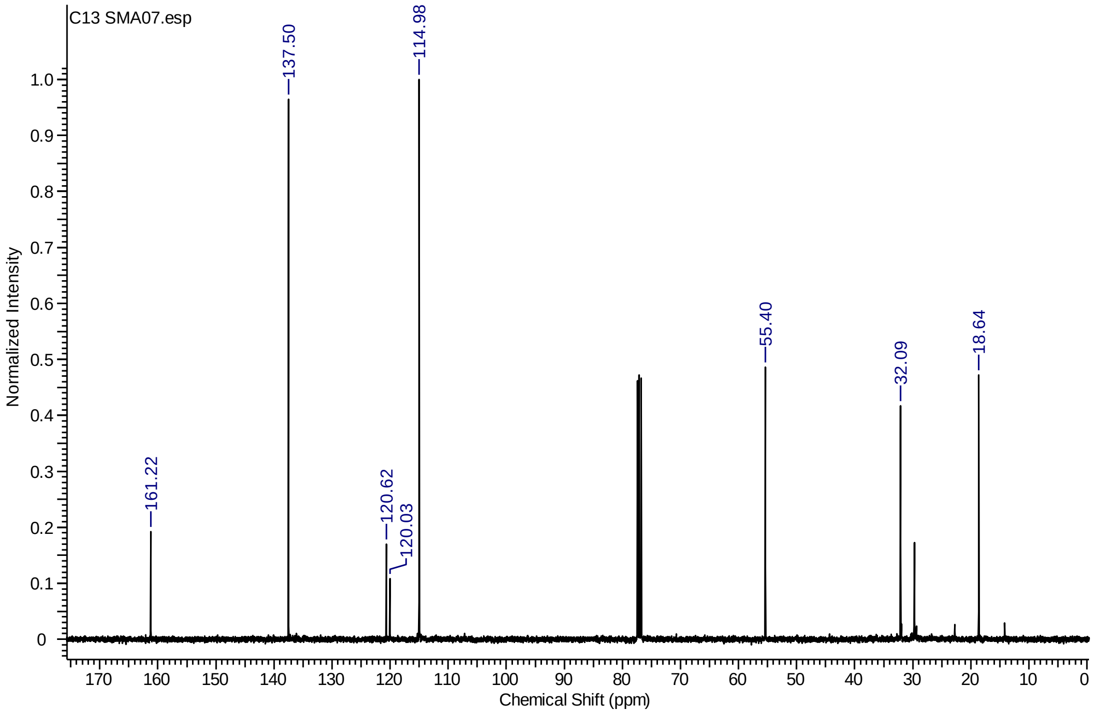

2-((2-Bromophenyl)thio)propanenitrile 8f

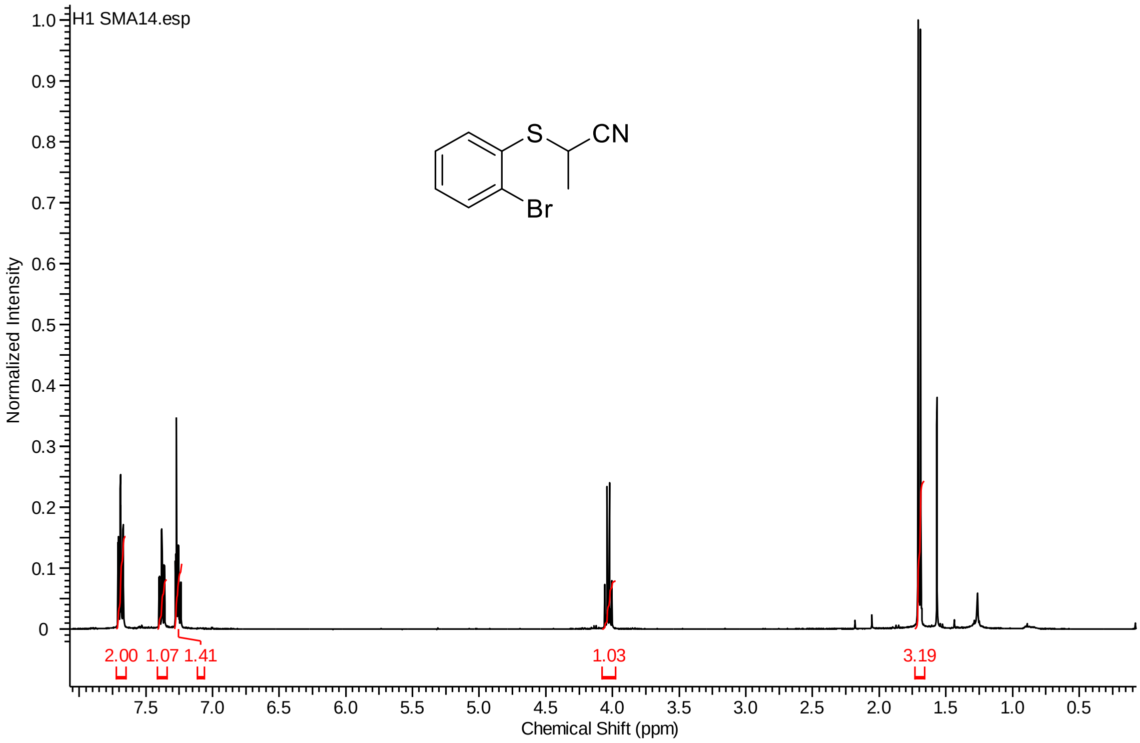

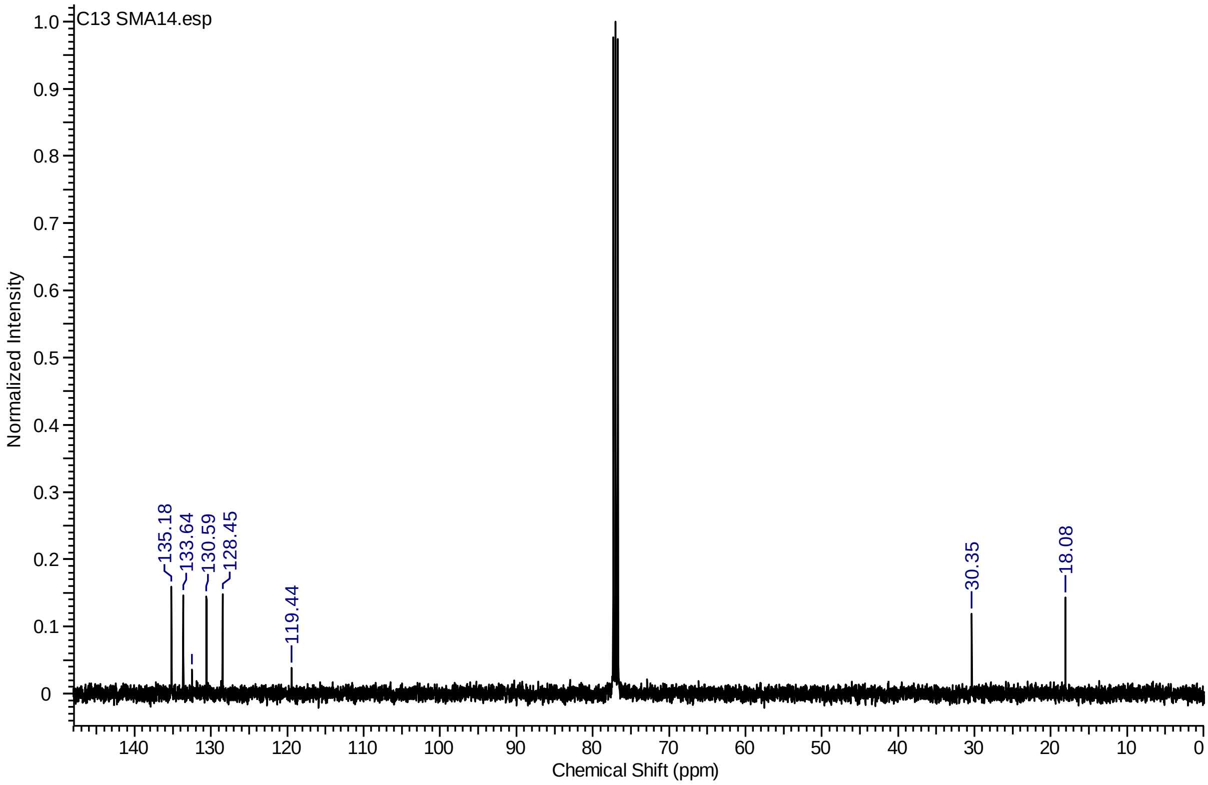

2-(Allylthio)propanenitrile 8g

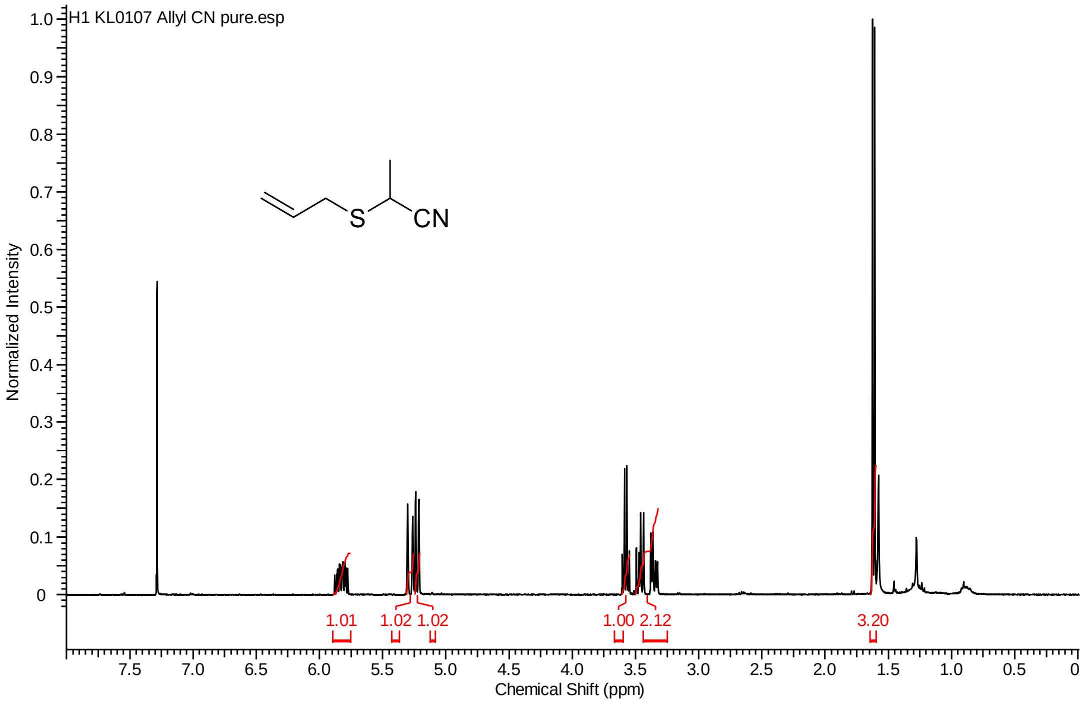

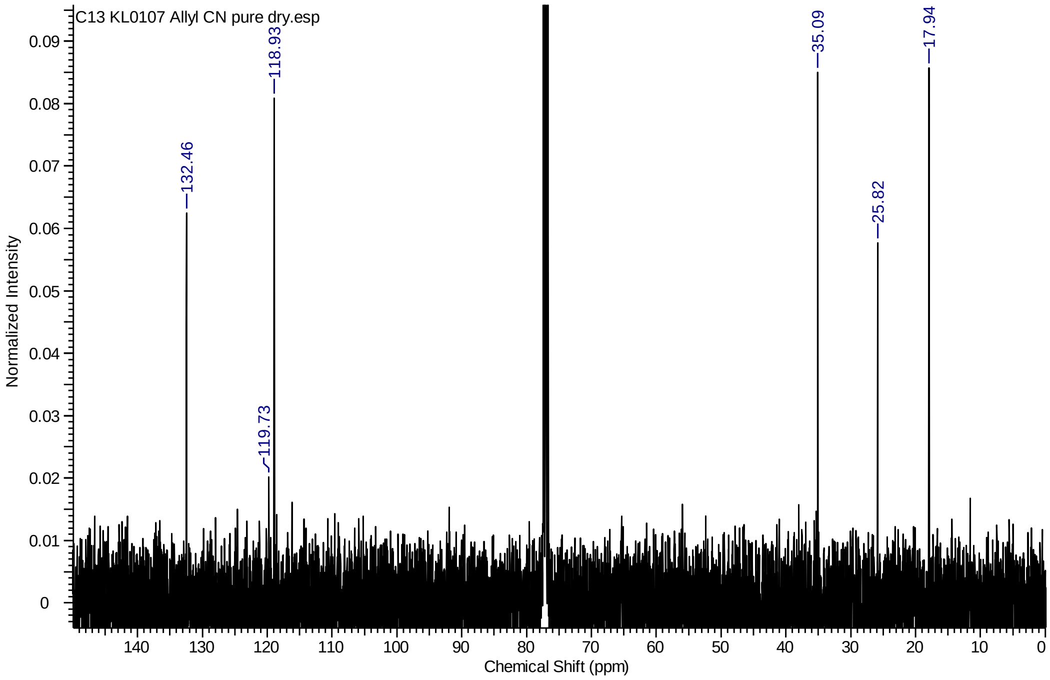

2-(Phenylthio)butanenitrile 8h

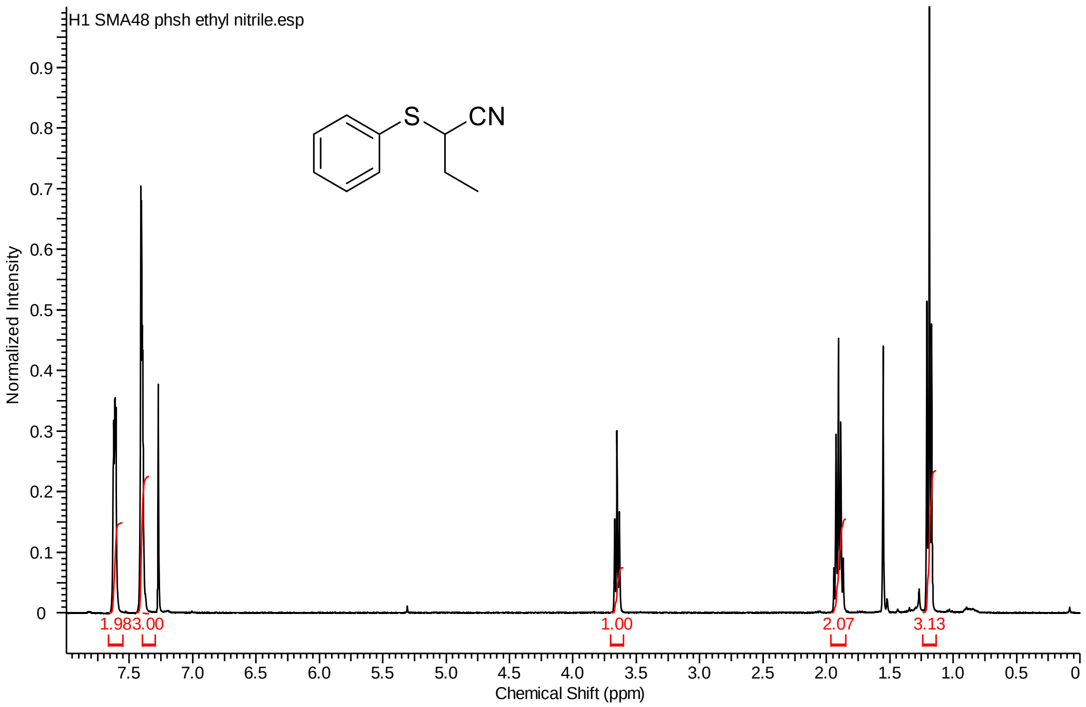

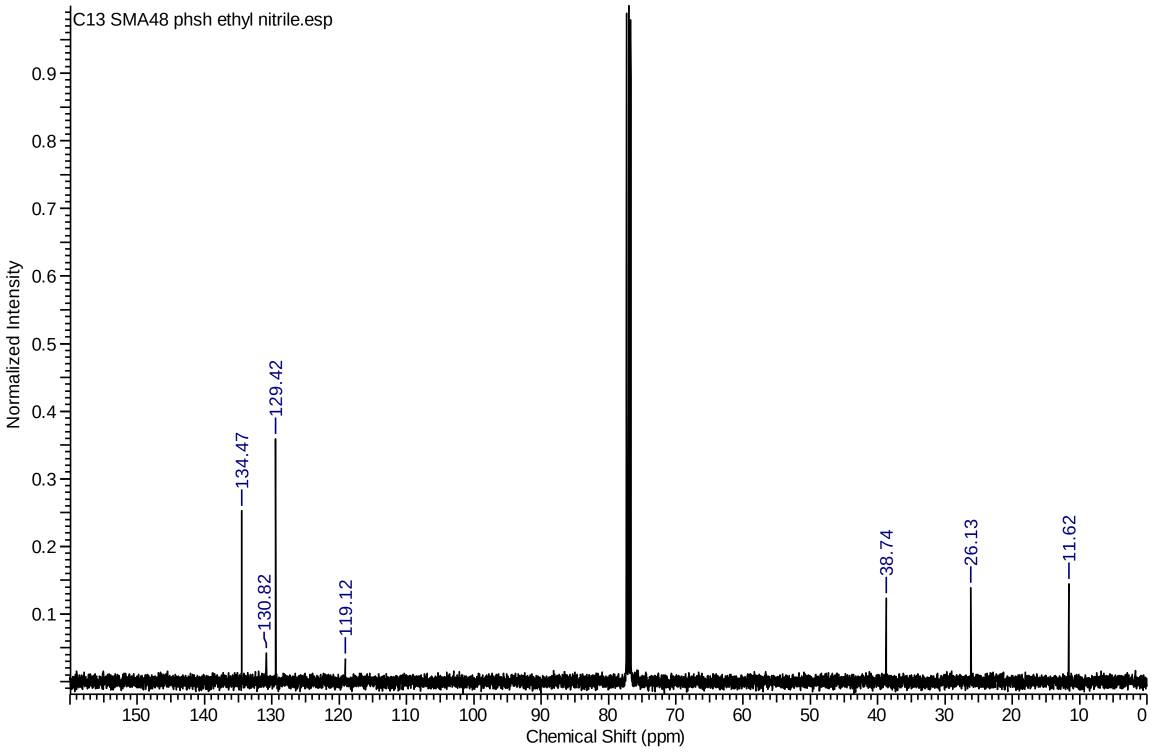

2-((4-Chlorophenyl)thio)butanenitrile 8i

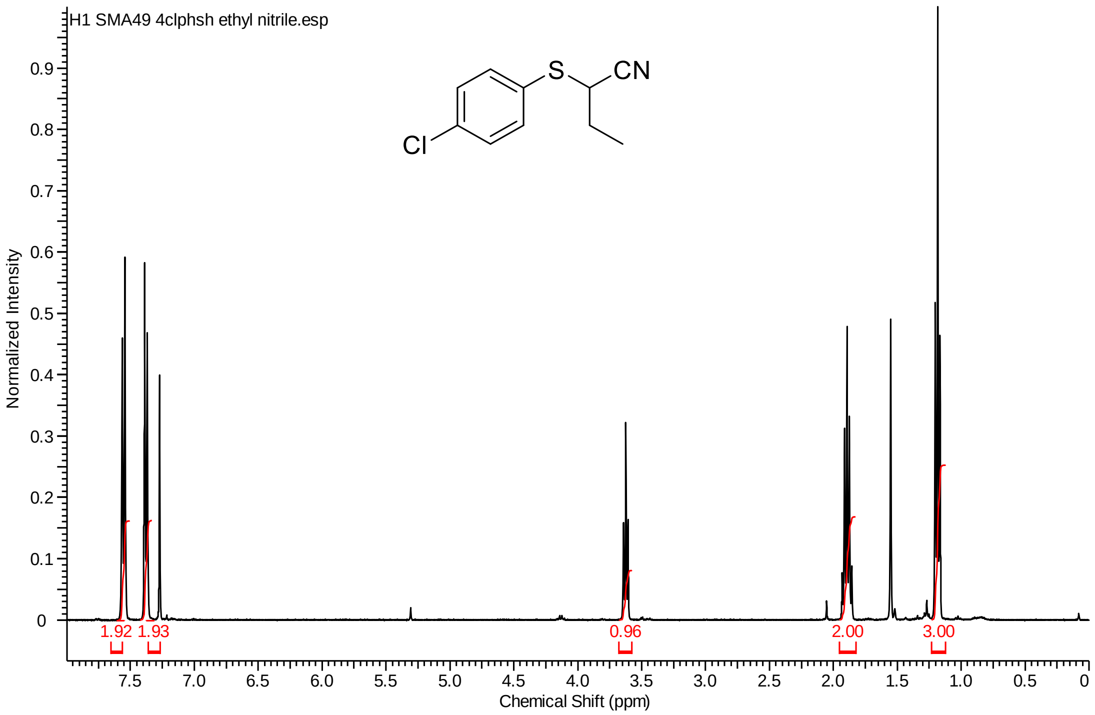

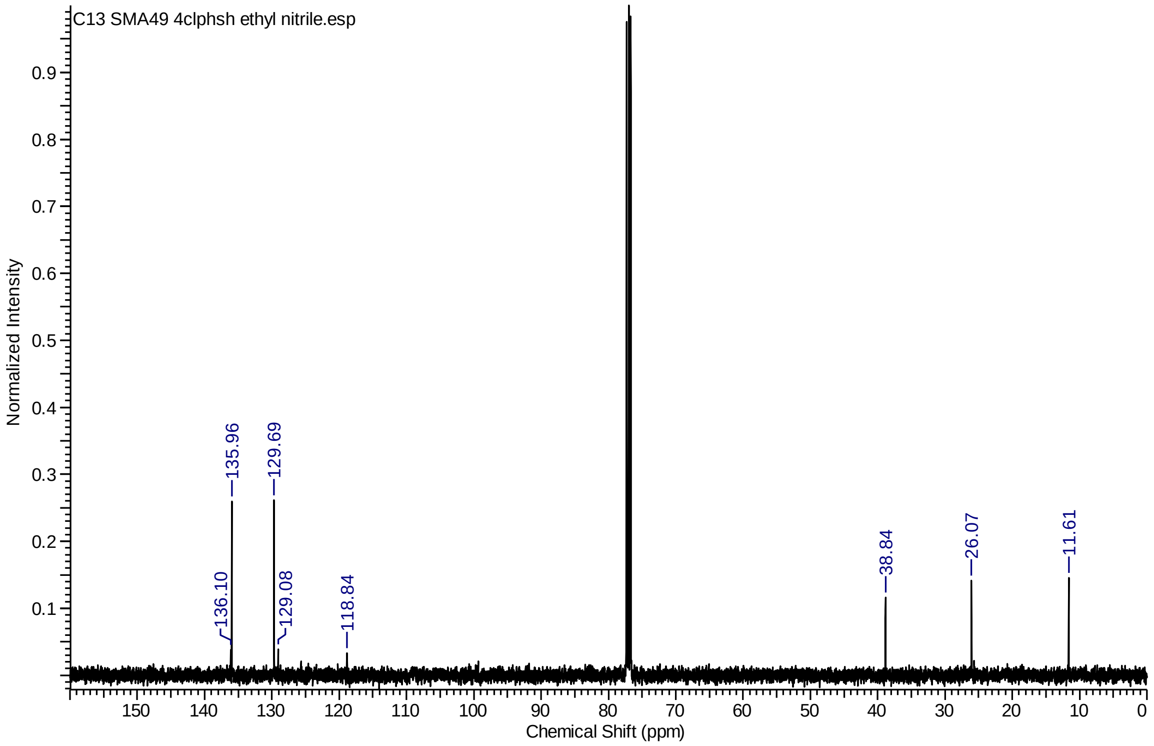

2-(Allylthio)-2-phenylacetonitrile 8j

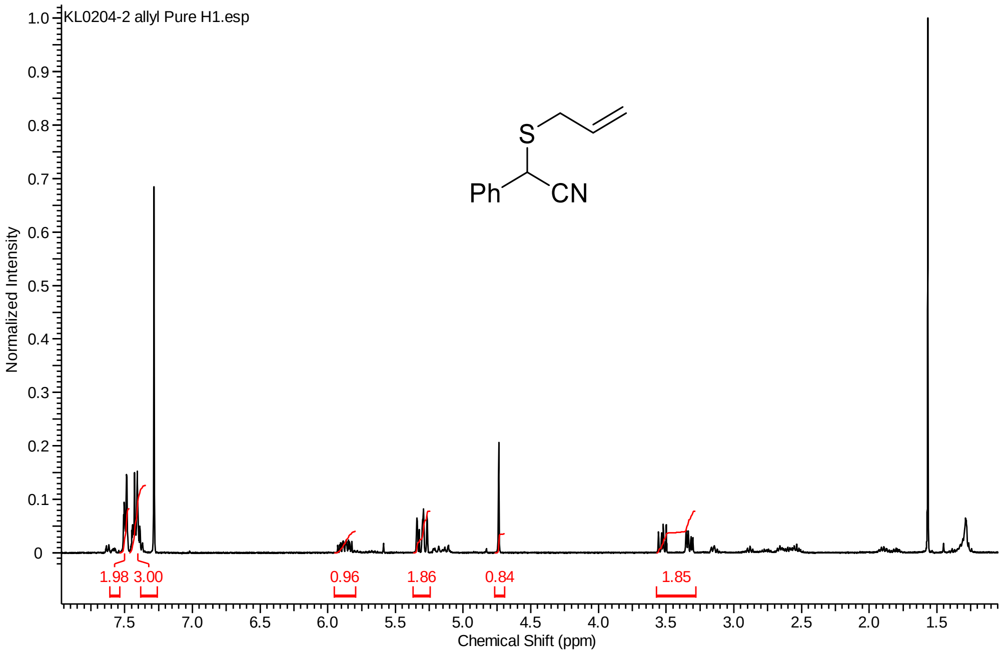

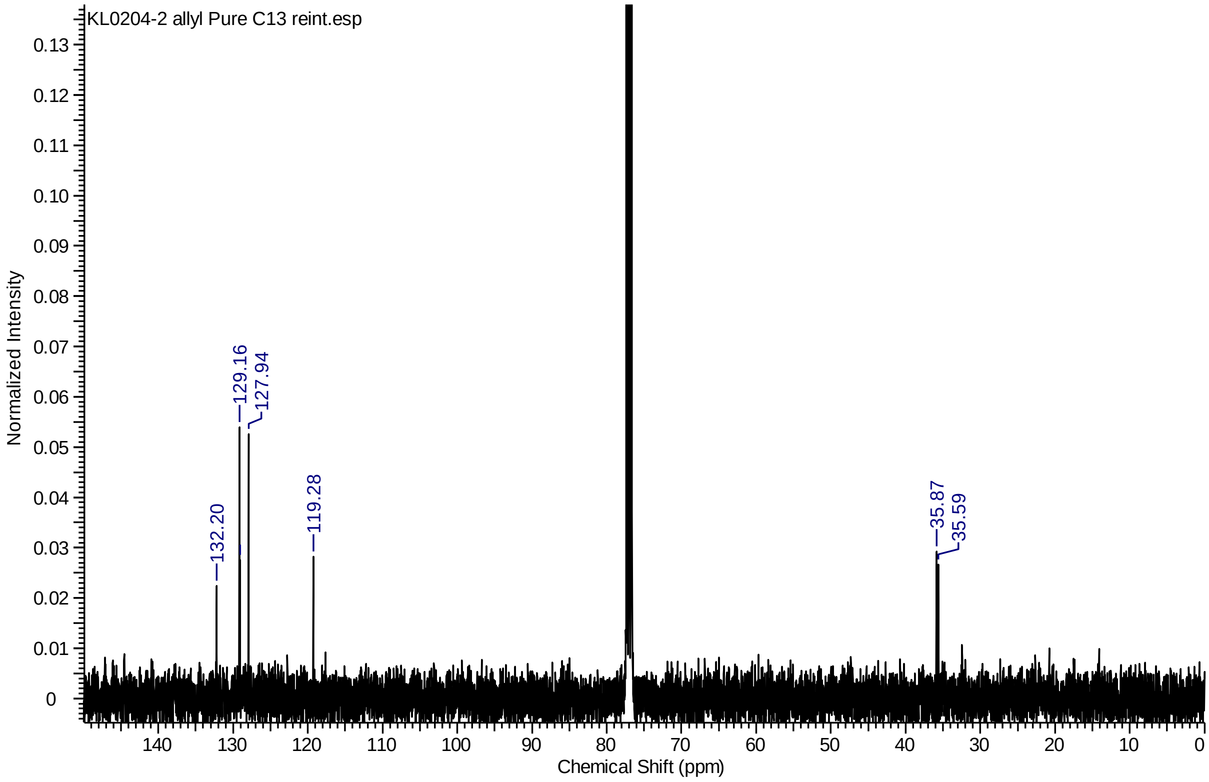

2-(Phenylthio)pentanenitrile 8k

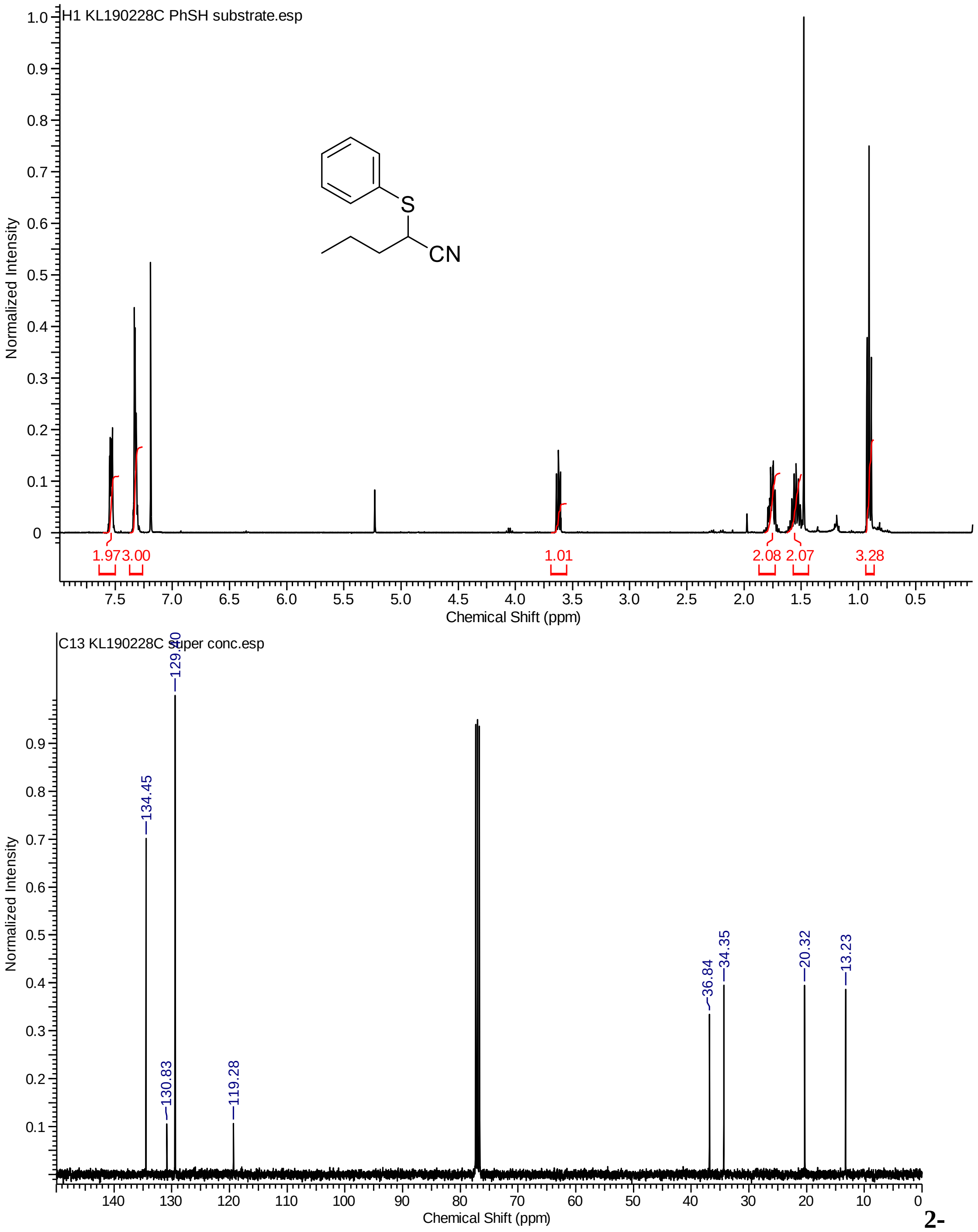

2-

2-(*p*-Tolylthio)pentanenitrile 8l

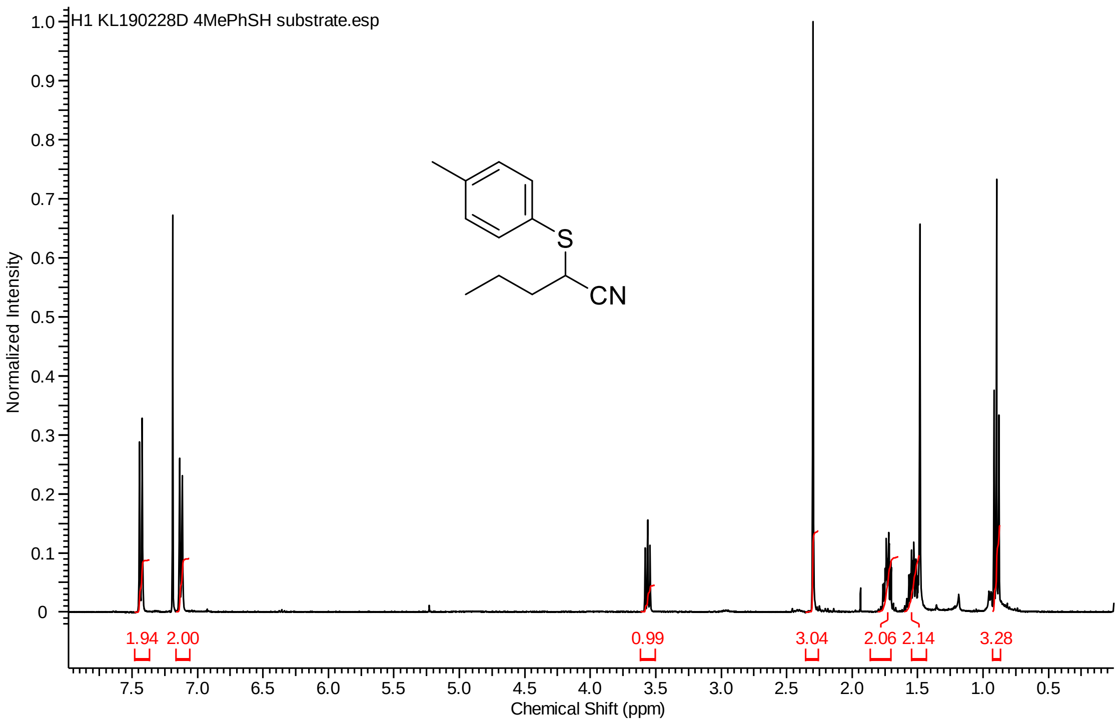

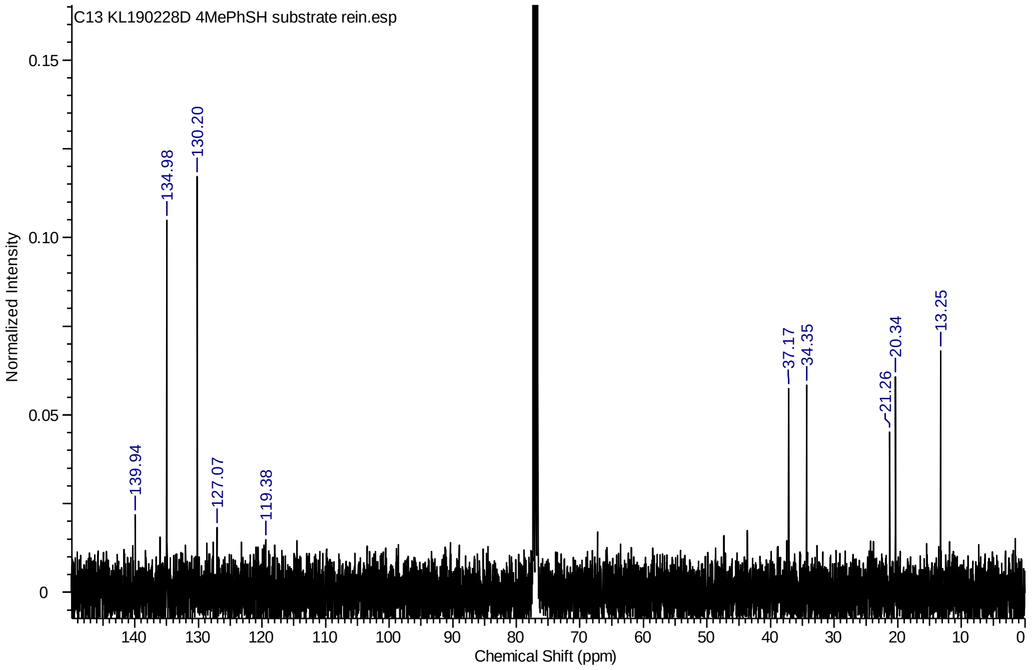

3-Methyl-2-(phenylthio)butanenitrile 8m

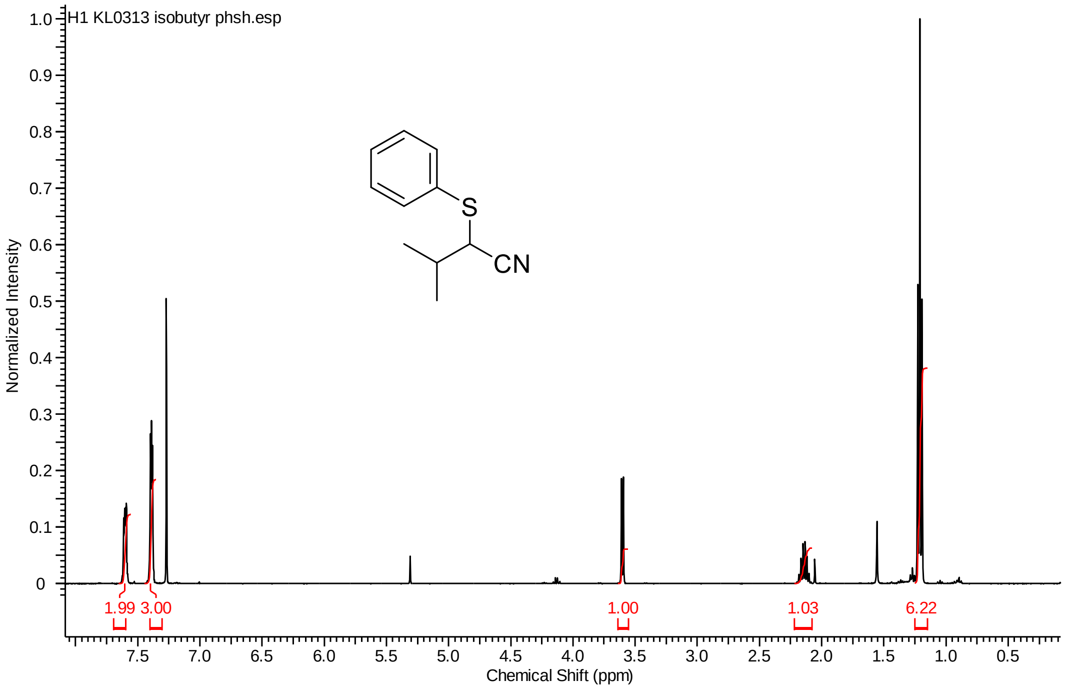

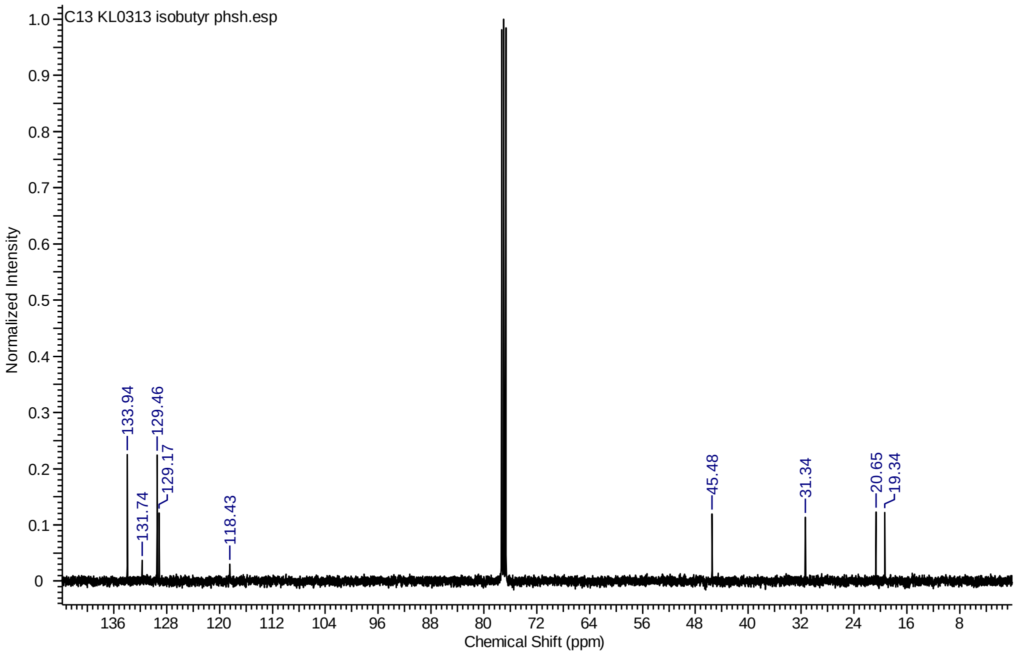

2-((4-Chlorophenyl)thio)pentanenitrile 8n

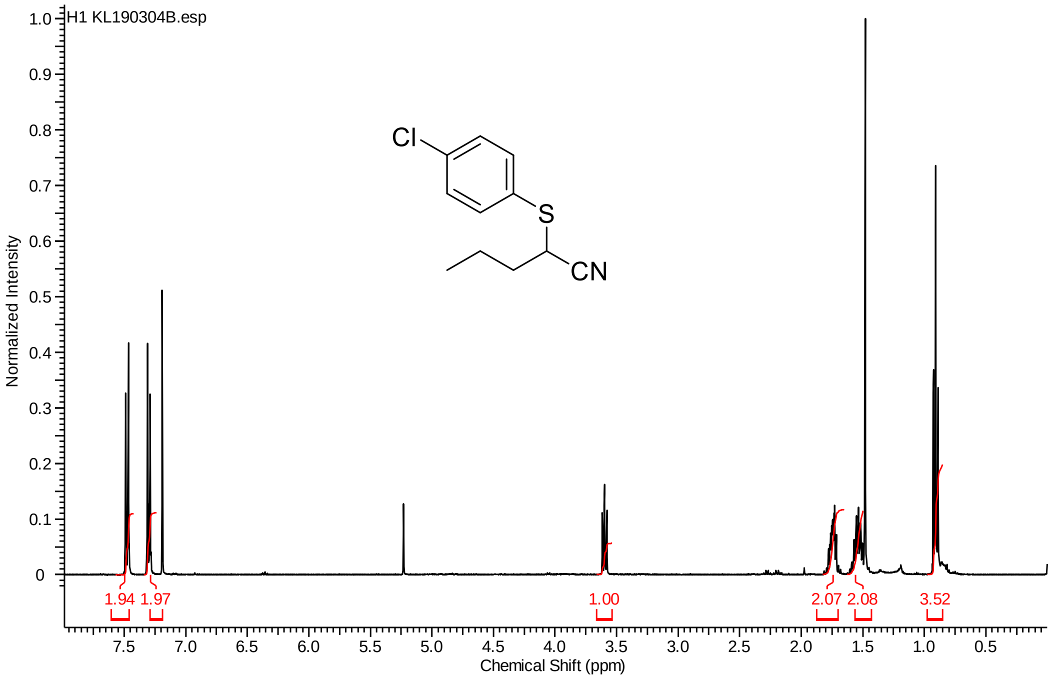

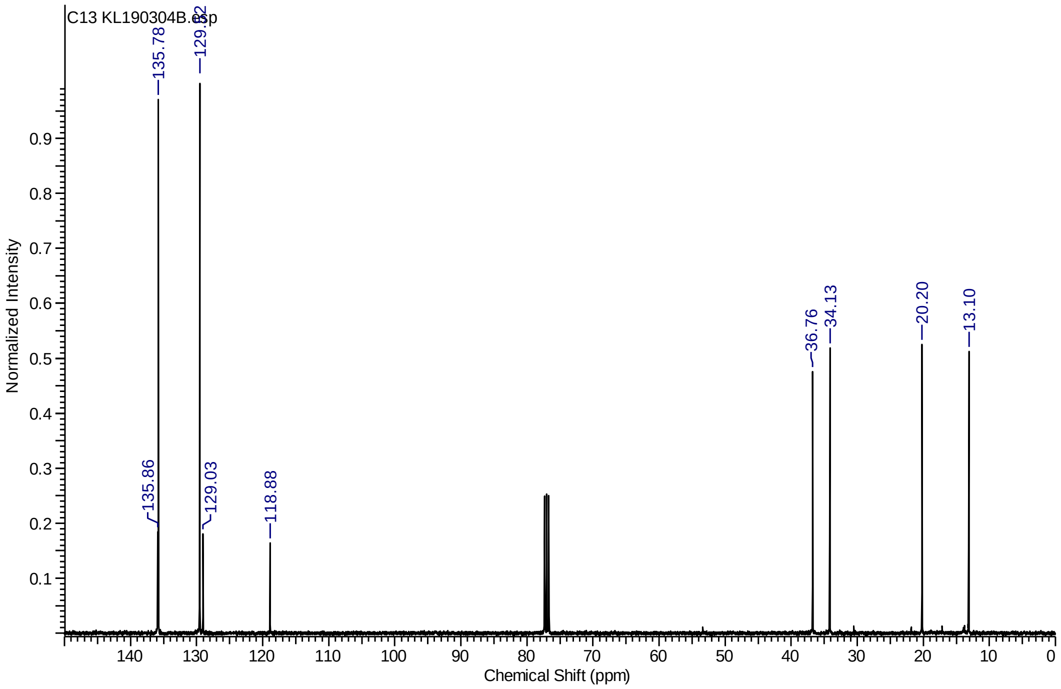

2-((4-Chlorophenyl)thio)-3-methylbutanenitrile 8o

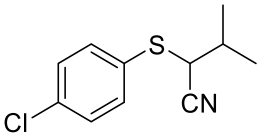

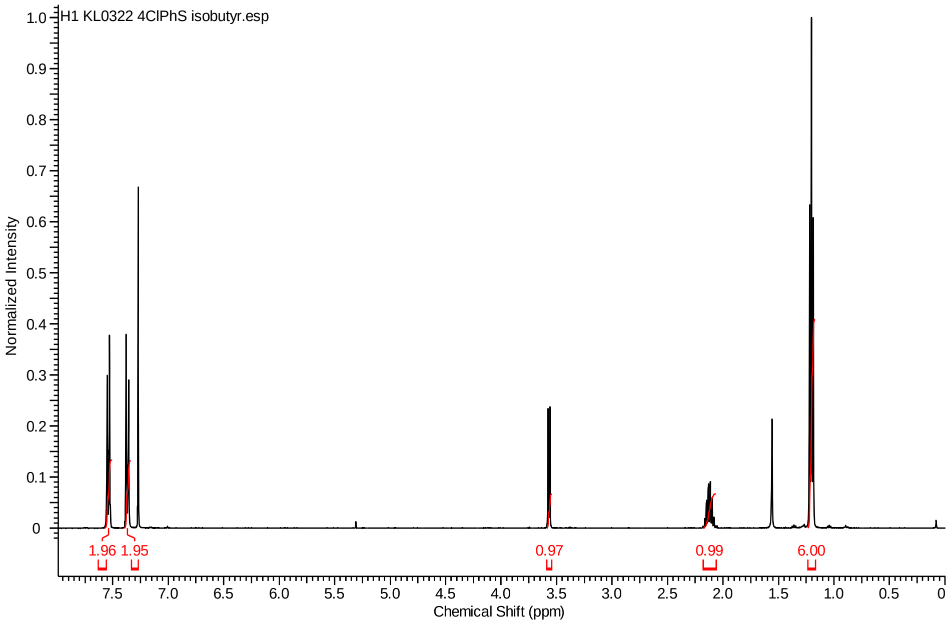

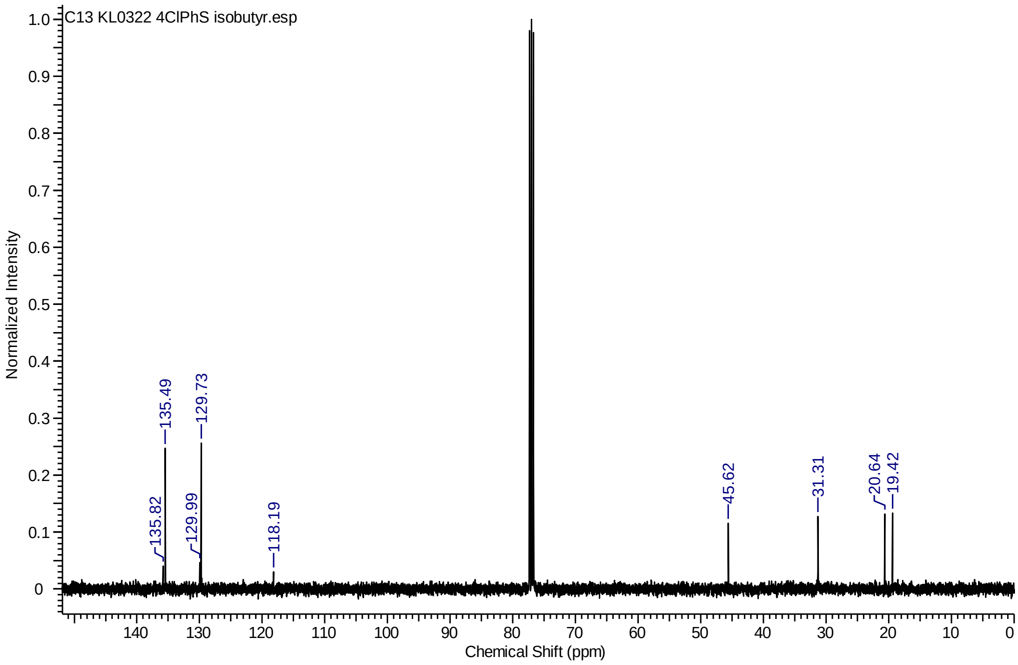

Phenyl-2-(propylthio)acetonitrile 8p

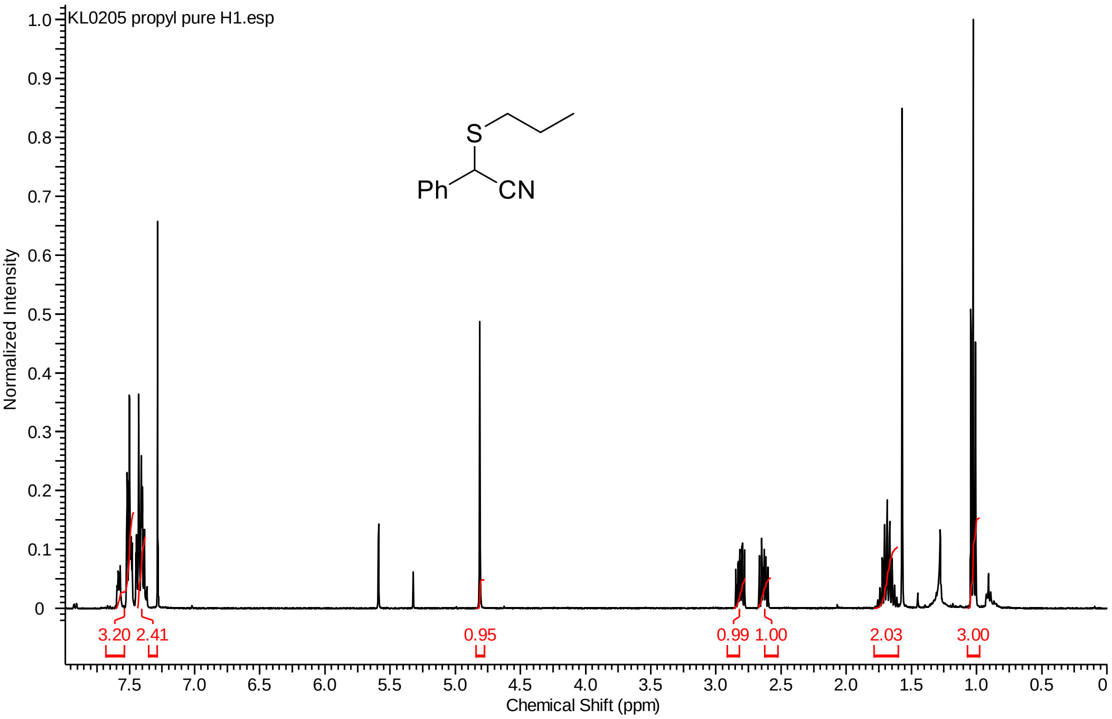

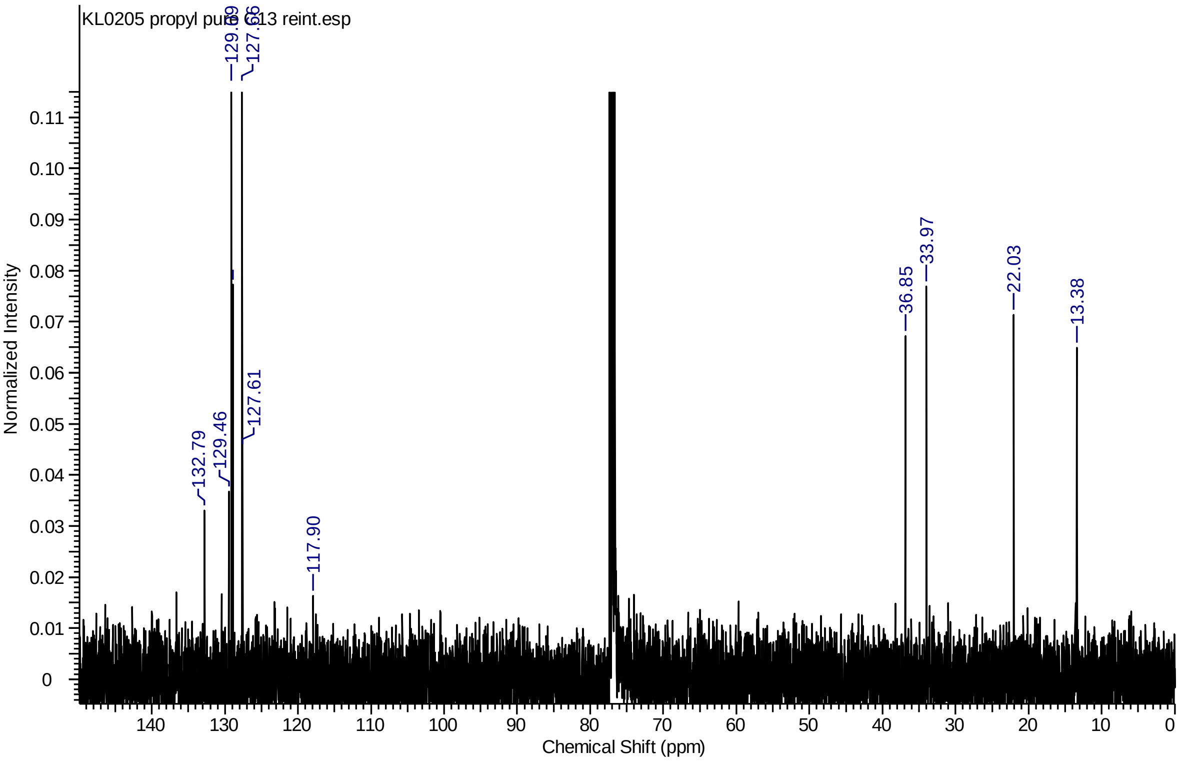

2-(Phenylthio)propanoic acid 9a

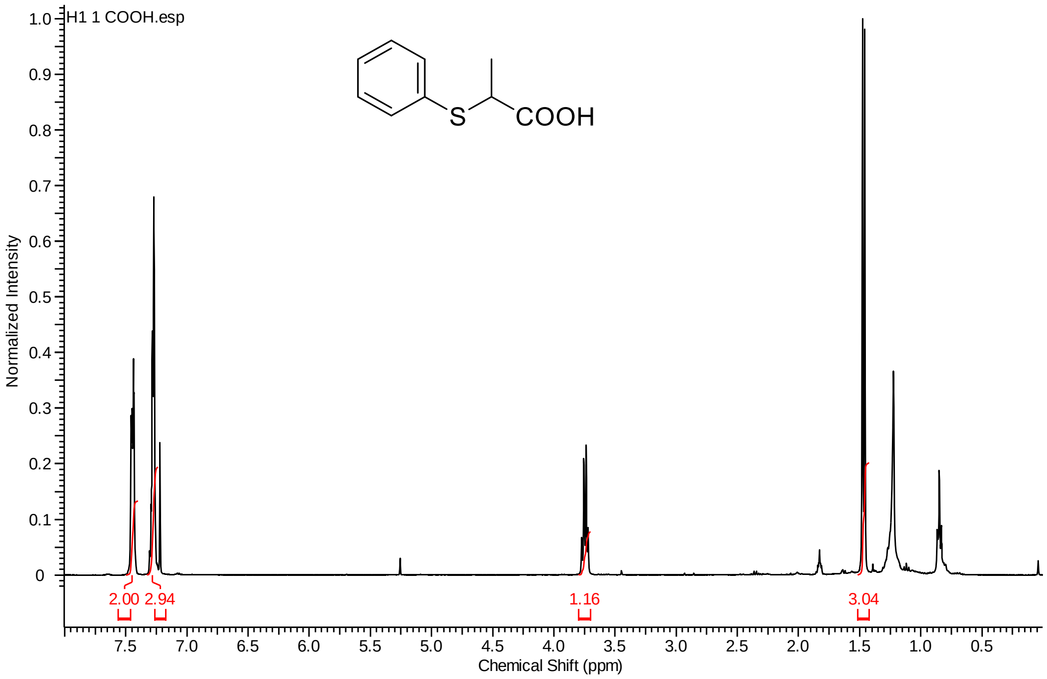

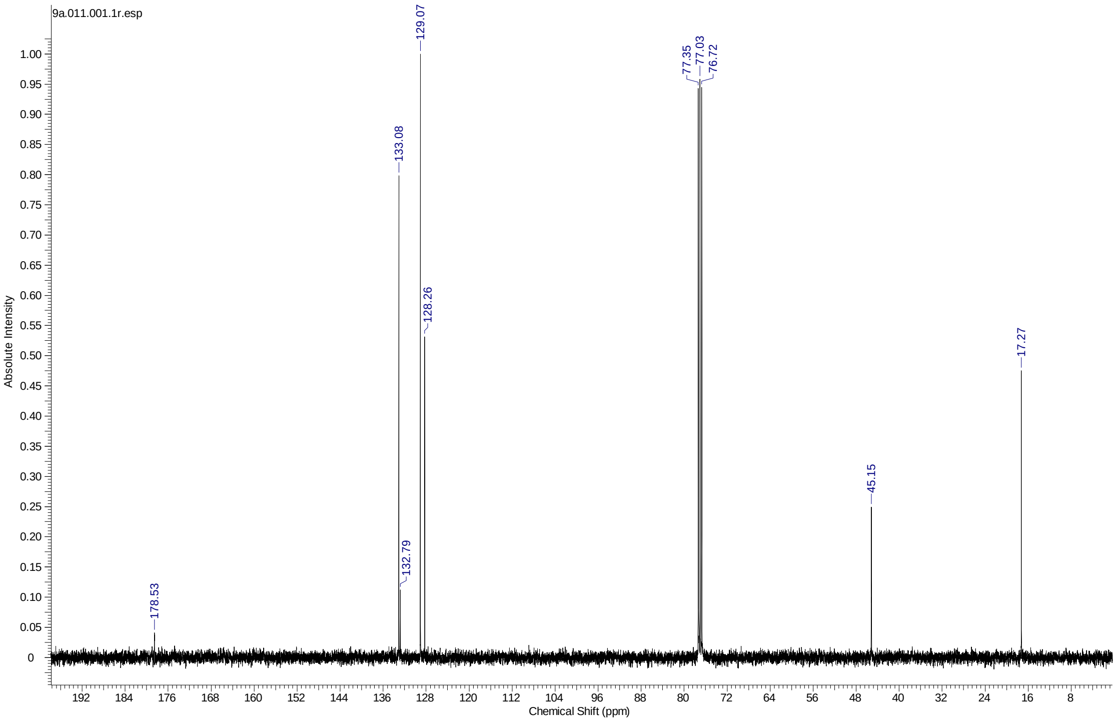

2-(*p*-Tolylthio)propanoic acid 9b

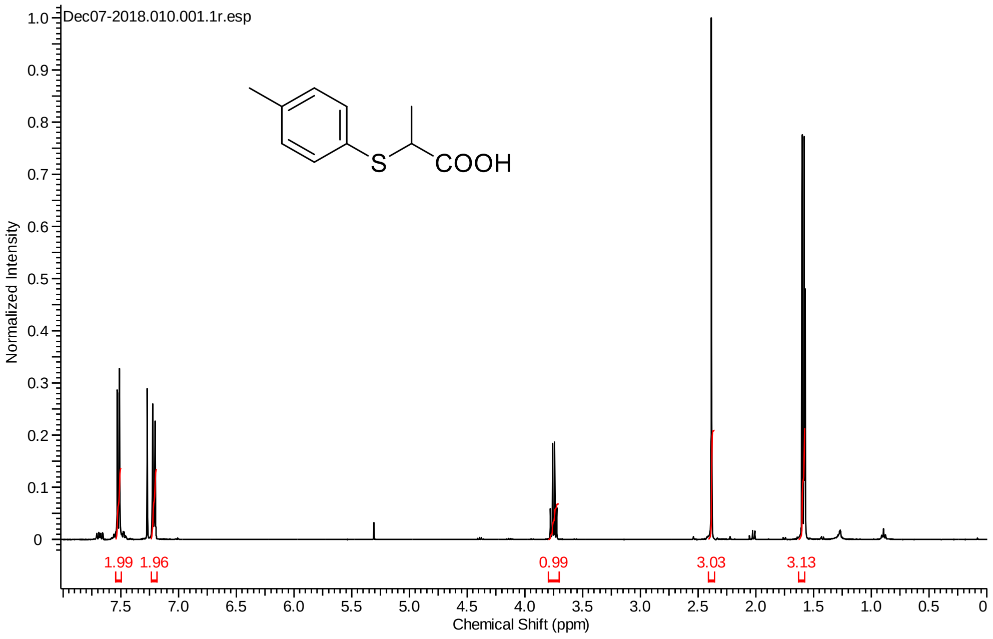
Dec07-2018.010.001.1r.esp
S
COOH
Normalized Intensity
Chemical Shift (ppm)
1.99
1.96
0.99
3.03
3.13

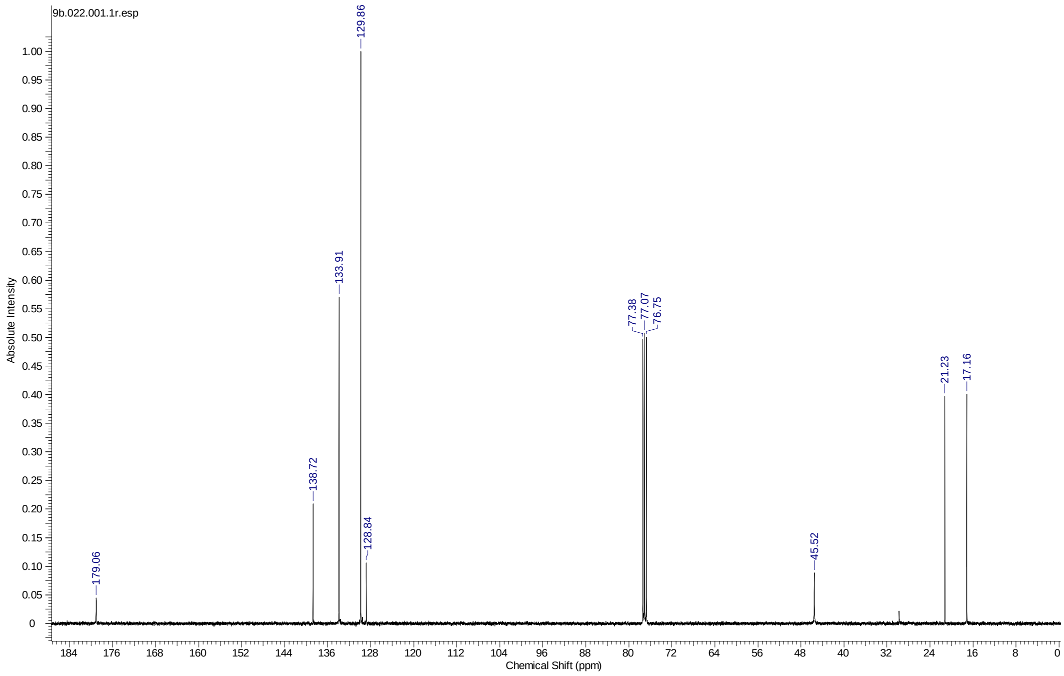
9b.022.001.1r.esp
Absolute Intensity
Chemical Shift (ppm)
179.06
138.72
133.91
129.86
128.84
77.38
77.07
76.75
45.52
21.23
17.16

2-((4-Chlorophenyl)thio)propanoic acid 9c

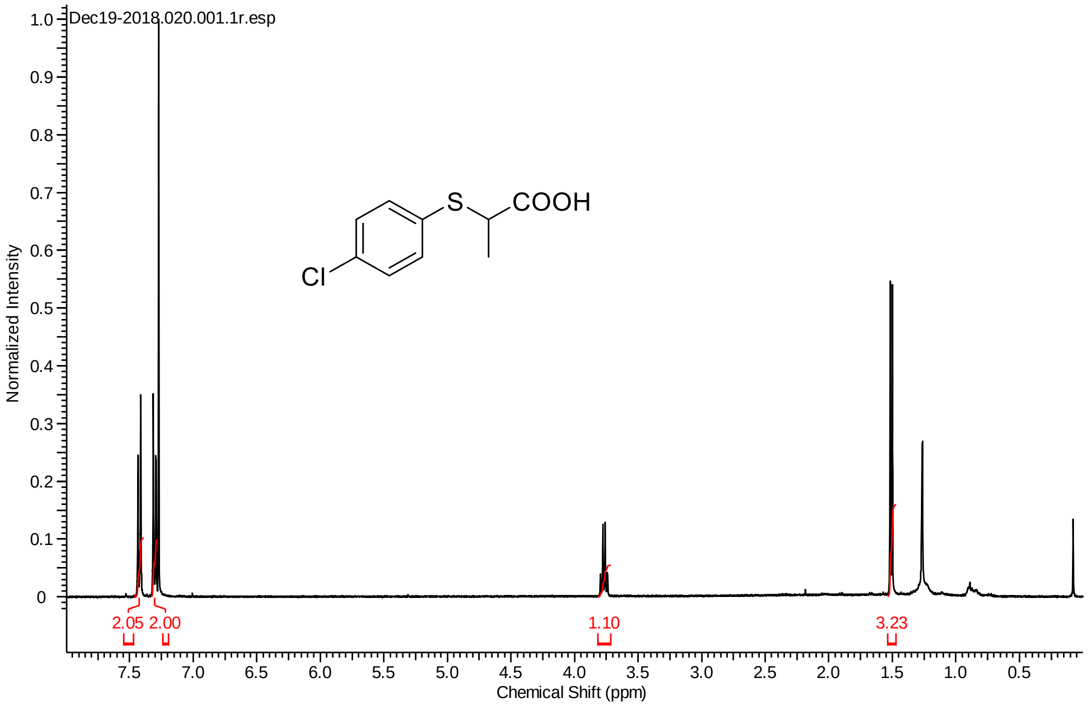

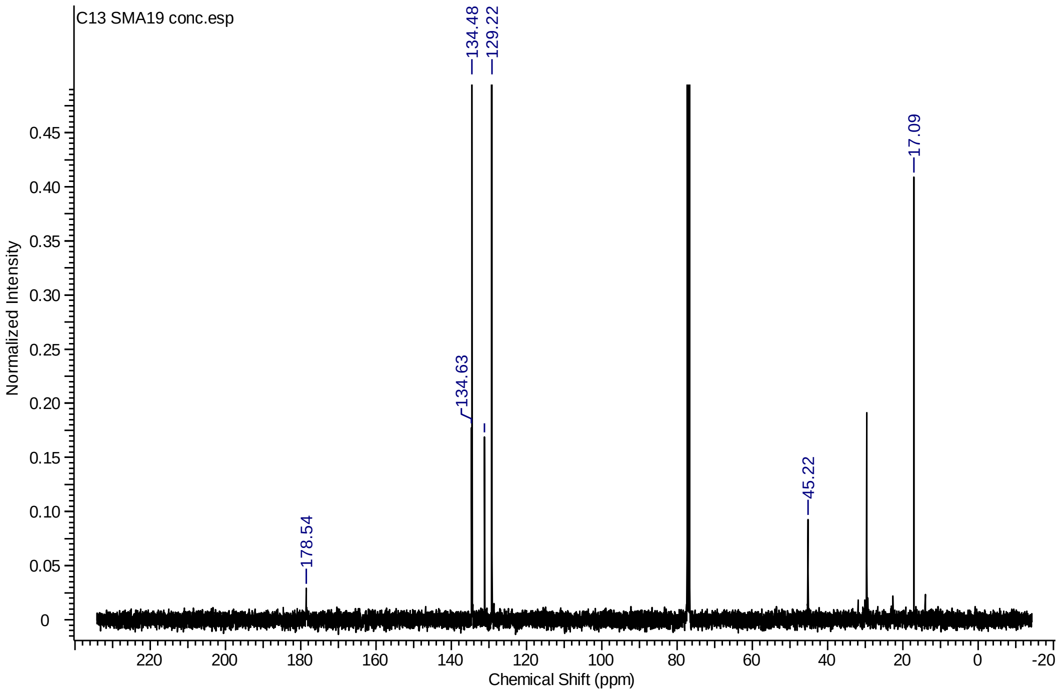

2-((2-Chlorophenyl)thio)propanoic acid 9d

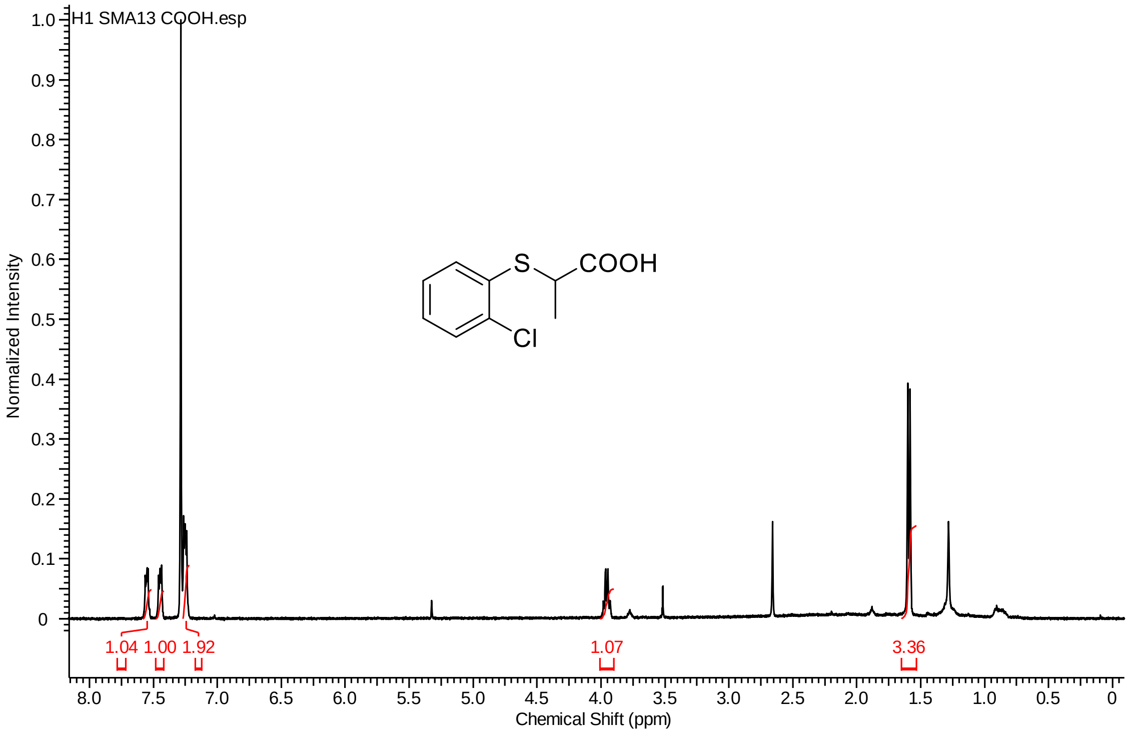

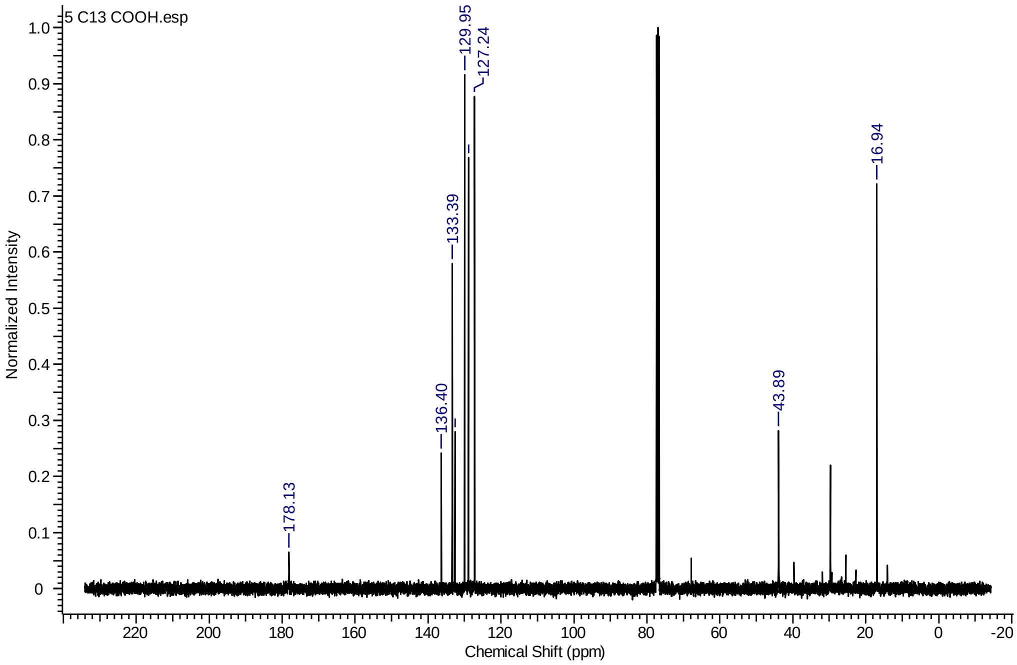

2-((4-Methoxyphenyl)thio)propanoic acid 9e

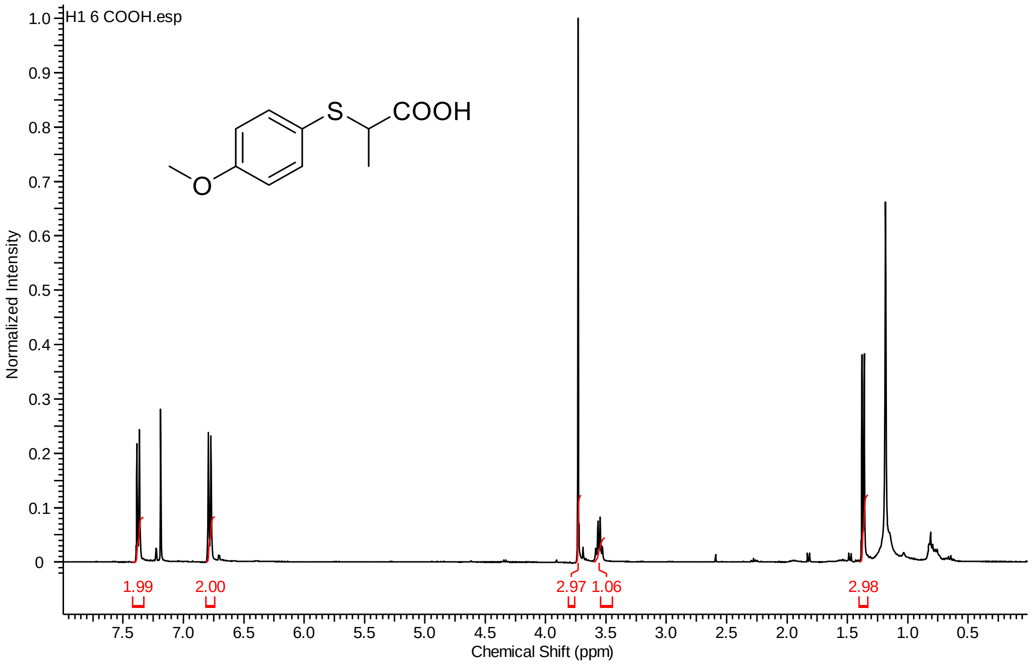

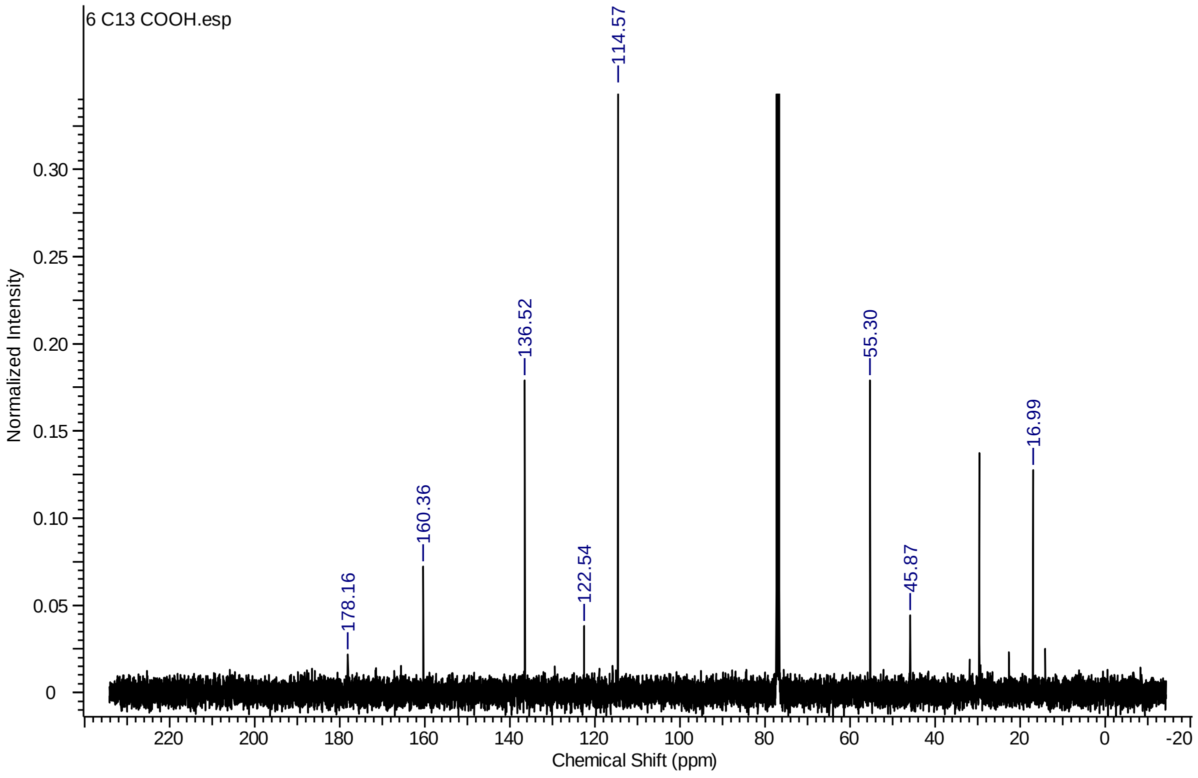

2-((2-Bromophenyl)thio)propanoic acid 9f

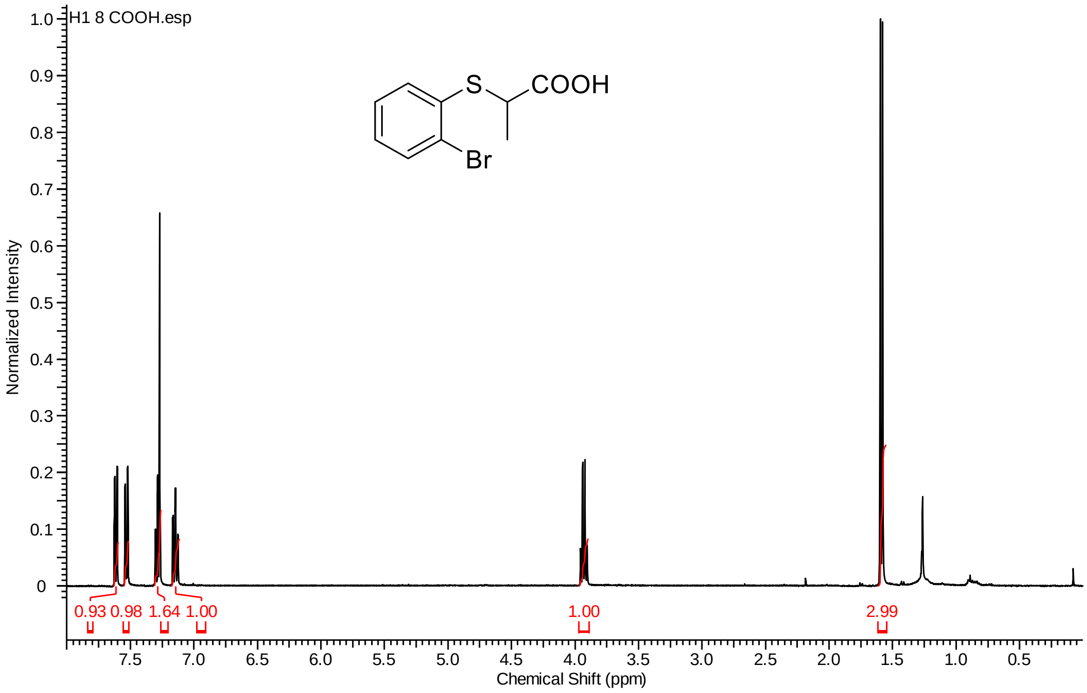

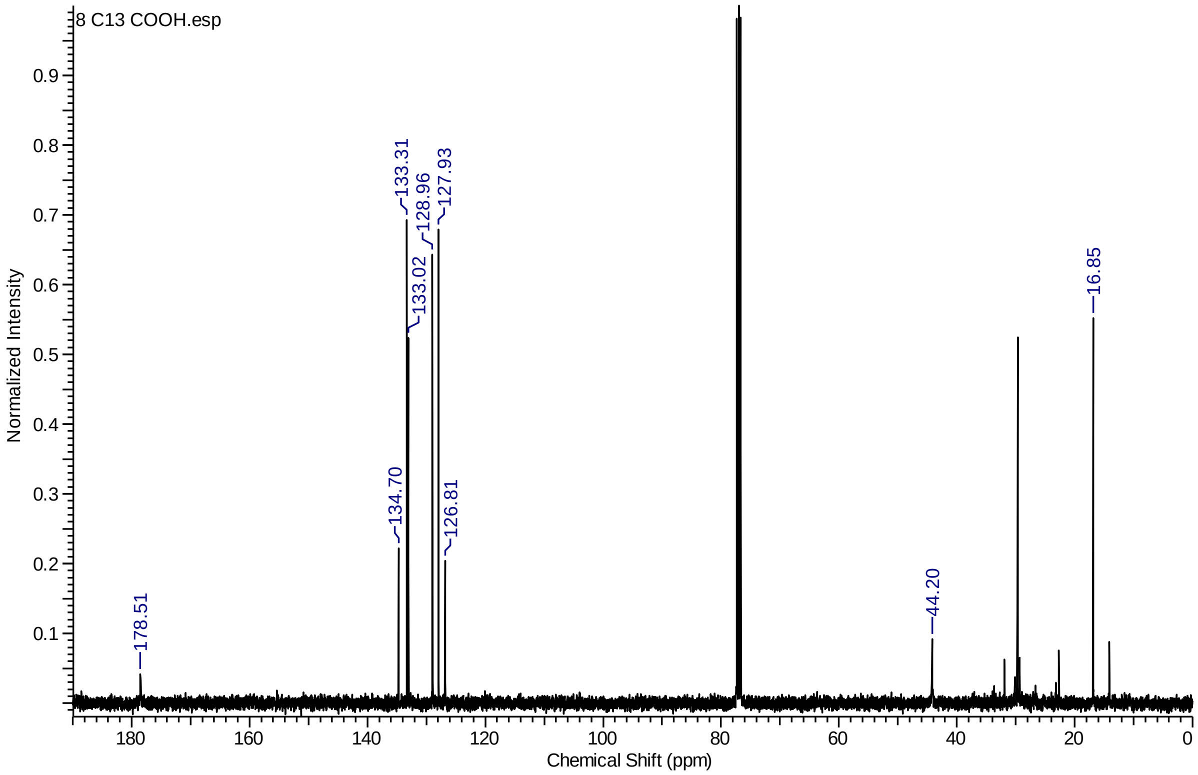

2-(Allylthio)propanoic acid 9g

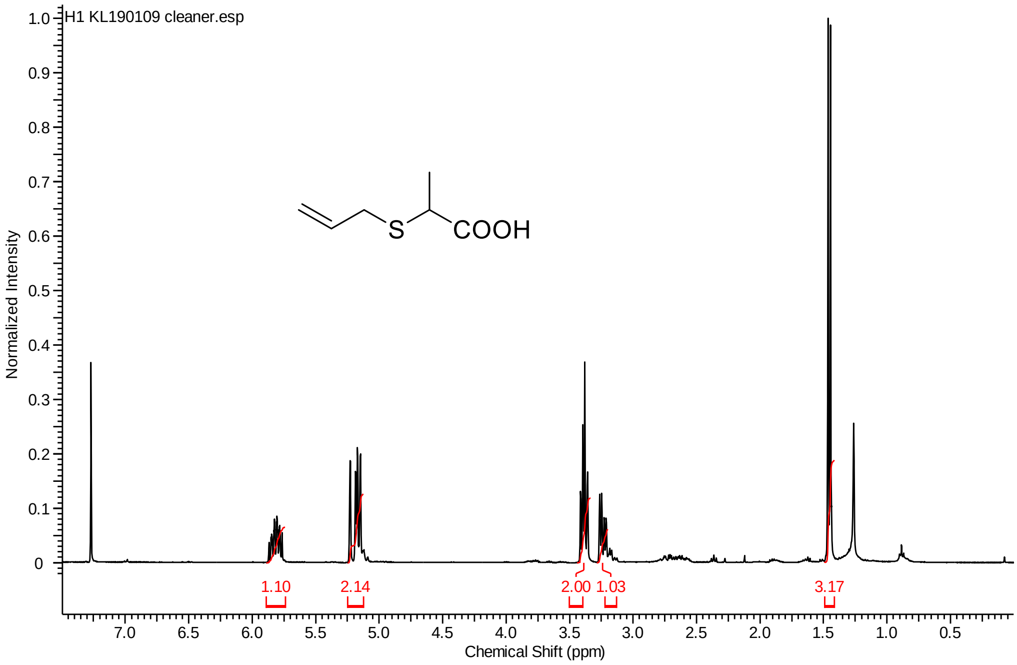

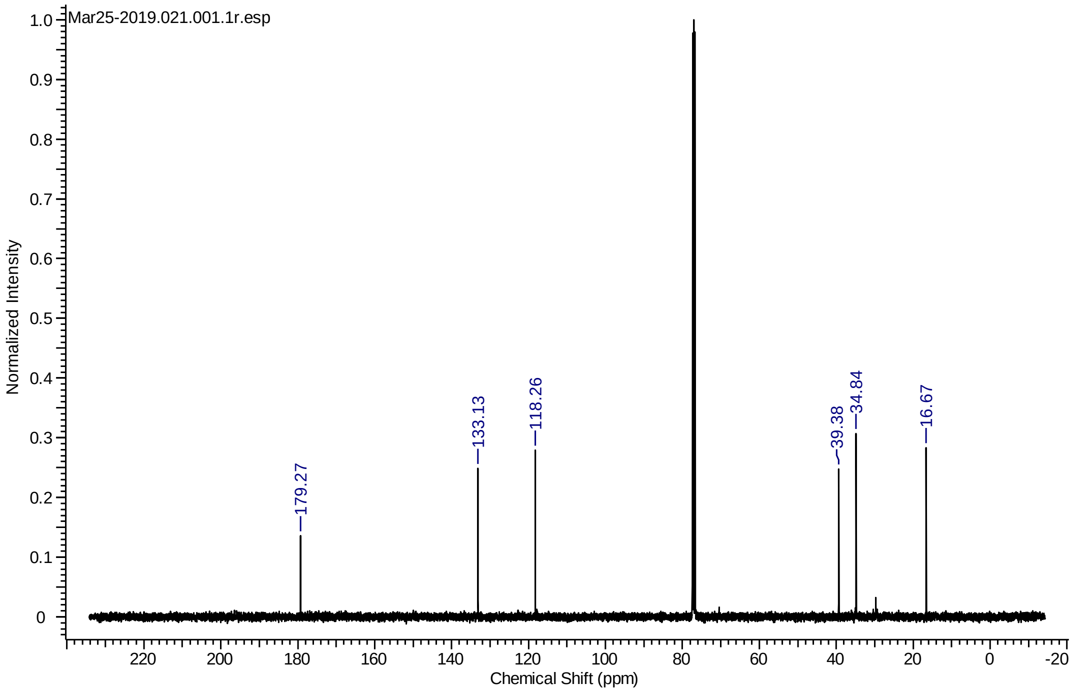

2-(Phenylthio)butanoic acid 9h

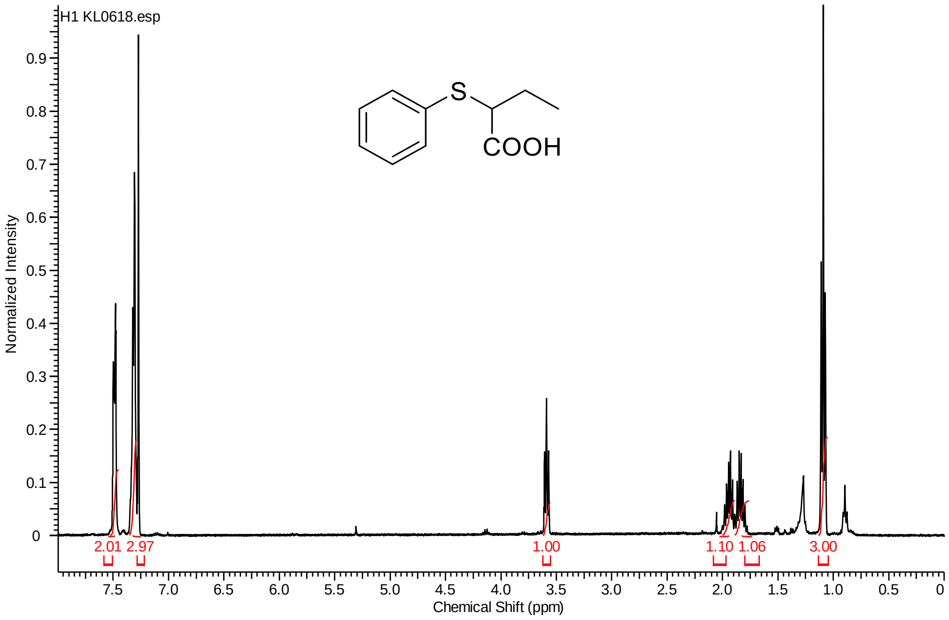

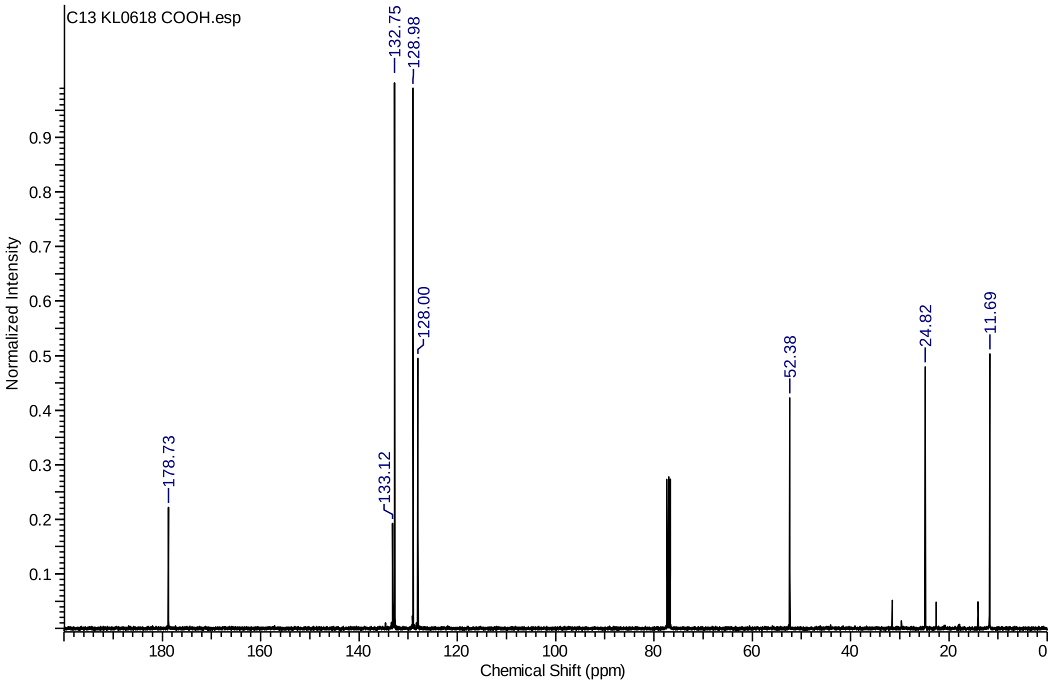

2-((4-Chlorophenyl)thio)butanoic acid 9i

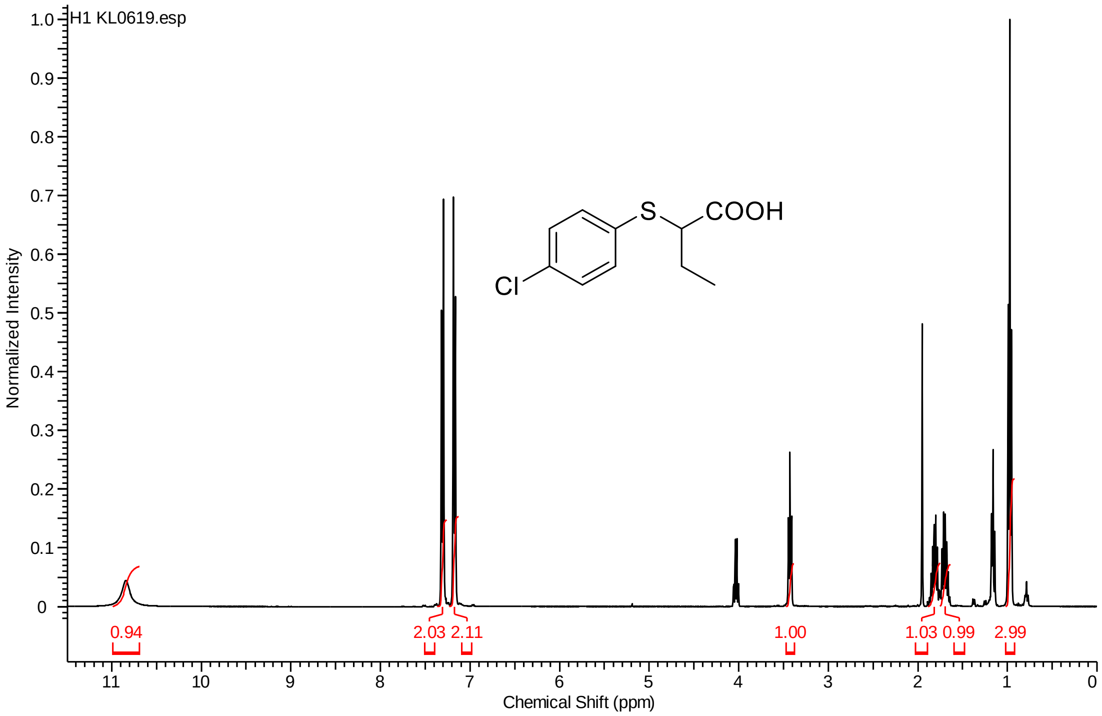

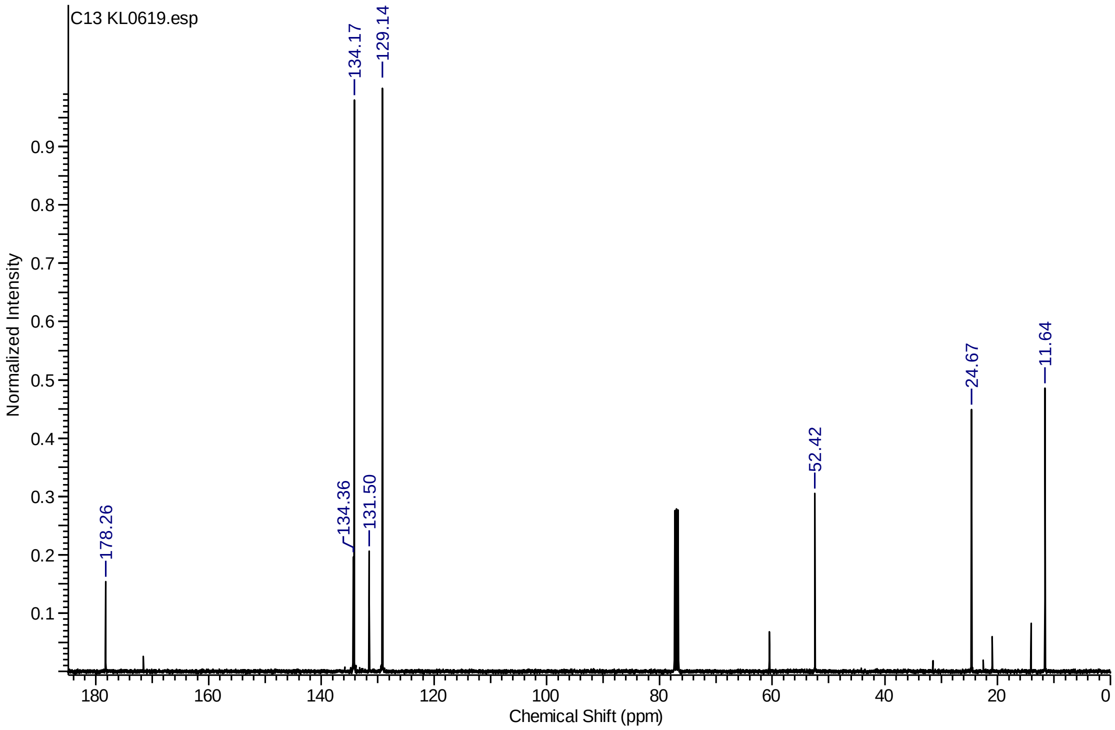

2-(Allylthio)-2-phenylacetic acid 9j

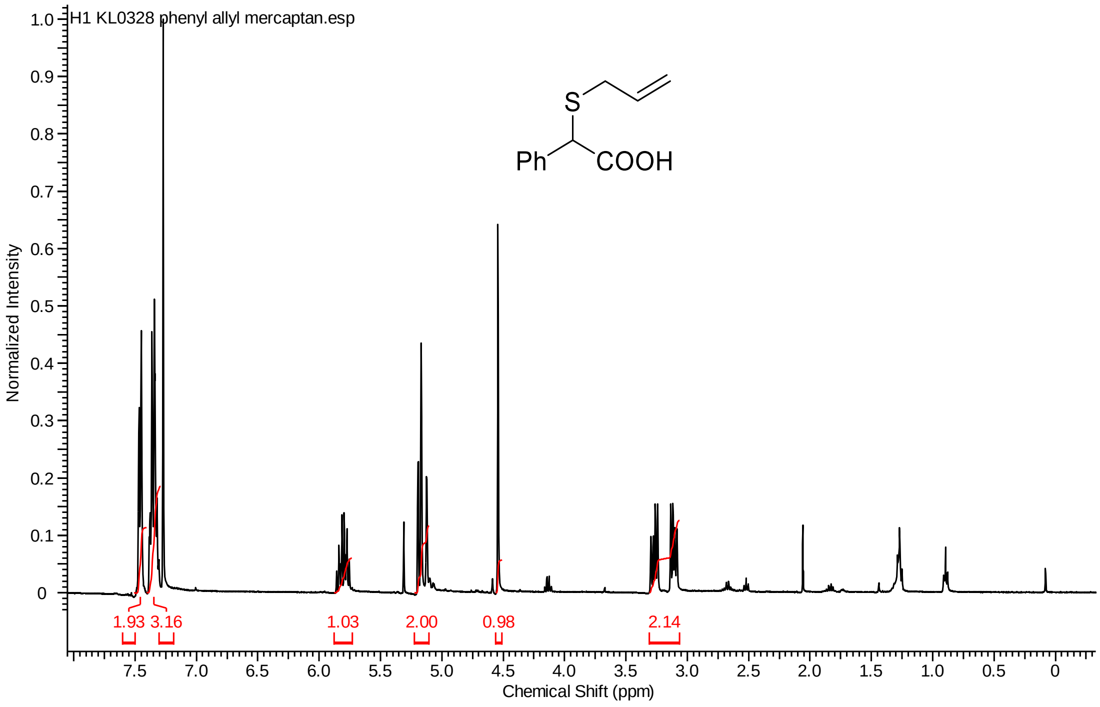

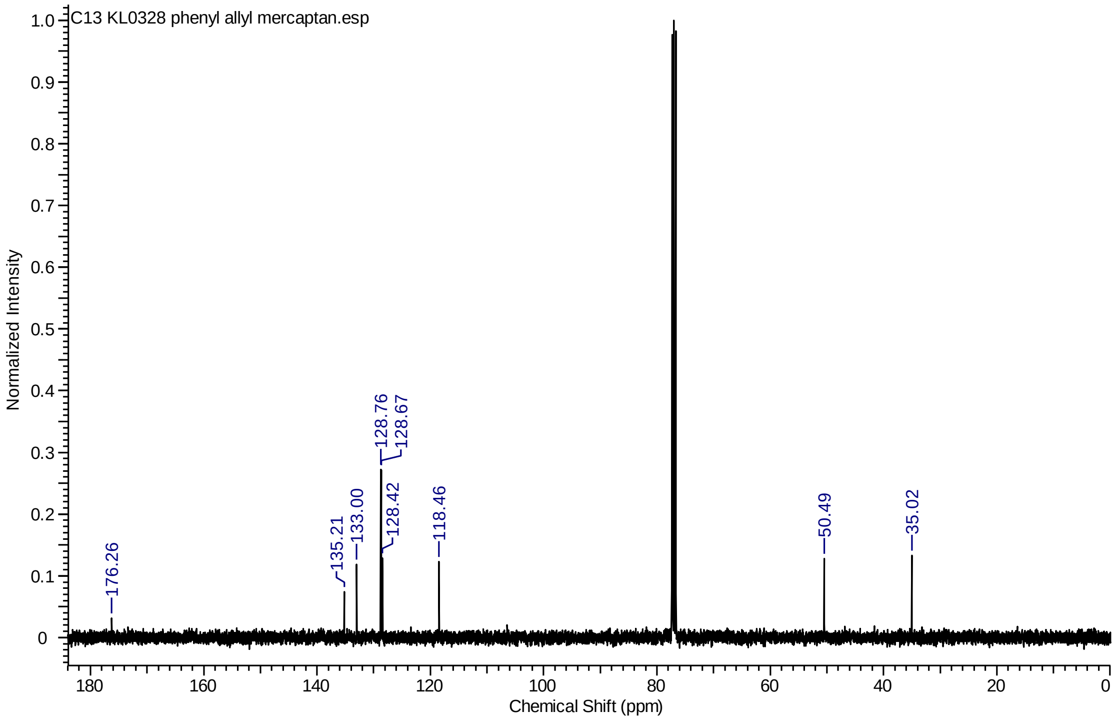

2-(Phenylthio)pentanoic acid 9k

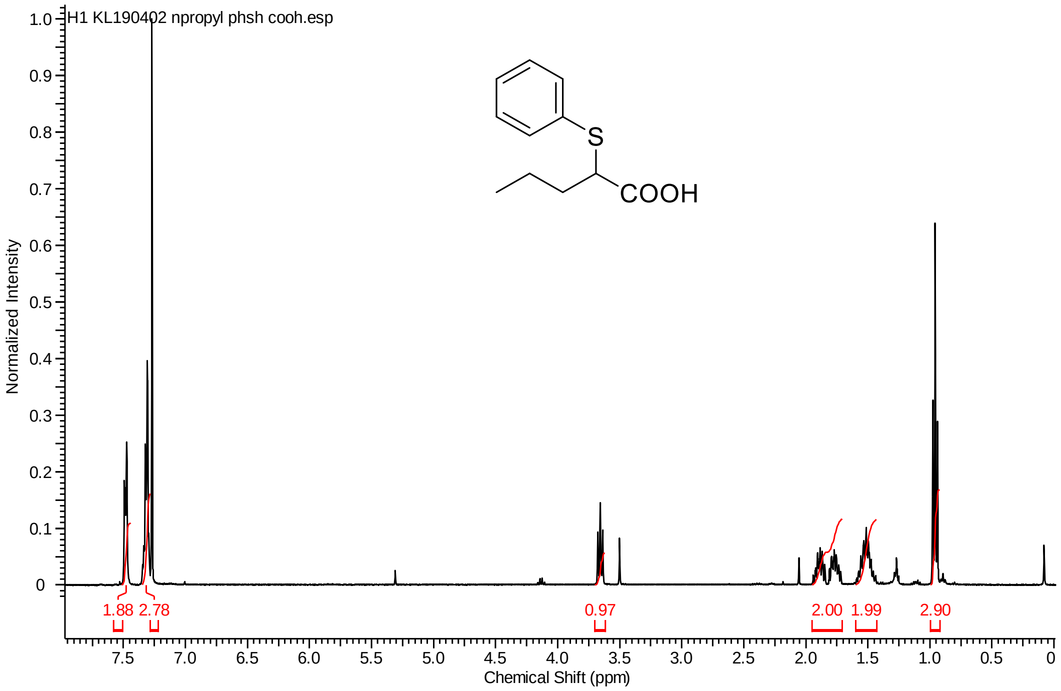

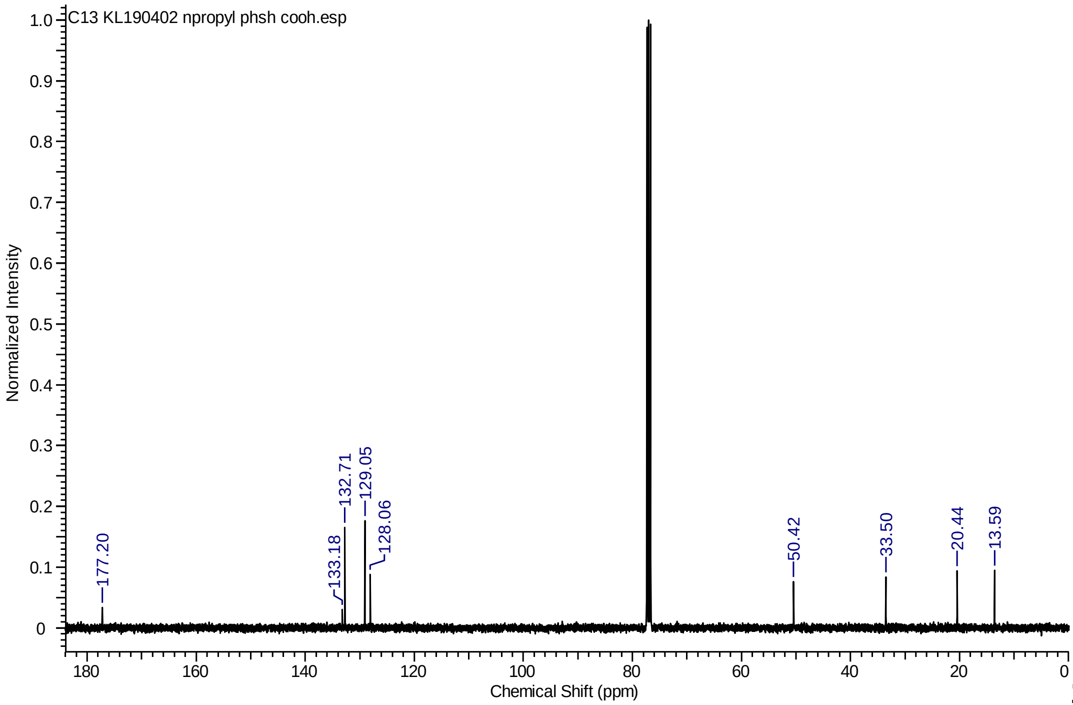

2-

2-(*p*-Tolylthio)pentanoic acid 9l

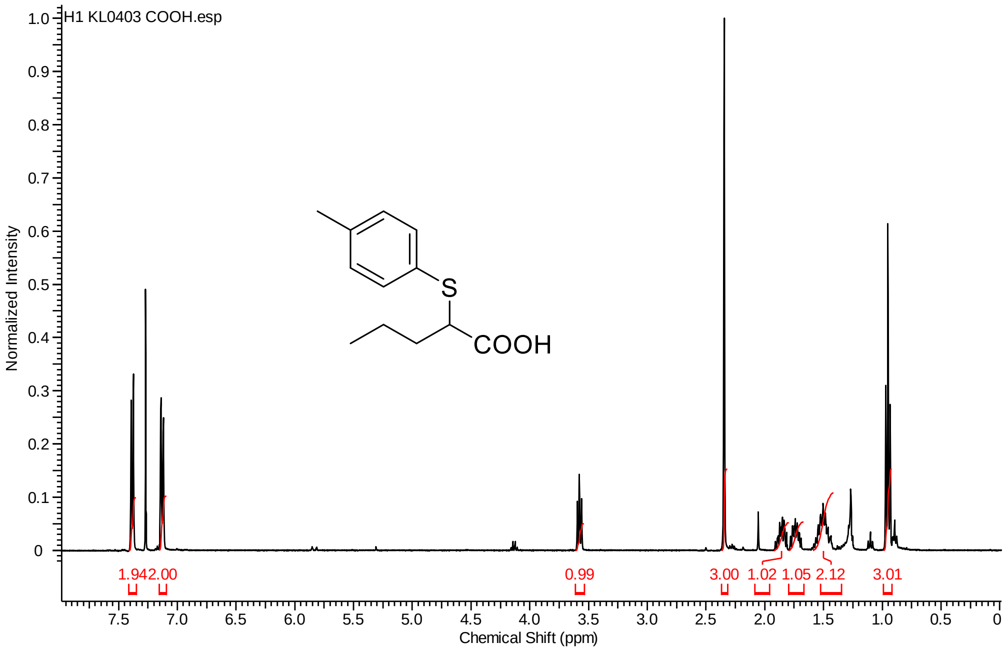
H1 KL0403 COOH.esp
S
COOH
1.94
2.00
0.99
3.00
1.02
1.05
2.12
3.01
Normalized Intensity
Chemical Shift (ppm)

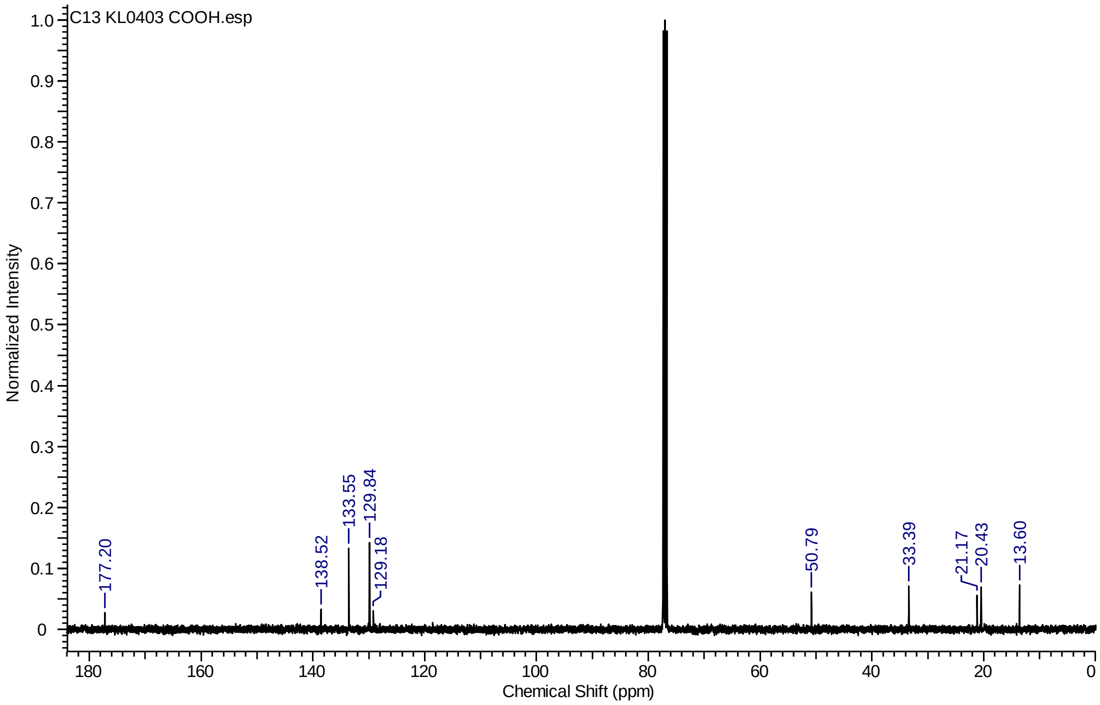
C13 KL0403 COOH.esp
177.20
138.52
133.55
129.84
129.18
50.79
33.39
21.17
20.43
13.60
Normalized Intensity
Chemical Shift (ppm)

3-Methyl-2-(phenylthio)butanoic acid 9m

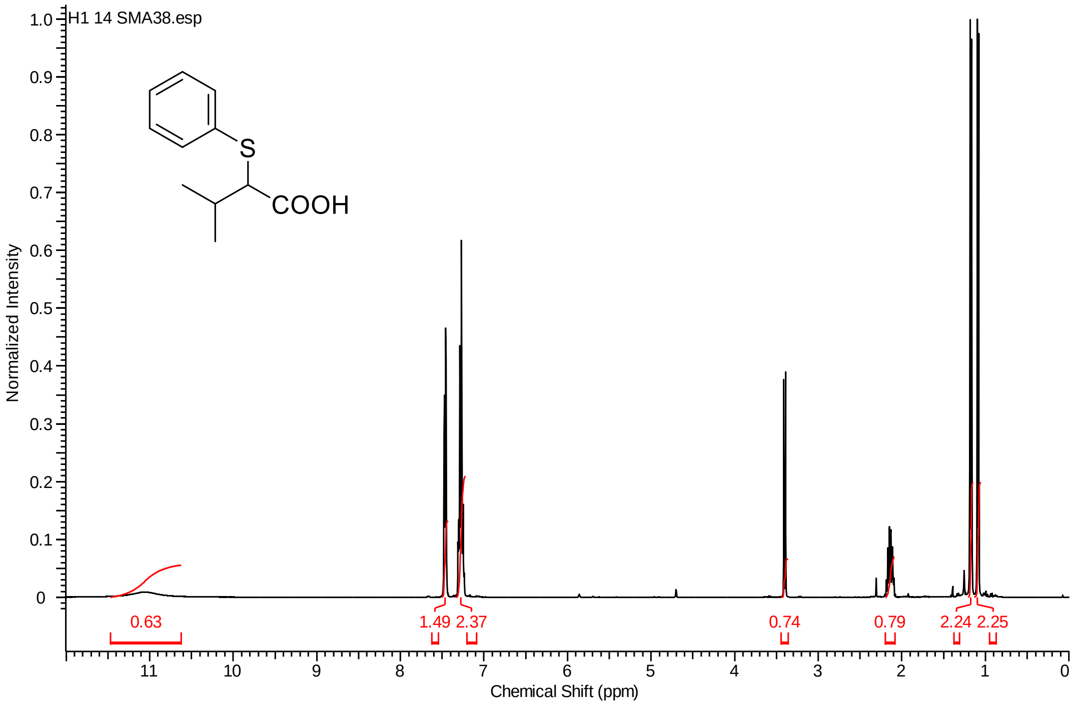

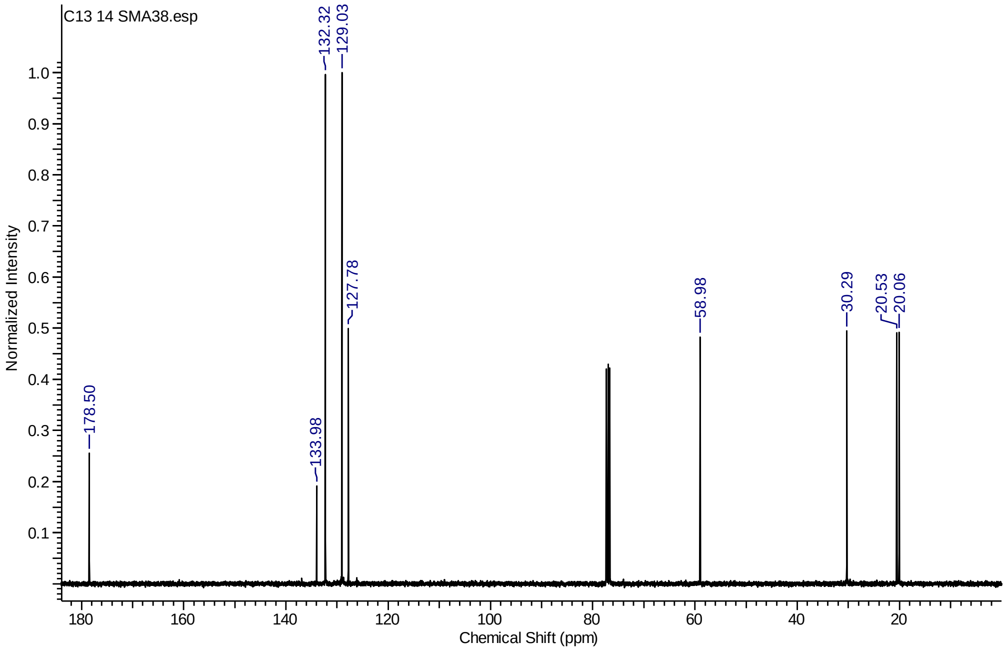

2-((4-Chlorophenyl)thio)pentanoic acid 9n

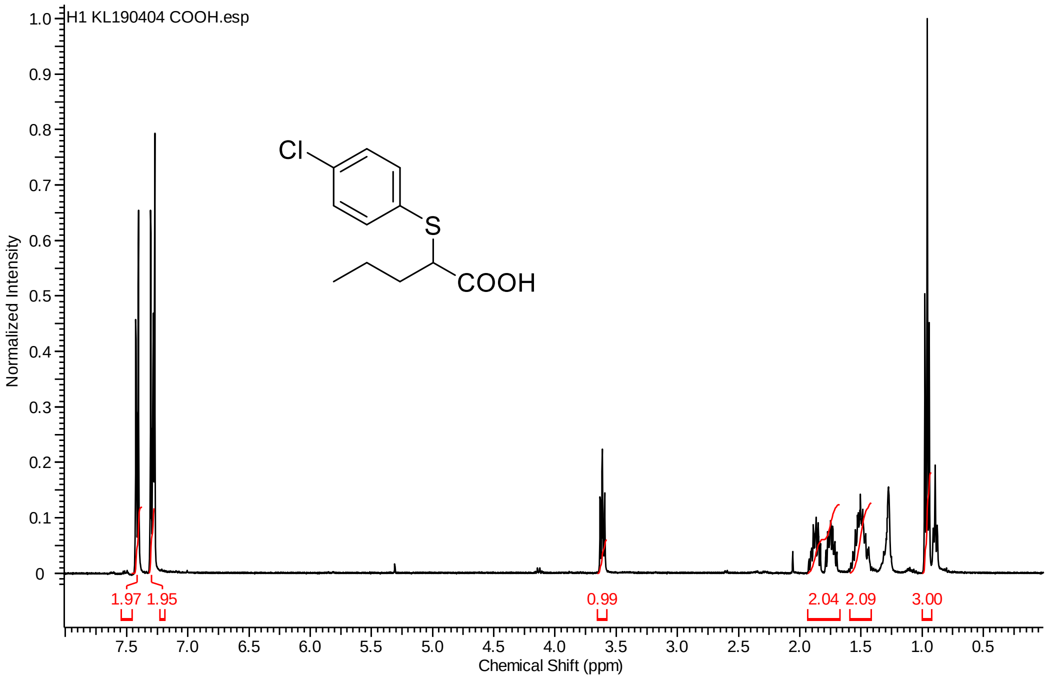

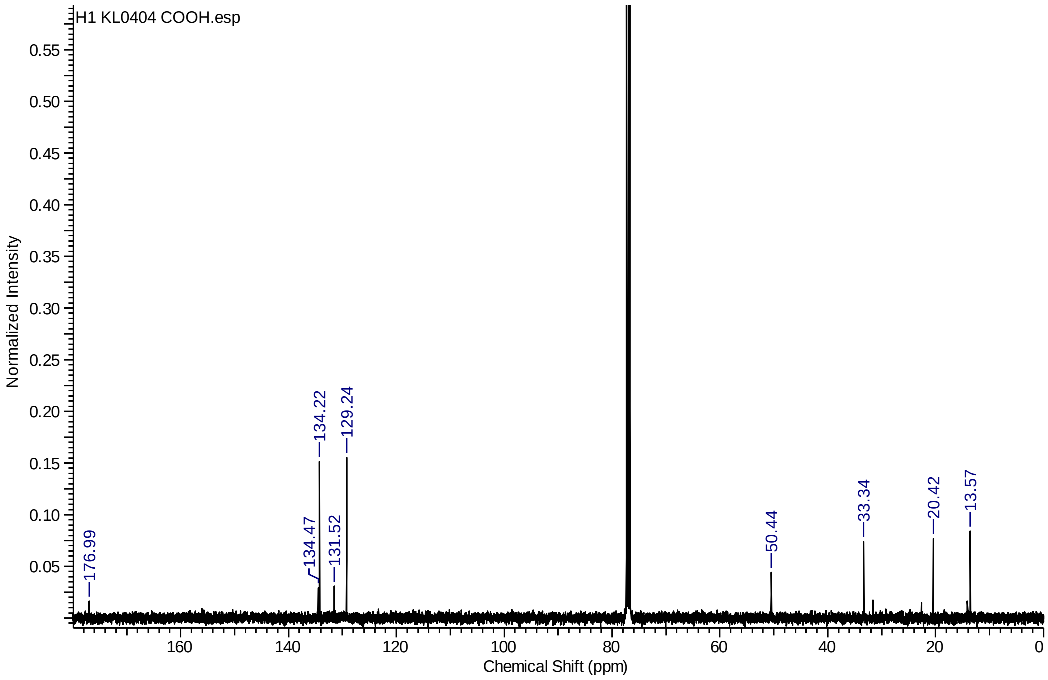

2-((4-Chlorophenyl)thio)-3-methylbutanoic acid 9o

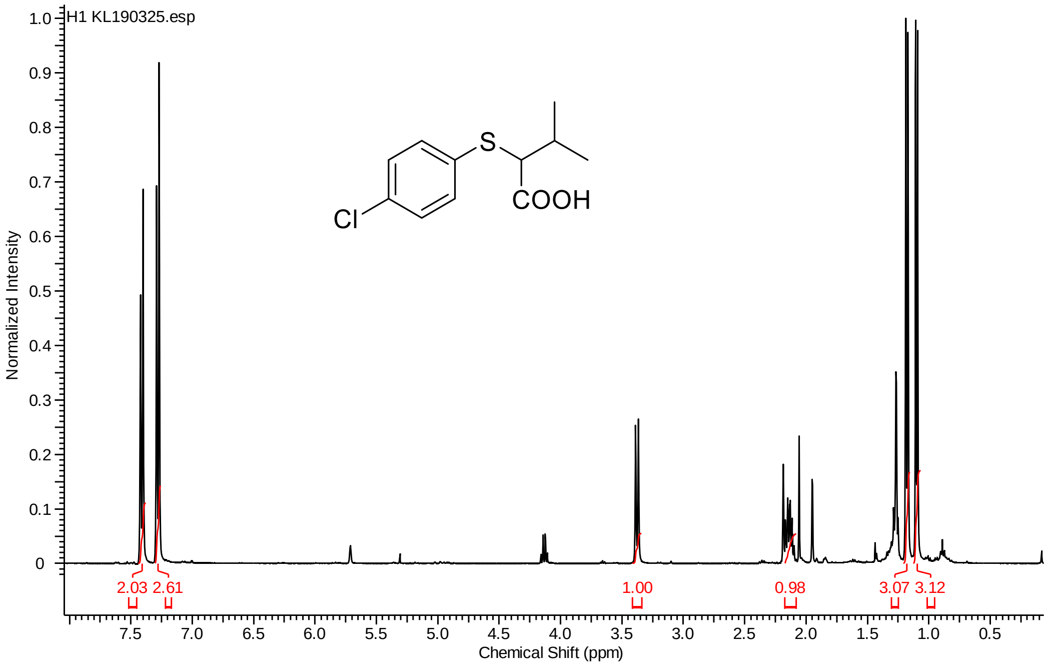

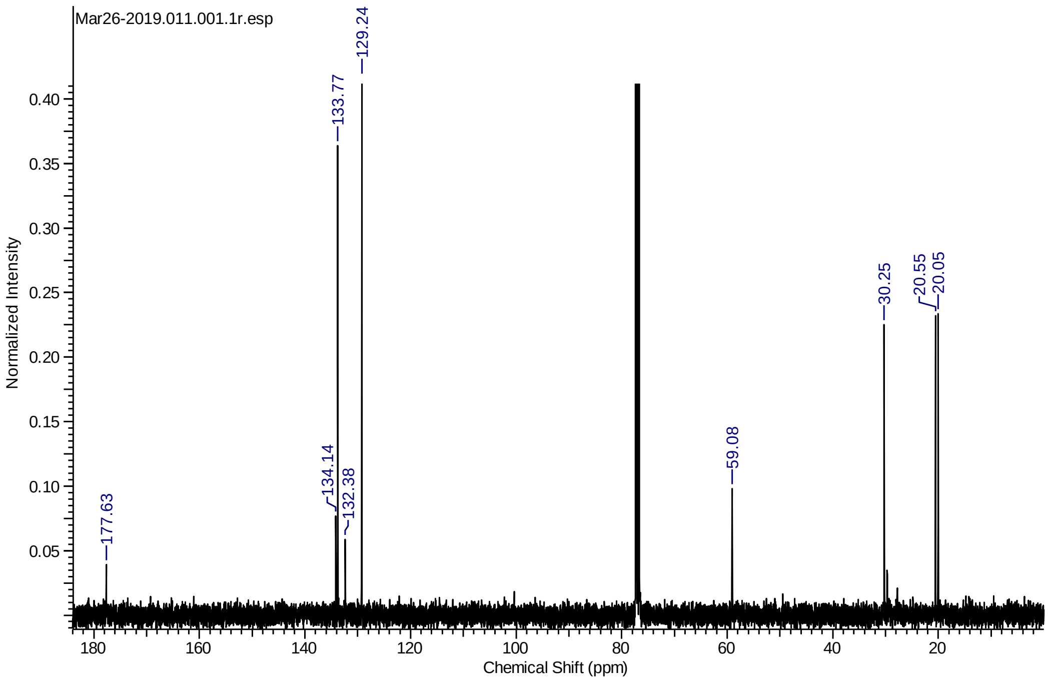

Phenyl-2-(propylthio)acetic acid 9p

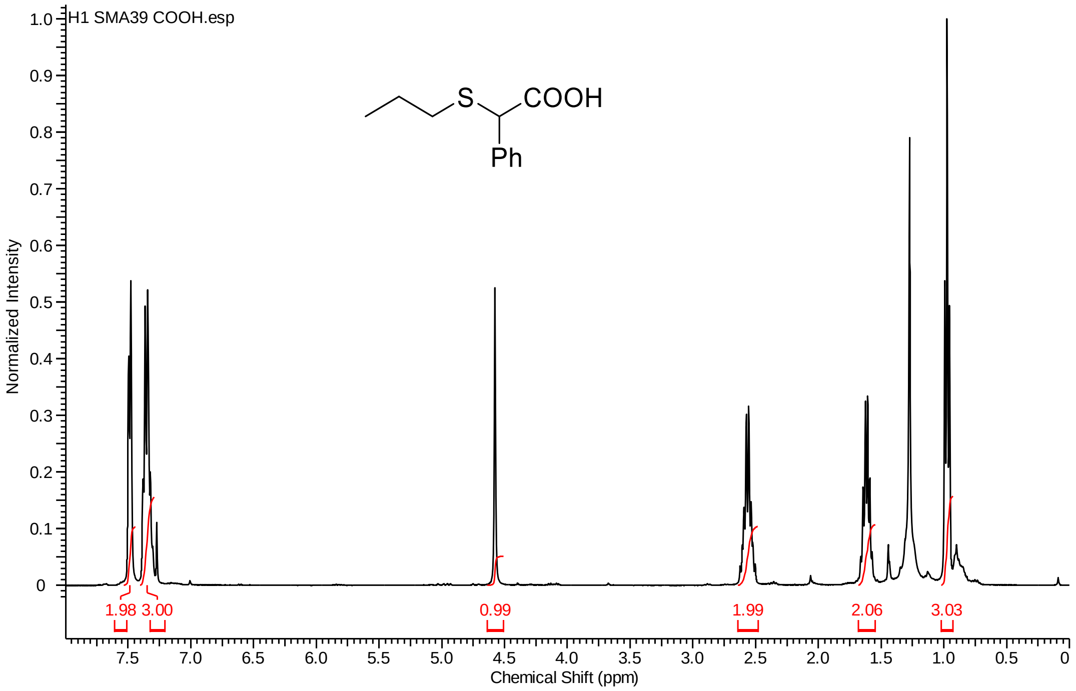

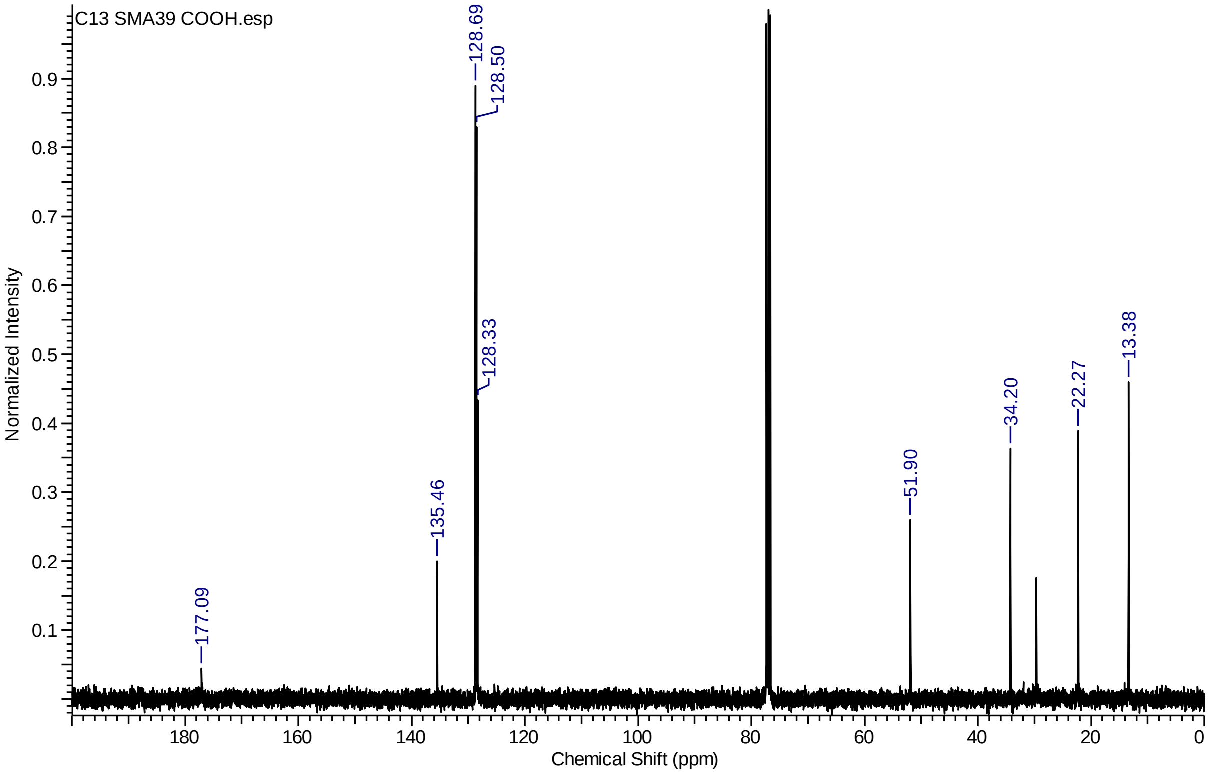

Supplement: Supplementary file 1 — Supplementary [file CHEM-26-10422-s001.pdf]
